# Sustainable multicomponent synthesis of C-4 sulfenylated pyrazoles *via* sodium thiosulfate-promoted tandem cyclocondensation and C–H sulfenylation

**DOI:** 10.1039/d5ra07282d

**Published:** 2025-10-27

**Authors:** Sanaz Abdollahi, Mohammad Abbasi, Najmeh Nowrouzi

**Affiliations:** a Department of Chemistry, Faculty of Nano and Bio Science and Technology, Persian Gulf University Bushehr 75169 Iran abbassi@pgu.ac.ir

## Abstract

We report a novel and environmentally benign multicomponent reaction (MCR) strategy for the synthesis of C-4 sulfenylated pyrazoles promoted by Na_2_S_2_O_3_·5H_2_O. This metal-free, one-pot protocol enables the simultaneous formation of two C–N bonds and one C–S bond through a cascade of cyclocondensation and direct C–H sulfenylation. A wide range of aryl and alkyl hydrazines and disulfides are efficiently converted to the corresponding products in good to excellent yields (72–94%) under sustainable conditions. DMSO plays a dual role as both the reaction medium and a modulator facilitating thiyl radical generation, which is essential for achieving regioselective C-4 sulfenylation. Compared to previously reported metal-catalyzed methods, this protocol offers high atom economy, broad substrate scope, and reduced environmental footprint, aligning well with the principles of green chemistry and advancing sustainable synthetic methodologies in heterocyclic chemistry.

## Abstract 2

A sustainable domino multicomponent protocol enables the synthesis of C-4 sulfenylated pyrazoles *via* simultaneous pyrazole ring construction and C–H sulfenylation in a single operation under mild conditions.

## Graphical abstract

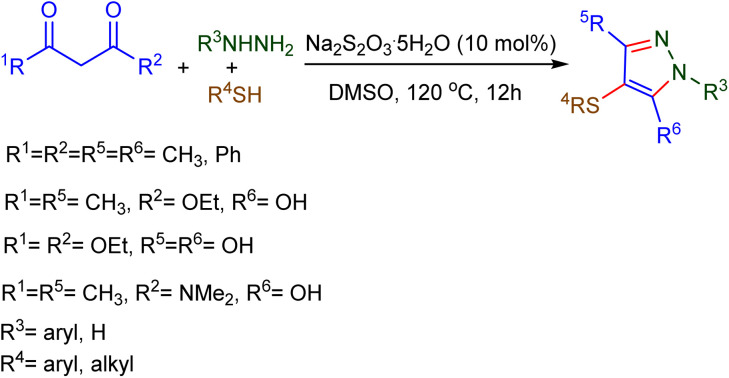

## 1 Introduction

The development of multicomponent domino C–H bond functionalization reactions has emerged as a powerful and atom-economical approach in modern organic synthesis, enabling the efficient construction of complex heterocycles from simple starting materials. This goal has been extensively achieved through transition-metal-catalysed systems, particularly those employing palladium and rhodium catalysts.^1,2^ In addition, transition-metal-free methodologies have recently emerged as attractive alternatives, often relying on radical-mediated pathways to accomplish selective C–H bond activation.^3–5^

Over the past decades, a variety of sulfur-based transformations^6^ such as sulfenylation,^7–14^ sulfinylation,^15,16^ and sulfonylation^17,18^ have been developed for the construction of C–S bonds, enabling access to structurally diverse organosulfur compounds. Among them, sulfenylation represents the most straightforward route for the formation of thioethers and related sulfur-containing motifs through the direct introduction of –SR groups.

Pyrazole derivatives have attracted considerable attention in pharmaceutical research due to their unique structural features and diverse biological properties. These heterocycles have exhibited a wide spectrum of bioactivities, including anticancer, anti-inflammatory, antibacterial, and antimicrobial effects. As a result, the development of efficient synthetic methodologies for the construction of pyrazole frameworks remains a topic of significant interest in organic synthesis.^19,20^

To date, various strategies have been established for pyrazole synthesis, encompassing metal-catalysed, metal-free, photochemical, and multicomponent reactions.^21^ Among metal-catalysed methods, notable examples include the silver-mediated [3 + 2] cycloaddition of *N*-isocyano imino triphenylphosphorane,^22^ AlCl_3_-promoted reactions of tosylhydrazones with terminal alkynes,^23^ ruthenium-catalysed dehydrogenative coupling of 1,3-diols with arylhydrazines,^24^ iron-catalysed coupling of diarylhydrazones with diols,^25^ and copper-catalysed oxidative cyclization of β,γ-unsaturated hydrazones.^26^ In contrast, metal-free approaches have also shown great promise, with representative examples including one-pot condensations of carbonyl compounds and hydrazines followed by *in situ* oxidation,^27^ iodine-mediated C–N oxidative cyclization of α,β-unsaturated carbonyls,^28^ and photoredox transformations under visible light using hydrazines and Michael acceptors.^29^

Incorporation of sulfur-containing moieties into heterocyclic compounds has emerged as a valuable strategy for modulating their biological activity. Accordingly, the formation of carbon–sulfur (C–S) bonds has become a crucial synthetic objective, particularly in the development of sulfur-functionalized pyrazoles. Traditionally, sulfenylated pyrazoles are accessed through direct sulfenylation of preformed pyrazoles *via* oxidative coupling with thiols as aryl sulfur sources.^30^ However, an alternative and more streamlined strategy involves one-pot multicomponent reactions, wherein pyrazole ring construction and sulfenylation occur in a single operation.

Several such methodologies have recently been reported, utilizing diverse substrates and catalytic systems. For example, an iodine-catalysed three-component reaction of 1,3-diketones, hydrazines, and thiols has been employed to synthesize C4-sulfenylated pyrazoles.^31^ Similarly, a [3 + 2] annulation involving β-ketonitriles (or pentane-2,4-dione), arylhydrazines, and arylsulfonyl hydrazides under iodine mediation has been developed,^32^ while a NIS-promoted three-component reaction of 3-oxo-3-arylpropanenitriles and arylsulfonyl hydrazides affords 3-aryl-4-(arylthio)-1*H*-pyrazol-5-amines through sequential cyclization and sulfenylation.^33^ AlCl_3_-mediated chalcogenation/cyclization of α,β-alkyl hydrazones provides access to 4-chalcogenyl pyrazoles.^34^ Furthermore, cascade reactions starting from phenylhydrazine, thiols, and aminoacrylonitriles offer another practical route to sulfenylated pyrazoles.^35^ A domino cyclization and C–H sulfenylation strategy using 1,3-diarylpropane-1,3-diones or 1,3-diketones and arylsulfonyl hydrazides has also been described, relying on iodide-based catalysts such as NIS and NH_4_I.^36^

Despite their efficacy, many of these methods rely on reactive halogen sources or strong Lewis acids, which may raise concerns regarding safety, volatility, and environmental impact. To address these issues, we turned our attention to sodium thiosulfate (Na_2_S_2_O_3_), a stable, inexpensive, and non-volatile reagent with well-documented nucleophilic and mild reducing properties. It is widely used in organic synthesis for introducing sulfur atoms,^37–40^ quenching excess oxidants,^41–43^ and enabling chemoselective transformations with excellent functional group compatibility.^44,45^

**Scheme 1:**
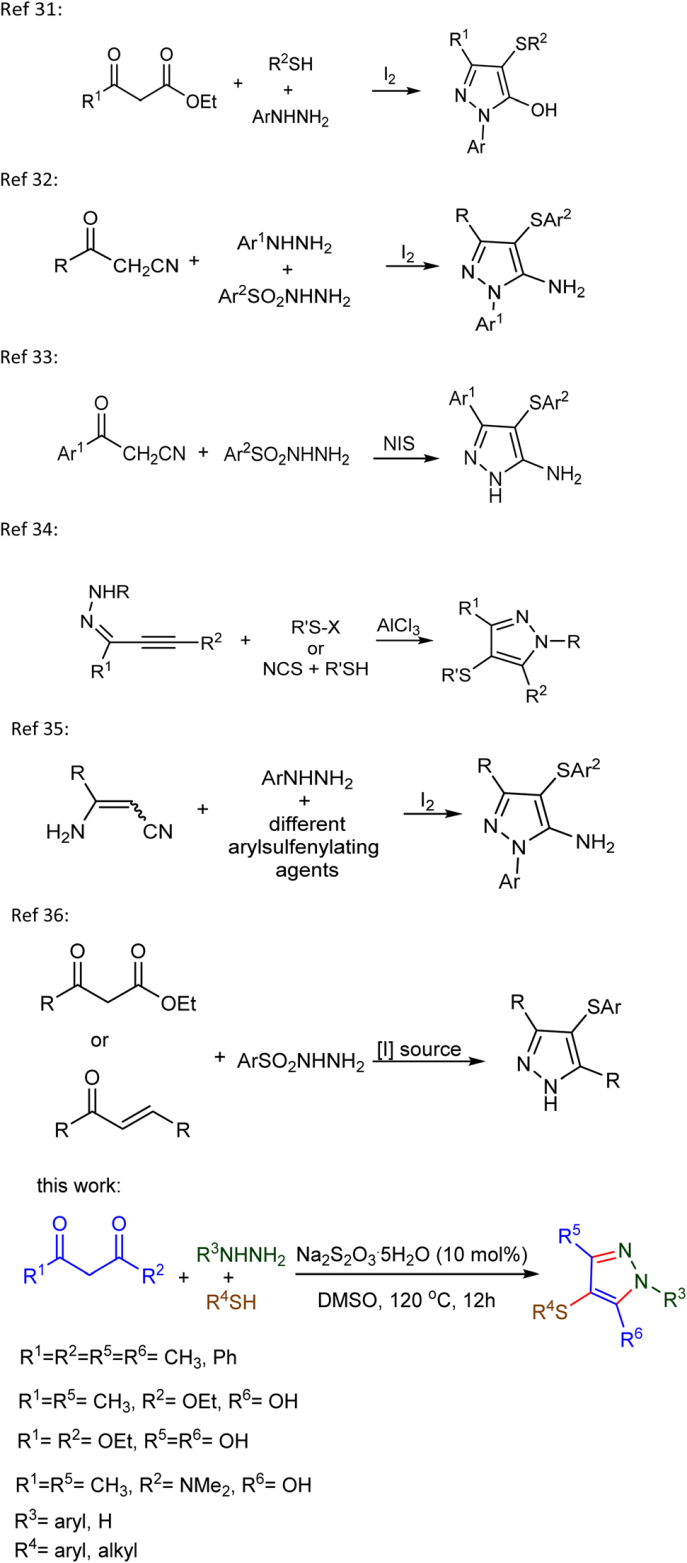
Methods for synthesis of thiol-substituted pyrazoles.

Encouraged by our previous success in using Na_2_S_2_O_3_ to catalyse the sulfenylation of enaminones with thiols^46^ also, decarbonylative sulfenylation and decarboxylative sulfenylation of aldehydes and carboxylic acids by thiols,^47^ we sought to expand its application to the synthesis of sulfenylated pyrazoles. Herein, we report a practical and environmentally benign one-pot method for constructing 4-sulfenylpyrazoles from 1,3-diketones or β-keto esters, hydrazine derivatives, and thiols, using Na_2_S_2_O_3_ as a catalyst. This approach highlights the potential of sodium thiosulfate as a green alternative to traditional halogen-based promoters in sulfur-containing heterocycle synthesis through C–H bond activation reaction (Scheme 1).

## 2 Experimental

### 2.1. General remarks

All commercially available reagent-grade chemicals were purchased from chemical suppliers and used as received without further purification. Proton and carbon magnetic resonance spectra (^1^H NMR and ^13^C NMR) were recorded using either tetramethylsilane (TMS) as the internal standard in CDCl_3_ (^1^H NMR: TMS at 0.00 ppm, CDCl_3_ at 7.24 ppm; ^13^C NMR: CDCl_3_ at 77.0 ppm) or tetramethylsilane (TMS) as the internal standard in DMSO-d_6_ (^1^HNMR: TMS at 0.00 ppm, DMSO at 2.50 ppm; ^13^C NMR: DMSO at 40.0 ppm). The chemical shifts (d) were expressed in ppm and *J* values were given in Hz. The following abbreviations are used to indicate the multiplicity: singlet (s), doublet (d), triplet (t) and multiplet (m). All first order splitting patterns were assigned on the basis of the appearance of the multiplet. Splitting patterns that could not be easily interpreted were designated as multiplet (m).

### 2.2. General procedure for the synthesis of substituted C4-sulfenylated pyrazoles

A stoppered test tube equipped with a magnetic stirring bar was charged with Na_2_S_2_O_3_·5H_2_O (0.025 g, 0.1 mmol), a substituted 1,3-diketone (1.0 mmol), hydrazine or a hydrazine derivative (1.0 mmol), thiol (1.0 mmol), and DMSO (2.0 mL). The mixture was stirred at 120 °C under an air atmosphere for 12 hours. After this period, the reaction mixture was allowed to cool to room temperature and then diluted by the addition of distilled water (1.0 mL). The mixture was extracted with ethyl acetate (5 × 2 mL), and the combined organic layers were concentrated. The crude product was purified by silica gel column chromatography using an appropriate mixture of *n*-hexane and ethyl acetate to afford the pure C4-sulfenylated pyrazoles.

## 3 Results and discussion

In the initial phase of this study acetylacetone (1a), phenylhydrazine (2a), and thiophenol (3a) were selected as model substrates, and Na_2_S_2_O_3_·5H_2_O was employed as the catalyst to optimize reaction conditions. The results of these optimization experiments are summarized in Table 1.

**Table 1:** Optimization of the reaction conditions^a^

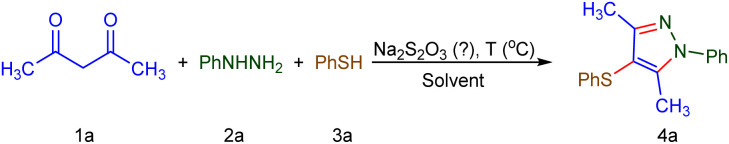				
Entry	Na_2_S_2_O_3_·5H_2_O (mmol)	Solvent	Temp. [°C]	Yield^b^ (%)
1	—	DMSO	80	15
2	0.1	DMSO	80	50
3	0.1	DMF	80	28
4	0.1	H_2_O	80	30
5	0.1	CH_3_CN	80	10
6	0.1	EtOH	80	15
7	0.1	MeOH	80	15
8	0.1	—	80	10
9	0.1	DMSO	90	70
10	0.1	DMSO	100	79
**11**	**0.1**	**DMSO**	**120**	**88**
12	0.1	DMSO	140	88
13	0.2	DMSO	120	88

a Reaction conditions: 1a (1.0 mmol), 2a (1.0 mmol), 3a (1.0 mmol), solvent (2.0 mL), 12 h.

b Isolated yield.

A variety of solvents were evaluated to determine their effect on the reaction efficiency, including polar aprotic solvents such as dimethyl sulfoxide (DMSO), *N*,*N*-dimethylformamide (DMF), and acetonitrile (CH_3_CN), as well as polar protic solvents such as ethanol (EtOH), methanol (MeOH), and water (H_2_O). Among them, DMSO proved to be the most effective medium, delivering the highest yield at 80 °C (Table 1, entry 2). In contrast, significantly lower yields were observed with the other solvents, indicating the superior solvating and stabilizing capabilities of DMSO in this transformation. Moreover, in the absence of the catalyst, only trace amounts of product were detected (entry 1), underscoring the essential role of Na_2_S_2_O_3_·5H_2_O.

Evaluation of reaction temperature further revealed that 120 °C provided the best outcome (entry 11), while increasing the temperature or catalyst loading beyond this point had no beneficial effect (entries 12 and 13). Based on these findings, the optimal conditions were determined to be 0.1 equiv. of Na_2_S_2_O_3_·5H_2_O in 2.0 mL of DMSO at 120 °C.

With the optimized conditions in hand, the substrate scope and limitations of the reaction were systematically explored using a diverse array of thiols, 1,3-diketones, and hydrazines. The diketone substrates include acetylacetone (1a) and 1,3-diphenylpropane-1,3-dione (1b), while the hydrazine derivatives employed are phenylhydrazine (2a), 2-chlorophenylhydrazine (2b), 4-bromophenylhydrazine (2c), and hydrazine (2d). A range of thiols were also investigated, including thiophenol (3a), *p*-methylthiophenol (3b), *p*-fluorothiophenol (3c), *p*-chlorothiophenol (3d), 2-mercaptobenzoic acid (3e), benzyl mercaptan (3f), naphthalene-1-thiol (3g), hexane-1-thiol (3h), butane-2-thiol (3i), furan-2-ylmethanethiol (3j), and ethane-1,2-dithiol (3k). The results are presented in Table 2.

**Table 2:** Three-component synthesis of 4-aryl(-alkyl)thiopyrazoles^a^^,^^b^

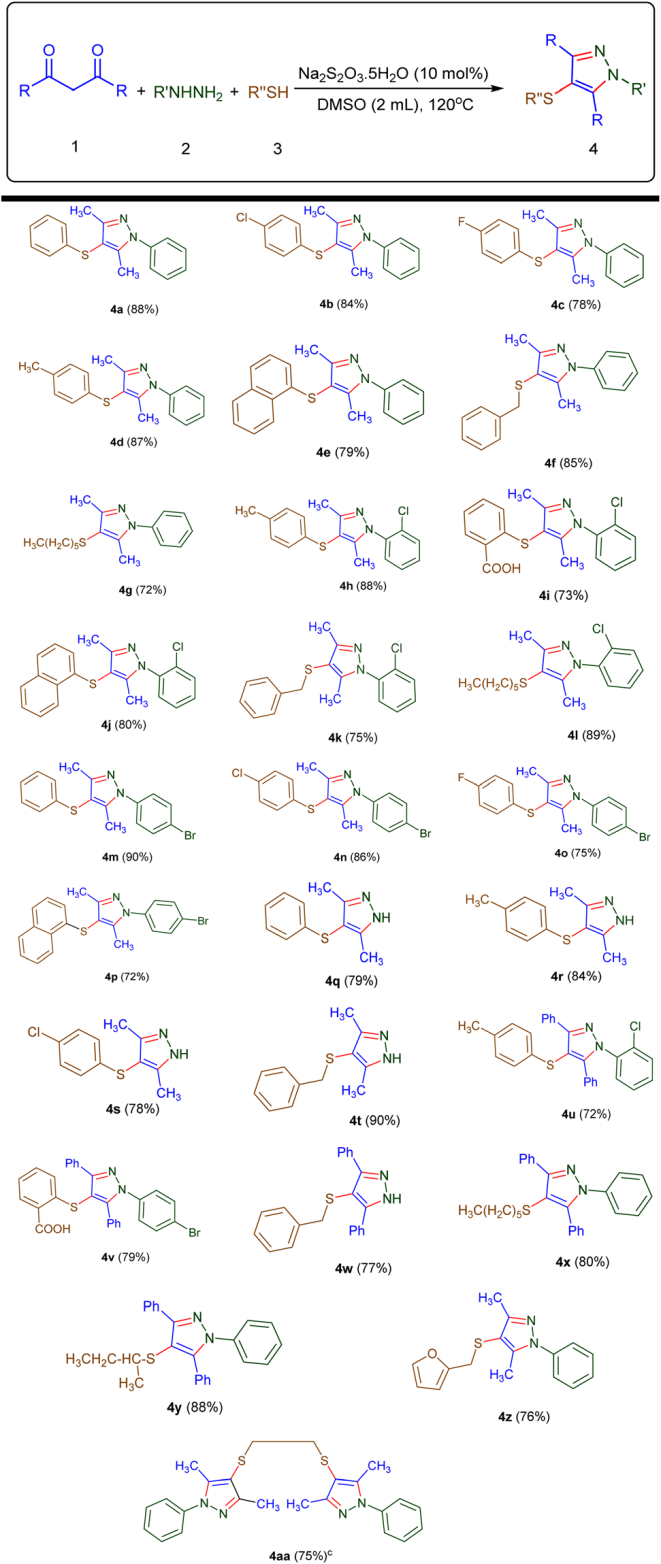

a Reaction conditions: 1 (1.0 mmol), 2 (1.0 mmol), 3 (1.0 mmol), Na_2_S_2_O_3_·5H_2_O (10 mol%), DMSO (2.0 mL), at 120 °C, 12 h.

b Isolated yields of the products are indicated in parentheses next to their respective labels.

c Reactants 1, 2, and 3 were used in a molar ratio of 2.0 : 2.0 : 1.0 (mmol).

As summarized in Table 2, the three-component reaction demonstrated a broad substrate scope and excellent functional group tolerance under the optimized conditions. In general, the reaction exhibited excellent tolerance toward both electron-donating and electron-withdrawing substituents on aromatic thiols. For instance, thiophenol (3a), *p*-methylthiophenol (3b), and *p*-chlorothiophenol (3d) reacted efficiently with acetylacetone (1a) and phenylhydrazine (2a), affording the corresponding sulfenylated pyrazoles (4a, 4d, and 4b) in high yields (88%, 87%, and 84%, respectively). Similarly, *p*-fluorothiophenol (3c) gave satisfactory results (4c, 78%). Notably, 2-mercaptobenzoic acid (3e), despite its potential steric hindrance, successfully afforded the desired product 4i in 73% yield.

Naphthalene-1-thiol (3g), as a polycyclic aromatic thiol also participated well in the transformation, delivering the corresponding products (4e, 4j, and 4p) with yields ranging from 72% to 80%.

Among the non-aromatic thiols, both benzyl mercaptan (3f) and furan-2-ylmethanethiol (3j) demonstrated high reactivity under the optimized conditions. Benzyl mercaptan (3f), as a representative benzylic thiol, afforded the desired sulfenylated pyrazoles (4f, 4k, and 4t) in good to excellent yields ranging from 75% to 90% across different hydrazine partners. Similarly, furan-2-ylmethanethiol (3j), as a heterobenzylic thiol, successfully furnished the corresponding product 4z in 76% yield. These results suggest that both benzylic and heterobenzylic thiols are well-suited to this transformation.

Aliphatic thiols, including both linear and branched derivatives such as hexane-1-thiol (3h), butane-2-thiol (3i), and ethane-1,2-dithiol (3k), also engaged effectively in the reaction. The corresponding sulfenylated products (4g, 4x, 4y, and 4aa) were obtained in good to excellent yields (72–88%), demonstrating the versatility of the protocol across structurally diverse thiol classes.

With respect to the hydrazine component, arylhydrazines bearing halogens (2b–2c) as well as unsubstituted hydrazine (2d) were all competent coupling partners, providing a wide array of functionalized pyrazoles. The electronic nature of substituents on hydrazines did not significantly impact the reaction efficiency. Furthermore, extending the diketone backbone from acetylacetone (1a) to the more sterically demanding 1,3-diphenylpropane-1,3-dione (1b) did not hinder the reaction outcome, as evidenced by the successful synthesis of products 4u–4x in yields ranging from 72% to 88%.

Overall, the method demonstrated excellent generality and robustness across a structurally and electronically diverse substrate scope, offering a straightforward and environmentally friendly approach to the synthesis of sulfenylated pyrazoles.

Following the successful synthesis of sulfenylated pyrazoles from 1,3-diketones, we aimed to further broaden the applicability of the reaction to other synthetically valuable carbonyl compounds. Accordingly, ethyl acetoacetate (5a), diethyl malonate (5b), and *N*,*N*-dimethyl-3-oxobutanamide (5c) were selected as alternative substrates to assess their compatibility under the optimized conditions (Table 3).

**Table 3:** Synthesis of C-4 sulfenylated pyrazoles using (5a–c)^a^^,^^b^

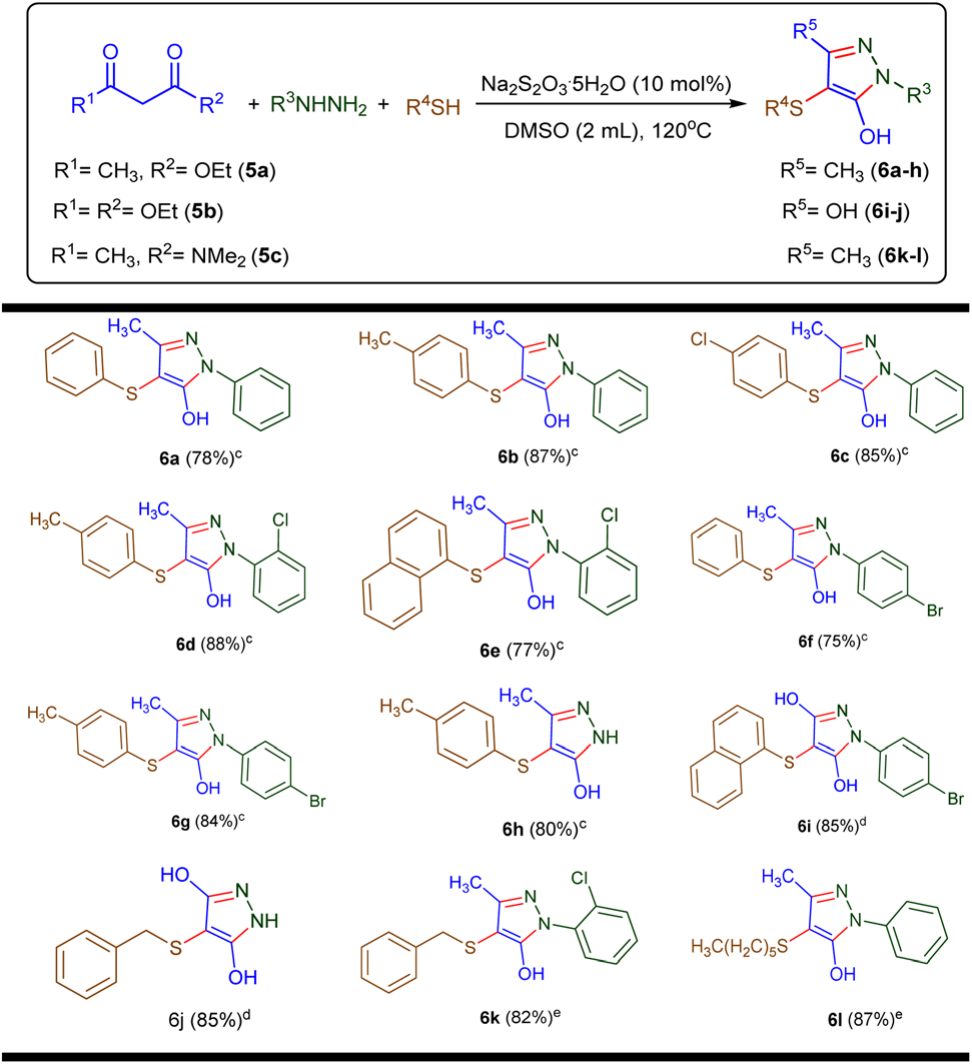

a Reaction conditions: 5 (1.0 mmol), 2 (1.0 mmol), 3 (1.0 mmol), Na_2_S_2_O_3_·5H_2_O (10 mol%), DMSO (2.0 mL), at 120 °C, 12 h.

b Isolated yields of the products are indicated in parentheses next to their respective labels.

c The products were obtained from substrate 5a.

d The products were obtained from substrate 5b.

e The products were obtained from substrate 5c.

As summarized in Table 3, all three substrates participated smoothly in the transformation, affording the corresponding C-4 sulfenylated pyrazoles (6a–6l) in good to excellent yields (75–88%). Ethyl acetoacetate (5a) reacted efficiently with a variety of thiols and hydrazines. For instance, combination with thiophenol (3a) and phenylhydrazine (2a) yielded 6a in 78% yield, while reactions with *p*-methylthiophenol (3b) or *p*-chlorothiophenol (3d) furnished 6b and 6c in 87% and 85% yields, respectively. Substituted hydrazines also performed well: 4-chlorophenylhydrazine (2b) and 4-bromophenylhydrazine (2c) provided products 6d–6g in 75–88% yields depending on the thiol used. Notably, unsubstituted hydrazine (2d) afforded 6h in 80% yield when coupled with 3b and 5a.

Further exploration using diethyl malonate (5b) demonstrated that bis-ester systems are also amenable to this transformation. Products 6i and 6j were obtained in high yields (both 85%) using different hydrazine and thiol partners, highlighting the method's versatility.

In the case of *N*,*N*-dimethyl-3-oxobutanamide (5c), the reaction also proceeded efficiently. Products 6k and 6l were isolated in 82% and 87% yields, respectively, confirming the compatibility of amide-functionalized β-keto compounds, which are typically less reactive due to conjugative stabilization.

Collectively, these results underscore the broad functional group tolerance and general applicability of the method across diverse carbonyl frameworks-including β-ketoesters, malonates, and β-ketoamides-enabling access to a wide array of structurally diverse C-4 sulfenylated pyrazoles.

**Scheme 2:**
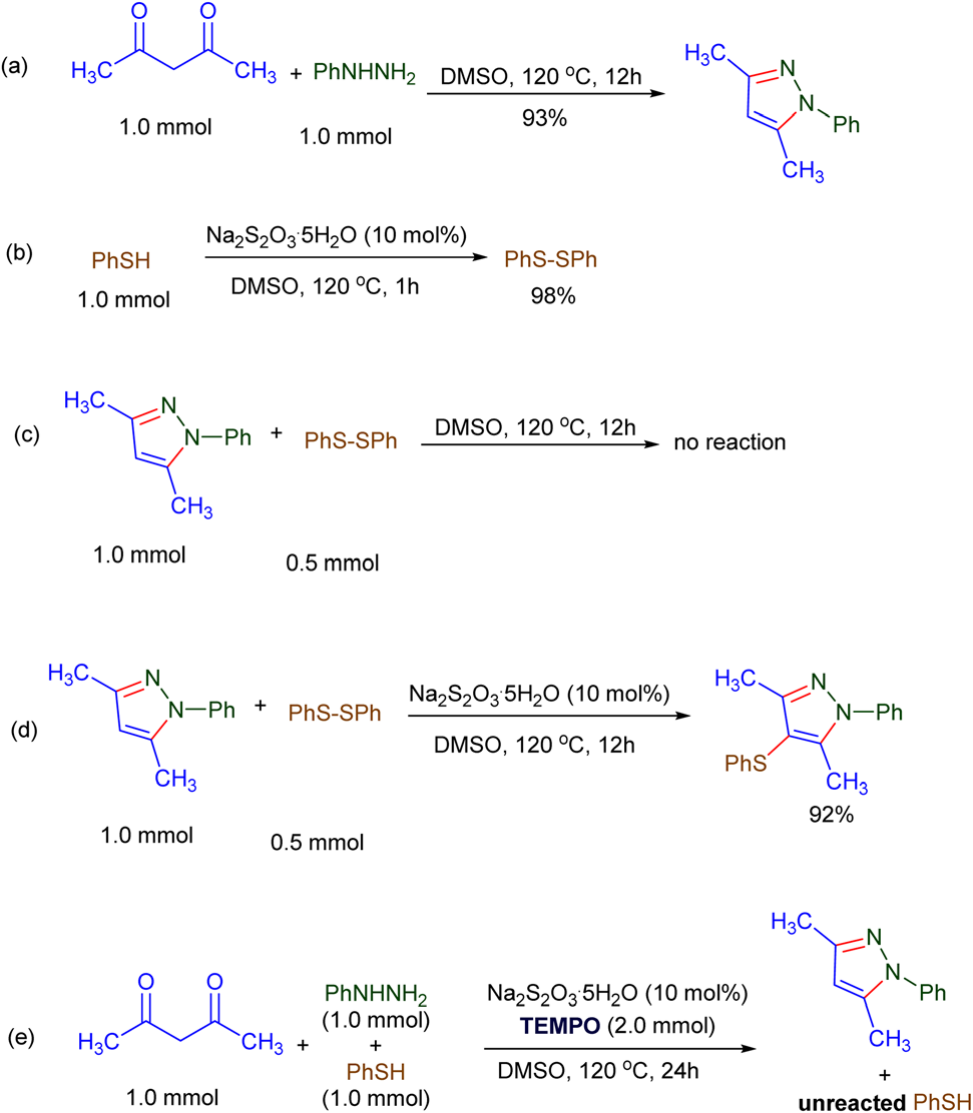
Control experiments.

In continuation, to gain deeper insight into the reaction process, several control experiments were performed (Scheme 2). First, to examine whether the formation of pyrazoles from 1,3-dicarbonyl compounds and hydrazines required catalysis by sodium thiosulfate, a reaction between acetylacetone and phenylhydrazine was carried out in DMSO at 120 °C (Scheme 2a). After 12 h, complete consumption of the substrates and formation of the corresponding pyrazole were observed, indicating that pyrazole formation is not necessarily dependent on sodium thiosulfate.

In another experiment, thiophenol was heated in DMSO in the presence of sodium thiosulfate for 1 h (Scheme 2b). Analysis of the reaction mixture revealed complete conversion of thiophenol into the corresponding disulfide.

Furthermore, to clarify the role of sodium thiosulfate in the sulfenylation of pyrazoles, a reaction between 3,5-dimethyl-1-phenyl-1*H*-pyrazole and diphenyl disulfide was investigated. Initially, the reaction was carried out in the absence of sodium thiosulfate (Scheme 2c), and after 12 h, both substrates remained intact. However, upon addition of sodium thiosulfate to the reaction mixture, the transformation proceeded smoothly to afford the desired sulfenylated product in 92% yield (Scheme 2d).

Finally, to confirm the involvement of radical intermediates in the sulfenylation step, the model reaction was carried out in the presence of the radical scavenger TEMPO (2.0 equiv.) under otherwise identical conditions (Scheme 2e). In this case, the formation of the sulfenylated product was completely inhibited; the thiol starting material was largely recovered and only the cyclocondensation product derived from acetylacetone and phenylhydrazine was obtained, even after 24 h. These results clearly indicate that sulfur-centered radicals are responsible for the C–S bond formation, thus supporting a radical-mediated mechanism for this transformation.

**Scheme 3:**
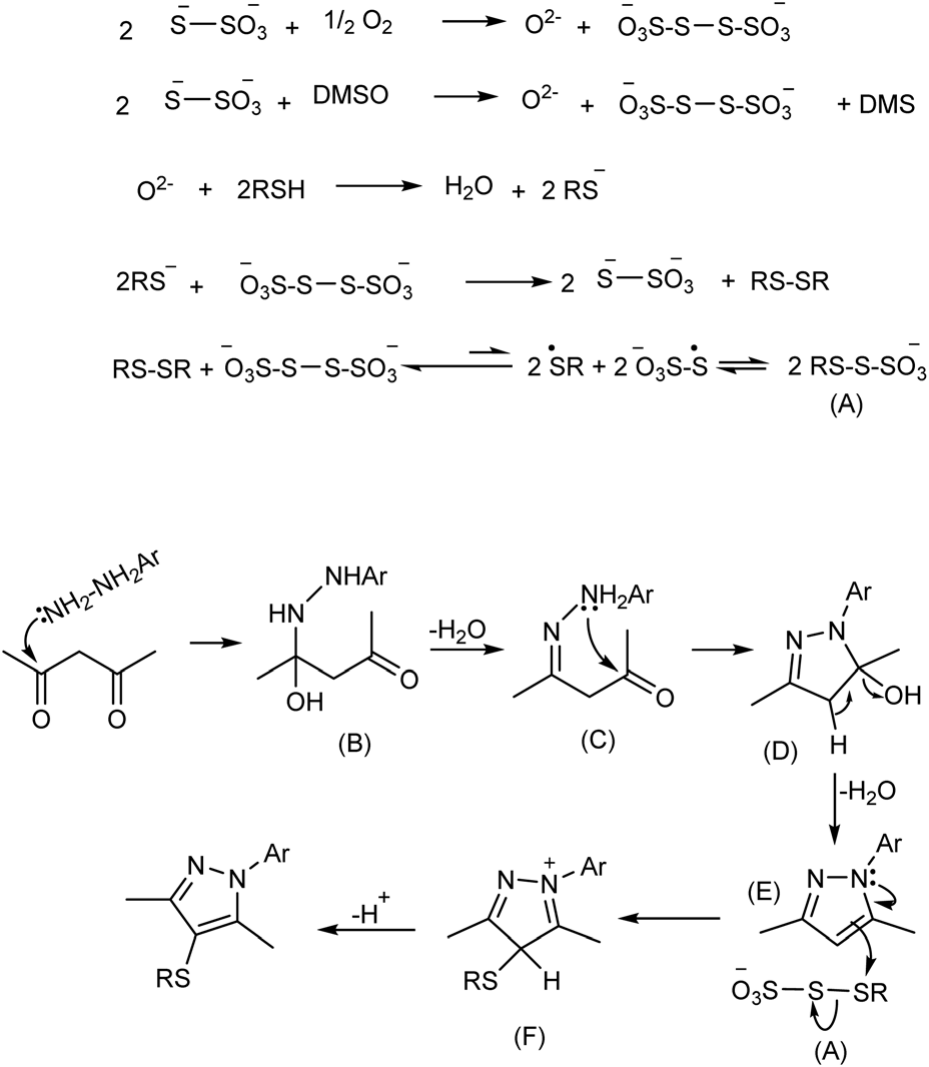
A proposed mechanism for the direct transformation.

Although the precise role of sodium thiosulfate in this transformation remains ambiguous, a plausible reaction mechanism is proposed in Scheme 3. DMSO, leading to the formation of tetrathionate (S_4_O_6_^2−^) and oxide anions (O^2−^). In this context, DMSO plays a dual role in the transformation. Besides serving as the solvent, it also acts as a mild oxidant under thermal conditions, facilitating the generation of tetrathionate intermediate. The strongly basic oxide anion can deprotonate the thiol to afford water and a thiolate anion.

Subsequent oxidation of the thiolate leads to the formation of a symmetrical disulfide (RS–SR), while tetrathionate is reduced back to thiosulfate, establishing a redox cycle. Given that the S–S bond in both disulfides and tetrathionate is relatively weak, it may undergo homolytic cleavage under the reaction conditions, producing thiyl (RS˙) radical. In the case of tetrathionate, such cleavage generates sulfur-centered thiosulfonyl [–O_3_S–S˙] radical.

We hypothesize that recombination of these radical species may lead to the formation of a transient sulfenylating intermediate (designated as species A), which is responsible for the electrophilic C–S bond formation at the C-4 position of the pyrazole ring.

## 4 Conclusions

We have developed an efficient and environmentally benign metal-free protocol for the synthesis of C-4 sulfenylated pyrazoles *via* a Na_2_S_2_O_3_·5H_2_O-catalyzed domino multicomponent reaction. This one-pot process enables the direct coupling of 1,3-dicarbonyl compounds, hydrazines, and thiols, leading to the simultaneous formation of two C–N bonds and one C–S bond in a single operational step.

The use of sodium thiosulfate as a mild, non-toxic, and green catalyst plays a crucial role in facilitating the reaction, offering advantages such as commercial availability, low cost, and environmental compatibility. Despite the reaction being carried out at 120 °C for 12 h, these conditions are necessary to efficiently promote both the cyclocondensation and C–H sulfenylation steps. The overall process remains sustainable due to its metal-free, additive-free, and one-pot design, the use of benign DMSO as solvent, and the high atom economy achieved.

The reaction demonstrates a broad substrate scope, affording the desired products in good to excellent yields with high selectivity. This strategy provides notable advantages in operational simplicity, energy efficiency, and environmental responsibility, representing a valuable contribution to the fields of green and heterocyclic synthesis.

The present domino multicomponent protocol, which combines pyrazole ring construction and subsequent C–H sulfenylation in a single operation, offers a promising platform for further development. Future studies will focus on domino multicomponent synthesis of other heterocyclic thioethers such as 4-oxazolyl thioethers.

## Conflicts of interest

There are no conflicts to declare.

## Supplementary Material

RA-015-D5RA07282D-s001

## Data Availability

All data supporting the findings of this study are available within the article and its supplementary information (SI). Supplementary information: the ^1^H and ^13^C NMR spectra of the synthesized compounds, together with their spectral data. See DOI: https://doi.org/10.1039/d5ra07282d.

## References

[cit1] Yang Z., Liu C., Lei J., Zhou Y., Gao X., Li Y. (2022). Chem. Commun..

[cit2] Cheng Z., Xu W., Tan X., Zhou Z., Zhou L., Liang Y., Yang Y. (2025). Org. Chem. Front..

[cit3] Wang X., Yan A., Xiao H., Xiao W., Xu L., Wang D. (2025). Org. Lett..

[cit4] Qin H., Wei G.-L., Zheng X.-W., Bao M.-H., Zhang Y.-W., Huang P. (2024). New J. Chem..

[cit5] Qin H., Wei G., Lou Y., Zheng X., Bao M., Zhang Y., Huang P. (2024). Org. Biomol. Chem..

[cit6] Reddy J. R., Kumari A. H. (2021). RSC Adv..

[cit7] Saroha M., Sindhu J., Kumar S., Bhasin K. K., Khurana J. M., Varma R. S., Tomar D. (2021). ChemistrySelect.

[cit8] Hosseinian A., Arshadi S., Sarhandi S., Monfared A., Vessally E. (2019). J. Sulfur Chem..

[cit9] Nowrouzi N., Abbasi M., Shaikhi Shahidzadeh E., Amaleh F. (2022). New J. Chem..

[cit10] Golzar N., Nowrouzi N., Abbasi M. (2018). Tetrahedron.

[cit11] Shaikhi Shahidzadeh E., Nowrouzi N., Abbasi M. (2019). Appl. Organomet. Chem..

[cit12] Arman A., Nowrouzi N., Abbasi M. (2024). Tetrahedron Lett..

[cit13] Abbasi M., Nowrouzi N., Sajedinia S. (2023). Mol. Diversity.

[cit14] Nowrouzi N., Abbasi M., Safari E., Arman A. (2024). Org. Biomol. Chem..

[cit15] Tan H., Zhang C., Deng Y., Zhang M., Cheng X., Wu J., Zheng D. (2023). Org. Lett..

[cit16] Li Y., Zhang W., Kweon J., Pan Y., Wang Q., Chang S., Wang Y. (2025). Nat. Commun..

[cit17] Qi Z., Wen S.-M., Wu Q., Jiang D.-F., Hao W.-J., Jiang B. (2023). J. Org. Chem..

[cit18] Qi Z., Wen S., Li H., Liu S., Jiang D. (2023). Org. Lett..

[cit19] Karrouchi K., Radi S., Ramli Y., Taoufik J., Mabkhot Y. N., Al-aizari F. A., Ansar M. (2018). Molecules.

[cit20] Becerra D., Castillo J. C. (2025). RSC Adv..

[cit21] Ameziane El Hassani I., Rouzi K., Assila H., Karrouchi K., Ansar M. (2023). Reactions.

[cit22] Yi F., Zhao W., Wang Z., Bi X. (2019). Org. Lett..

[cit23] Tang M., Wang Y., Wang H., Kong Y. (2016). Synthesis.

[cit24] Zheng Y., Long Y., Gong H., Xu J., Zhang C., Fu H., Zheng X., Chen H., Li R. (2022). Org. Lett..

[cit25] Panda N., Jena A. K. (2012). J. Org. Chem..

[cit26] Fan Z., Feng J., Hou Y., Rao M., Cheng J. (2020). Org. Lett..

[cit27] Lellek V., Chen C. Y., Yang W., Liu J., Ji X., Faessler R. (2018). Synlett.

[cit28] Zhang X., Kang J., Niu P., Wu J., Yu W., Chang J. (2014). J. Org. Chem..

[cit29] Ding Y., Zhang T., Chen Q. Y., Zhu C. (2016). Org. Lett..

[cit30] Akhmetova V. R. , Akhmadiev N. S. and Ibragimov A. G., Sulfur-Containing Pyrazoles, Pyrazolines and Indazoles, in N-Heterocycles, ed. K. L. Ameta, R. Kant, R. Penoni, A. Maspero and L. Scapinello, Springer, Singapore, 2022, 10.1007/978-981-19-0832-3_7

[cit31] Sun P., Yang D., Wei W., Sun X., Zhang W., Wang H., Wang Y., Wang H. (2017). Tetrahedron.

[cit32] Sun J., Qiu J. K., Zhu Y. L., Guo C., Hao W. J., Jiang B., Tu S. J. (2015). J. Org. Chem..

[cit33] Wei Y. T., Liu P., Liu Y., He J., Li X. Z., Li S. W., Zhao J. X. (2021). Org. Biomol. Chem..

[cit34] Yu X. Z., Shang Y. Z., Cheng Y. F., Tian J., Niu Y. L., Gao W. C. (2020). Org. Biomol. Chem..

[cit35] Annes S. B., Saritha R., Chandru K., Mandali P. K., Ramesh S. (2021). J. Org. Chem..

[cit36] Feng Y., He J., Wei Y., Xie J., Liu P. (2022). Eur. J. Org Chem..

[cit37] Liao Y.-S., Liang C.-F. (2018). Org. Biomol. Chem..

[cit38] Liu B.-B., Chu X.-Q., Liu H., Yin L., Wang S.-Y., Ji S.-J. (2017). J. Org. Chem..

[cit39] Sundaravelu N., Sangeetha S., Sekar G. (2021). Org. Biomol. Chem..

[cit40] Biswas K., Basu B. (2018). Curr. Organocatal..

[cit41] Boal A. K., Patsalis F. I. (2017). J.–Am. Water
Works Assoc..

[cit42] Hossain S. M. G., McLaughlan R. G. (2012). Water, Air, Soil Pollut..

[cit43] Chen T., Taylor-Edmonds L., Andrews S., Hofmann R. (2023). Front. Environ. Sci. Eng..

[cit44] Salehi M., Nowrouzi N. (2025). R. Soc. Open Sci..

[cit45] Lu X., Fu F., Gao R., Liu H., Wang H., Xiao J. (2019). New J. Chem..

[cit46] Shafie H., Abbasi M. (2024). Tetrahedron.

[cit47] Salehi M., Nowrouzi N., Abbasi M. (2025). R. Soc. Open Sci..

