# Supplementary material for: Sustainable multicomponent synthesis of C-4 sulfenylated pyrazoles via sodium thiosulfate-promoted tandem cyclocondensation and C–H sulfenylation
Source: RSC Adv. 2025 Oct 27;15(48):40737–44. doi: 10.1039/d5ra07282d (PMC12557309; doi:10.1039/d5ra07282d)
Supplement: RA-015-D5RA07282D-s001 [file RA-015-D5RA07282D-s001.pdf]

Supplementary Information (SI) for RSC Advances.
This journal is © The Royal Society of Chemistry 2025

Supporting information

For

Sustainable Multicomponent Synthesis of C-4 Sulfenylated Pyrazoles via Sodium Thiosulfate-Promoted Tandem Cyclocondensation and C-H Sulfenylation

Sanaz Abdollahi[a], Mohammad Abbasi[a,*] and Najmeh Nowrouzi[a]

[a]*Department of Chemistry, Faculty of Nano and Bio Science and Technology, Persian Gulf University, Bushehr 75169, Iran*

Characterization Data of the Products	S2
Copies of ^{1}H and ^{13}C NMR Spectra	S7

Characterization Data of the Products:

3,5-dimethyl-1-phenyl-4-(phenylthio)-1H-pyrazole (4a)[1]. red oil, ^{1}H-NMR (300 MHZ, Chloroform-*d*): δ (ppm) 7.52 – 7.44 (m, 4H, Ar), 7.40 – 7.35 (m, 2H, Ar), 7.34 (d, J = 1.3 Hz, 1H, Ar), 7.32 – 7.26 (m, 2H, Ar), 7.26 – 7.21 (m, 1H, Ar), 2.47 (s, 3H, CH_3), 2.22 (s, 3H, CH_3).^{13}C-NMR (75 MHZ, Chloroform-*d*): δ (ppm) 153.4, 146.5, 138.1, 136.9, 130.9, 130.4, 129.7, 128.9, 125.3, 124.9, 105.3, 12.1, 10.5.

4-((4-chlorophenyl)thio)-3,5-dimethyl-1-phenyl-1H-pyrazole (4b)[2]. red oil, ^{1}H-NMR (300 MHZ, Chloroform-*d*): δ (ppm) 7.52 – 7.44 (m, 4H, Ar), 7.42 – 7.33 (m, 5H, Ar), 2.47 (s, 3H, CH_3), 2.22 (s, 3H, CH_3). ^{13}C-NMR (75 MHZ, Chloroform-*d*): δ (ppm) 149.6, 142.4, 138.5, 134.4, 133.9, 129.4, 129.3, 129.1, 128.0, 124.6, 109.9, 13.4, 13.1.

4-((4-fluorophenyl)thio)-3,5-dimethyl-1-phenyl-1H-pyrazole (4c). red oil, ^{1}H-NMR (300 MHZ, Chloroform-*d*): δ (ppm) 7.52 – 7.43 (m, 6H, Ar), 7.41 – 7.33 (m, 1H, Ar), 7.19 – 7.12 (m, 2H, Ar), 2.47 (s, 3H, CH_3), 2.24 (s, 3H, CH_3). ^{13}C-NMR (75 MHZ, Chloroform-*d*): δ (ppm) 162.7, 160.7, 153.4, 146.5, 138.1, 132.4, 130.9, 130.4, 129.7, 129.0, 127.8, 125.3, 105.3, 12.1, 10.5.

Anal. Calcd for $C_{17}H_{15}FN_2S$: C, 68.43; H, 5.07; F, 6.37; N, 9.39; S, 10.74. Found: C, 68.25; H, 5.14; N, 9.28; S, 10.82.

3,5-dimethyl-1-phenyl-4-(p-tolylthio)-1H-pyrazole (4d)[1]. red oil, ^{1}H-NMR (300 MHZ, Chloroform-*d*): δ (ppm) 7.52 – 7.44 (m, 4H, Ar), 7.40 – 7.33 (m, 1H, Ar), 7.31 – 7.25 (m, 2H, Ar), 7.24 – 7.19 (m, 2H, Ar), 2.47 (s, 3H, CH_3), 2.36 (s, 3H, CH_3), 2.22 (s, 3H, CH_3). ^{13}C-NMR (75 MHZ, Chloroform-*d*): δ (ppm) 149.6, 142.4, 138.5, 137.4, 133.7, 129.9, 129.7, 129.4, 128.0, 124.6, 109.9, 21.1, 13.4, 13.1.

3,5-dimethyl-4-(naphthalen-1-ylthio)-1-phenyl-1H-pyrazole (4e)[1]. red oil, ^{1}H-NMR (300 MHZ, Chloroform-*d*): δ (ppm) 8.11 – 8.07 (m, 1H, Ar), 7.88 (dt, J = 7.8, 1.7 Hz, 1H, Ar), 7.79 (dt, J = 7.1, 1.6 Hz, 1H, Ar), 7.55 – 7.43 (m, 8H, Ar), 7.40 – 7.33 (m, 1H, Ar), 2.47 (s, 3H, CH_3), 2.22 (s, 3H, CH_3). ^{13}C-NMR (75 MHZ, Chloroform-*d*): δ (ppm) 149.3, 141.5, 138.5, 135.2, 133.8, 133.0, 129.4, 129.3, 129.2, 128.0, 127.0, 126.9, 126.8, 126.5, 126.0, 124.6, 109.6, 13.4, 13.1.

4-(benzylthio)-3,5-dimethyl-1-phenyl-1H-pyrazole (4f). red oil, ^{1}H-NMR (300 MHZ, Chloroform-*d*): δ (ppm) 7.52 – 7.44 (m, 4H, Ar), 7.42 – 7.28 (m, 5H, Ar), 7.27 – 7.22 (m, 1H, Ar), 4.11 (s, 2H, CH_2), 2.47 (s, 3H, CH_3), 2.21 (s, 3H, CH_3). ^{13}C-NMR (75 MHZ, Chloroform-*d*): δ (ppm) 148.6, 138.5, 138.1, 138.1, 129.4, 129.3, 128.7, 128.0, 127.7, 124.6, 123.8, 41.0, 13.3, 13.1.

Anal Calc. for $C_{18}H_{18}N_2S$: C, 73.43; H, 6.16; N, 9.52; S, 10.89. Found: C, 73.30; H, 6.09; N, 9.65; S, 10.96.

4-(hexylthio)-3,5-dimethyl-1-phenyl-1H-pyrazole (4g). red oil, ^{1}H-NMR (300 MHZ, Chloroform-*d*): δ (ppm) 7.52 – 7.44 (m, 4H, Ar), 7.42 – 7.33 (m, 1H, Ar), 3.02 (t, J = 6.2 Hz, 2H, CH_2), 2.47 (s, 3H, CH_3), 2.17 (s, 3H, CH_3), 1.62 (p, J = 6.2 Hz, 2H, CH_2), 1.45 – 1.37 (m, 2H, CH_2), 1.36 – 1.26 (m, 4H, CH_2), 0.93 – 0.85 (m, 3H, CH_3). ^{13}C-NMR (75 MHZ, Chloroform-*d*): δ (ppm) 148.9, 138.5, 137.2, 129.4, 128.0, 125.6, 124.6, 35.7, 31.5, 28.9, 28.3, 22.6, 14.1, 13.3, 13.1.

Anal Calc. for $C_{17}H_{24}N_2S$: C, 70.79; H, 8.39; N, 9.71; S, 11.11. Found: 70.68; H, 8.45; N, 9.66; S, 11.21.

1-(2-chlorophenyl)-3,5-dimethyl-4-(p-tolylthio)-1H-pyrazole (4h)[1]. red oil, ^{1}H-NMR (300 MHZ, Chloroform-*d*): δ (ppm) 7.59 (dd, J = 6.9, 1.6 Hz, 1H, Ar), 7.48 (dd, J = 6.9, 1.7 Hz, 1H, Ar), 7.44 – 7.35 (m, 2H, Ar), 7.31 – 7.25 (m, 2H, Ar), 7.24 – 7.19 (m, 2H, Ar), 2.51 (s, 3H, CH_3), 2.36 (s, 3H, CH_3), 2.24 (s, 3H,

CH_3). ^{13}C-NMR (75 MHZ, Chloroform-*d*): δ (ppm) 150.9, 143.2, 137.4, 137.0, 133.7, 131.2, 130.0, 129.9, 129.7, 129.2, 128.3, 126.2, 110.7, 21.1, 13.4, 12.9.

2-((1-(2-chlorophenyl)-3,5-dimethyl-1H-pyrazol-4-yl)thio)benzoic acid (4i). red oil, ^{1}H-NMR (300 MHZ, Chloroform-*d*): δ (ppm) 9.71 (s, 1H, OH), 8.00 (dd, *J = 7.6*, 1.7 Hz, 1H, Ar), 7.66 – 7.62 (m, 1H, Ar), 7.62 – 7.56 (m, 1H, Ar), 7.47-7.50 (m, 2H, Ar), 7.44 – 7.32 (m, 3H, Ar), 2.51 (s, 3H, CH_3), 2.23 (s, 3H, CH_3). ^{13}C-NMR (75 MHZ, Chloroform-*d*): δ (ppm) 168.4, 150.6, 142.1, 140.2, 137.0, 133.1, 132.1, 131.2, 130.0, 129.2, 128.3, 128.1, 126.2, 125.8, 110.5, 13.4, 12.9.

Anal Calc. for $C_{18}H_{15}ClN_2O_2S$: C, 60.25; H, 4.21; Cl, 9.88; N, 7.81; O, 8.92; S, 8.93. Found: C, 60.37; H, 4.29; N, 7.83; S, 8.80.

1-(2-chlorophenyl)-3,5-dimethyl-4-(naphthalen-1-ylthio)-1H-pyrazole (4j). red oil, ^{1}H-NMR (300 MHZ, Chloroform-*d*): δ (ppm) 8.13 – 8.07 (m, 1H, Ar), 7.88 (dt, *J* = 7.8, 1.6 Hz, 1H, Ar), 7.79 (dt, *J* = 7.2, 1.6 Hz, 1H, Ar), 7.59 (dd, *J* = 6.9, 1.5 Hz, 1H, Ar), 7.54 – 7.37 (m, 7H, Ar), 2.50 (s, 3H, CH_3), 2.23 (s, 3H, CH_3). ^{13}C-NMR (75 MHZ, Chloroform-*d*): δ (ppm) 150.8, 141.8, 137.0, 135.2, 133.8, 133.0, 131.2, 130.0, 129.3, 129.2, 129.2, 128.3, 127.0, 126.9, 126.8, 126.5, 126.2, 126.0, 110.7, 13.4, 12.9.

Anal Calc. for $C_{21}H_{17}ClN_2S$: C, 69.13; H, 4.70; Cl, 9.72; N, 7.68; S, 8.79. Found: C, 69.12; H, 4.81; N, 7.54; S, 8.90.

4-(benzylthio)-1-(2-chlorophenyl)-3,5-dimethyl-1H-pyrazole (4k). red oil, ^{1}H-NMR (300 MHZ, Chloroform-*d*): δ (ppm) 7.59 (dd, *J* = 6.9, 1.6 Hz, 1H, Ar), 7.48 (dd, *J* = 6.9, 1.7 Hz, 1H, Ar), 7.44 – 7.35 (m, 3H, Ar), 7.35 – 7.28 (m, 3H, Ar), 7.24 (m, 1H, Ar), 4.11 (s, 2H, CH_2), 2.49 (s, 3H, CH_3), 2.19 (s, 3H, CH_3). ^{13}C-NMR (75 MHZ, Chloroform-*d*): δ (ppm) 150.1, 138.7, 138.1, 137.1, 131.1, 130.0, 129.3, 129.2, 128.7, 128.3, 127.7, 126.2, 116.6, 41.0, 13.4, 12.8.

Anal Calc. for $C_{18}H_{17}ClN_2S$: C, 65.74; H, 5.21; Cl, 10.78; N, 8.52; S, 9.75. Found: C, 65.69; H, 5.11; N, 8.40; S, 9.90.

1-(2-chlorophenyl)-4-(hexylthio)-3,5-dimethyl-1H-pyrazole (4l). red oil, ^{1}H-NMR (300 MHZ, Chloroform-*d*): δ (ppm) 7.59 (dd, *J* = 6.9, 1.6 Hz, 1H, Ar), 7.48 (dd, *J* = 6.9, 1.7 Hz, 1H, Ar), 7.44 – 7.35 (m, 2H, Ar), 3.02 (t, *J* = 6.2 Hz, 2H, CH_2), 2.50 (s, 3H, CH_3), 2.17 (s, 3H, CH_3), 1.62 (p, *J* = 6.2 Hz, 2H, CH_2), 1.45 – 1.37 (m, 2H, CH_2), 1.37 – 1.26 (m, 4H, CH_2), 0.94 – 0.84 (m, 3H, CH_3). ^{13}C-NMR (75 MHZ, Chloroform-*d*): δ (ppm) 149.9, 138.3, 137.1, 131.1, 130.0, 129.2, 128.3, 126.2, 126.0, 35.7, 31.5, 28.9, 28.3, 22.6, 14.1, 13.4, 12.8.

Anal Calc. for $C_{17}H_{23}ClN_2S$: C, 63.24; H, 7.18; Cl, 10.98; N, 8.68; S, 9.93. Found: C, 63.35; H, 7.20; N, 8.55; S, 10.04.

1-(4-bromophenyl)-3,5-dimethyl-4-(phenylthio)-1H-pyrazole (4m). red oil, ^{1}H-NMR (300 MHZ, Chloroform-*d*): δ (ppm) 7.62 – 7.54 (m, 4H, Ar), 7.39 – 7.32 (m, 2H, Ar), 7.31 – 7.22 (m, 3H, Ar), 2.47 (s, 3H, CH_3), 2.24 (s, 3H, CH_3). ^{13}C-NMR (75 MHZ, Chloroform-*d*): δ (ppm) 149.6, 142.4, 137.2, 135.5, 132.2, 129.4, 129.2, 127.7, 125.3, 120.5, 109.9, 13.4, 13.1.

Anal Calc. for $C_{17}H_{15}BrN_2S$: C, 56.83; H, 4.21; Br, 22.24; N, 7.80; S, 8.92. Found: C, 56.83; H, 4.33; N, 7.90; S, 8.80.

1-(4-bromophenyl)-4-((4-chlorophenyl)thio)-3,5-dimethyl-1H-pyrazole (4n). red oil, ^{1}H-NMR (300 MHZ, Chloroform-*d*): δ (ppm) 7.62 – 7.53 (m, 4H, Ar), 7.42 – 7.34 (m, 4H, Ar), 2.47 (s, 3H, CH_3), 2.25 (s, 3H, CH_3).

^{13}C-NMR (75 MHZ, Chloroform-*d*): δ (ppm) 149.5, 142.3, 137.2, 134.4, 133.9, 132.2, 129.3, 129.1, 125.3, 120.5, 109.9, 13.4, 13.2.

Anal Calc. for $C_{17}H_{14}BrClN_2S$: C, 51.86; H, 3.58; Br, 20.29; Cl, 9.00; N, 7.12; S, 8.14. Found: C, 51.73; H, 3.63; N, 7.25; S, 8.00.

1-(4-bromophenyl)-4-((4-fluorophenyl)thio)-3,5-dimethyl-1H-pyrazole (4o). red oil, ^{1}H-NMR (300 MHZ, Chloroform-*d*): δ (ppm) 7.62 – 7.53 (m, 4H, Ar), 7.47 (m, 2H, Ar), 7.19 – 7.12 (m, 2H, Ar), 2.49 (s, 3H, CH_3), 2.22 (s, 3H, Ar). ^{13}C-NMR (75 MHZ, Chloroform-*d*): δ (ppm) 162.7, 160.7, 149.6, 142.4, 137.2, 132.3, 132.3, 132.2, 129.9, 129.9, 125.3, 120.5, 116.2, 116.0, 109.9, 13.4, 13.1.

Anal Calc. for $C_{17}H_{14}BrFN_2S$: C, 54.12; H, 3.74; Br, 21.18; F, 5.04; N, 7.43; S, 8.50. Found: C, 54.20; H, 3.60; N, 7.53; S, 8.38.

1-(4-bromophenyl)-3,5-dimethyl-4-(naphthalen-1-ylthio)-1H-pyrazole (4p). red oil, ^{1}H-NMR (300 MHZ, Chloroform-*d*): δ (ppm) 8.13 – 8.07 (m, 1H, Ar), 7.88 (dt, *J* = 7.8, 1.6 Hz, 1H, Ar), 7.79 (dt, *J* = 7.1, 1.6 Hz, 1H, Ar), 7.61 – 7.52 (m, 5H, Ar), 7.52 – 7.45 (m, 3H, Ar), 2.47 (s, 3H, CH_3), 2.20 (s, 3H, CH_3). ^{13}C-NMR (75 MHZ, Chloroform-*d*): δ (ppm) 149.3, 141.7, 137.2, 135.2, 133.8, 133.0, 132.2, 129.3, 129.2, 127.0, 126.9, 126.8, 126.5, 126.0, 125.3, 120.5, 109.6, 13.4, 13.1.

Anal Calc. for $C_{21}H_{17}BrN_2S$: C, 61.62; H, 4.19; Br, 19.52; N, 6.84; S, 7.83. Found: C, 61.56; H, 4.30; N, 6.70; S, 7.91.

3,5-dimethyl-4-(phenylthio)-1H-pyrazole (4q)[3]**.** red oil, ^{1}H-NMR (300 MHZ, Chloroform-*d*): δ (ppm) 8.93 (s, 1H, NH), 7.38 – 7.33 (m, 2H, Ar), 7.30 – 7.22 (m, 3H, Ar), 2.32 (s, 3H, CH_3), 2.25 (s, 3H, CH_3). ^{13}C-NMR (75 MHZ, Chloroform-*d*): δ (ppm) 148.6, 143.7, 136.0, 129.6, 129.2, 127.7, 107.2, 12.0, 11.4.

3,5-dimethyl-4-(p-tolylthio)-1H-pyrazole (4r)[4]**.** red oil, ^{1}H-NMR (300 MHZ, Chloroform-*d*): δ (ppm) 9.12 (s, 1H, NH), 7.31 – 7.25 (m, 2H, Ar), 7.24 – 7.19 (m, 2H, Ar), 2.36 (s, 3H, CH_3), 2.32 (s, 3H, CH_3), 2.25 (s, 3H, CH_3). ^{13}C-NMR (75 MHZ, Chloroform-*d*): δ (ppm) 148.6, 143.7, 137.4, 133.9, 129.9, 129.8, 107.2, 21.1, 12.0, 11.4.

4-((4-chlorophenyl)thio)-3,5-dimethyl-1H-pyrazole (4s)[5]**.** red oil, ^{1}H-NMR (300 MHZ, Chloroform-*d*): δ (ppm) 8.94 (s, 1H, NH), 7.42 – 7.34 (m, 4H, Ar), 2.32 (s, 3H, CH_3), 2.23 (s, 3H, CH_3). ^{13}C-NMR (75 MHZ, Chloroform-*d*): δ (ppm) 148.6, 143.7, 135.1, 133.9, 129.5, 129.1, 107.2, 12.0, 11.4.

4-(benzylthio)-3,5-dimethyl-1H-pyrazole (4t). red oil, ^{1}H-NMR (300 MHZ, Chloroform-*d*): δ (ppm) 9.07 (s, 1H, NH), 7.39 – 7.28 (m, 4H, Ar), 7.24 (m, 1H, Ar), 4.14 (s, 2H, CH_2), 2.29 (s, 3H, CH_3), 2.18 (s, 3H, CH_3). ^{13}C-NMR (75 MHZ, Chloroform-*d*): δ (ppm) 147.9, 141.4, 138.1, 129.3, 128.7, 127.7, 108.6, 41.3, 12.0, 11.4.

Anal Calc. for $C_{12}H_{14}N_2S$: C, 66.02; H, 6.46; N, 12.83; S, 14.69. Found: C, 66.08; H, 6.43; N, 12.93; S, 14.56.

1-(2-chlorophenyl)-3,5-diphenyl-4-(p-tolylthio)-1H-pyrazole (4u). red oil, ^{1}H-NMR (300 MHZ, Chloroform-*d*): δ (ppm) 7.74 – 7.70 (m, 1H, Ar), 7.64 – 7.57 (m, 4H, Ar), 7.55 – 7.37 (m, 10H, Ar), 7.31 – 7.27 (m, 2H, Ar), 7.23 – 7.21 (m, 1H, Ar), 2.36 (s, 3H, CH_3). ^{13}C-NMR (75 MHZ, Chloroform-*d*): δ (ppm) 152.1, 145.4, 137.6, 137.4, 133.3, 132.9, 132.3, 131.8, 131.1, 130.4, 129.9, 129.8, 129.7, 129.6, 129.2, 128.8, 128.5, 128.3, 128.0, 126.8, 125.4, 21.1.

Anal Calc. for $C_{28}H_{21}ClN_2S$: C, 74.24; H, 4.67; Cl, 7.83; N, 6.18; S, 7.08. Found: C, 74.14; H, 4.70; N, 6.07; S, 7.28.

2-((1-(4-bromophenyl)-3,5-diphenyl-1H-pyrazol-4-yl) thio) benzoic acid (4v). red oil, ^{1}H-NMR (300 MHZ, Chloroform-*d*): δ (ppm) 9.23 (s, 1H, OH), 8.00 (dd, *J* = 7.6, 1.8 Hz, 1H, Ar), 7.66 – 7.56 (m, 9H, Ar), 7.50 – 7.33 (m, 8H, Ar). ^{13}C-NMR (75 MHZ, Chloroform-*d*): δ (ppm) 168.5, 151.5, 145.2, 138.7, 138.4, 133.2, 133.1, 132.6, 132.1, 132.1, 129.7, 129.6, 128.8, 128.3, 128.1, 128.0, 126.8, 125.8, 124.7, 122.1, 120.5.

Anal Calc. for $C_{28}H_{19}BrN_2O_2S$: C, 63.76; H, 3.63; Br, 15.15; N, 5.31; O, 6.07; S, 6.08. Found: C, 63.88; H, 3.74; N, 5.25; S, 6.15.

4-(benzylthio)-3,5-diphenyl-1H-pyrazole (4w). red oil, ^{1}H-NMR (300 MHZ, Chloroform-*d*): δ (ppm) 9.26 (s, 1H, NH), 7.64 (dd, *J* = 8.6, 1.5 Hz, 2H, Ar), 7.59 – 7.53 (m, 2H, Ar), 7.49 (dd, *J* = 7.1, 1.3 Hz, 1H, Ar), 7.48 – 7.39 (m, 5H, Ar), 7.39 – 7.33 (m, 2H, Ar), 7.33 – 7.28 (m, 2H, Ar), 7.28 – 7.21 (m, 1H, Ar), 4.20 (s, 2H, CH_2). ^{13}C-NMR (75 MHZ, Chloroform-*d*): δ (ppm) 149.2, 141.8, 138.2, 132.3, 130.2, 129.7, 129.3, 129.3, 128.8, 128.7, 128.0, 127.7, 126.4, 125.3, 115.5, 41.2.

Anal Calc. for $C_{22}H_{18}N_2S$: C, 77.16; H, 5.30; N, 8.18; S, 9.36. Found: C, 77.05; H, 5.22; N, 8.30; S, 9.43.

4-(hexylthio)-1,3,5-triphenyl-1H-pyrazole (4x). red oil, ^{1}H-NMR (300 MHZ, Chloroform-*d*): δ (ppm) 7.64 – 7.37 (m, 15H, Ar), 3.06 (t, *J* = 6.2 Hz, 2H, CH_2), 1.61 (q, *J* = 6.2 Hz, 2H, CH_2), 1.45 – 1.37 (m, 2H, CH_2), 1.37 – 1.26 (m, 4H, CH_2), 0.92 – 0.86 (m, 3H, CH_3). ^{13}C-NMR (75 MHZ, Chloroform-*d*): δ (ppm) 152.2, 143.4, 139.4, 133.2, 131.7, 129.7, 129.6, 129.3, 128.8, 128.3, 128.0, 127.6, 126.7, 124.6, 124.5, 35.6, 31.5, 28.9, 28.3, 22.6, 14.1.

Anal Calc. for $C_{27}H_{28}N_2S$: C, 78.60; H, 6.84; N, 6.79; S, 7.77. Found: C, 78.65; H, 6.81; N, 6.90; S, 7.64.

4-(sec-butylthio)-1,3,5-triphenyl-1H-pyrazole (4y). red oil, ^{1}H-NMR (300 MHZ, Chloroform-*d*): δ (ppm) 7.66 – 7.34 (m, 15H, Ar), 3.33-3.39 (m, 1H, CH), 1.62 (qd, *J* = 7.1, 4.6 Hz, 2H, CH_2), 1.34 (d, *J* = 6.3 Hz, 3H, CH_3), 0.97 (t, *J* = 7.2 Hz, 3H, CH_3). ^{13}C-NMR (75 MHZ, Chloroform-*d*): δ (ppm) 152.9, 145.6, 139.5, 132.9, 131.5, 129.7, 129.6, 129.3, 128.8, 128.3, 128.0, 127.6, 126.7, 124.6, 122.0, 42.8, 29.9, 20.4, 11.4.

Anal Calc. for $C_{25}H_{24}N_2S$: C, 78.09; H, 6.29; N, 7.29; S, 8.34. Found: C, 77.97; H, 6.36; N, 7.20; S, 8.47.

4-((furan-2-ylmethyl)thio)-3,5-dimethyl-1-phenyl-1H-pyrazole (4z). red oil, ^{1}H-NMR (300 MHZ, Chloroform-*d*): δ (ppm) 7.52 – 7.42 (m, 5H, Ar), 7.42 – 7.33 (m, 1H, CH), 6.29-6.32 (m, 2H, 2×CH), 4.39 (s, 2H, CH_2), 2.47 (s, 3H, CH_3), 2.21 (s, 3H, CH_3). ^{13}C-NMR (75 MHZ, Chloroform-*d*): δ (ppm) 151.8, 149.1, 142.1, 138.5, 138.0, 129.4, 128.0, 124.6, 124.4, 110.9, 109.3, 33.1, 13.3, 13.1.

Anal Calc. for $C_{16}H_{16}N_2OS$: C, 67.58; H, 5.67; N, 9.85; O, 5.63; S, 11.27. Found: C, 67.71; H, 5.71; N, 9.80; S, 11.20.

1,2-bis ((3,5-dimethyl-1-phenyl-1H-pyrazol-4-yl) thio) ethane (4aa). red oil, ^{1}H-NMR (300 MHZ, Chloroform-*d*): δ (ppm) 7.52 – 7.44 (m, 4H, Ar), 7.42 – 7.33 (m, 1H, Ar), 3.34 (s, 2H, CH_2), 2.47 (s, 3H, CH_3), 2.22 (s, 3H, CH_3). ^{13}C-NMR (75 MHZ, Chloroform-*d*): δ (ppm) 148.8, 138.5, 137.1, 129.4, 128.0, 125.5, 124.6, 33.5, 13.3, 13.1.

Anal Calc. for $C_{24}H_{26}N_4S_2$: C, 66.33; H, 6.03; N, 12.89; S, 14.75. Found: C, 66.41; H, 6.12; N, 12.77; S, 14.70.

5-methyl-1-phenyl-4-(phenylthio)-1H-pyrazol-3-ol (6a)[1]**.** brown oil, ^{1}H-NMR (300 MHZ, Chloroform-*d*): δ (ppm) 9.27 (s, 1H, OH), 7.52 – 7.45 (m, 4H, Ar), 7.41 – 7.28 (m, 5H, Ar), 7.28 – 7.21 (m, 1H, Ar), 2.52 (s, 3H, CH_3). ^{13}C-NMR (75 MHZ, Chloroform-*d*): δ (ppm) 163.7, 144.8, 134.9, 133.4, 129.4, 129.4, 129.2, 128.0, 127.7, 125.1, 100.4, 13.0.

5-methyl-1-phenyl-4-(p-tolylthio)-1H-pyrazol-3-ol (6b)[1]. brown oil, ^{1}H-NMR (300 MHZ, Chloroform-*d*): δ (ppm) 9.41 (s, 1H, OH), 7.52 – 7.45 (m, 4H, Ar), 7.41 – 7.32 (m, 1H, Ar), 7.29 – 7.24 (m, 2H, Ar), 7.21 (dd, *J* = 7.6, 0.9 Hz, 2H, Ar), 2.52 (s, 3H, CH_3), 2.36 (s, 3H, CH_3). ^{13}C-NMR (75 MHZ, Chloroform-*d*): δ (ppm) 163.7, 144.8, 137.4, 133.5, 133.4, 130.0, 129.9, 129.4, 128.0, 126.1, 100.4, 21.1, 13.0.

4-((4-chlorophenyl) thio)-5-methyl-1-phenyl-1H-pyrazol-3-ol (6c)[1]. brown oil, ^{1}H-NMR (300 MHZ, Chloroform-*d*): δ (ppm) 8.89 (s, 1H, OH), 7.52 – 7.45 (m, 4H, Ar), 7.41 – 7.33 (m, 5H, Ar), 2.55 (s, 3H, CH_3). ^{13}C-NMR (75 MHZ, Chloroform-*d*): δ (ppm) 163.7, 144.8, 133.9, 133.7, 133.4, 129.5, 129.4, 129.1, 128.0, 125.1, 100.4, 13.0.

1-(2-chlorophenyl)-5-methyl-4-(p-tolylthio)-1H-pyrazol-3-ol (6d)[1]. brown oil, ^{1}H-NMR (300 MHZ, Chloroform-*d*): δ (ppm) 9.20 (s, 1H, OH), 7.61 (dd, *J* = 7.0, 1.6 Hz, 1H, Ar), 7.48 (dd, *J* = 6.8, 1.7 Hz, 1H, Ar), 7.44 – 7.34 (m, 2H, Ar), 7.29 – 7.19 (m, 4H, Ar), 2.54 (s, 3H, CH_3), 2.36 (s, 3H, CH_3). ^{13}C-NMR (75 MHZ, Chloroform-*d*): δ (ppm) 165.6, 145.1, 137.4, 133.5, 132.3, 131.3, 130.0, 130.0, 129.9, 129.2, 128.3, 126.6, 101.0, 21.1, 12.8.

1-(2-chlorophenyl)-5-methyl-4-(naphthalen-1-ylthio)-1H-pyrazol-3-ol (6e). brown oil, ^{1}H-NMR (300 MHZ, Chloroform-*d*): δ (ppm) 9.55 (s, 1H, OH), 8.14 – 8.09 (m, 1H, Ar), 7.88 (dt, *J* = 7.6, 1.6 Hz, 1H, Ar), 7.79 (dt, *J* = 7.2, 1.5 Hz, 1H, Ar), 7.61 (dd, *J* = 7.0, 1.9 Hz, 1H, Ar), 7.56 – 7.34 (m, 7H, Ar), 2.54 (s, 3H, CH_3). ^{13}C-NMR (75 MHZ, Chloroform-*d*): δ (ppm) 165.9, 144.4, 134.8, 133.9, 133.3, 132.3, 131.3, 130.0, 129.6, 129.3, 129.2, 128.3, 126.9, 126.8, 126.6, 126.5, 126.1, 100.8, 12.8.

Anal Calc. for $C_{20}H_{15}ClN_2OS$: C, 65.48; H, 4.12; Cl, 9.66; N, 7.64; O, 4.36; S, 8.74. Found: C, 65.60; H, 4.22; N, 7.59; S, 8.70.

1-(4-bromophenyl)-5-methyl-4-(phenylthio)-1H-pyrazol-3-ol (6f)[6]. brown oil, ^{1}H-NMR (300 MHZ, Chloroform-*d*): δ (ppm) 9.38 (s, 1H, OH), 7.62 – 7.52 (m, 4H, Ar), 7.39 – 7.28 (m, 4H, Ar), 7.28 – 7.21 (m, 1H, Ar), 2.54 (s, 3H, CH_3). ^{13}C-NMR (75 MHZ, Chloroform-*d*): δ (ppm) 163.7, 144.9, 134.9, 132.2, 132.2, 129.4, 129.2, 127.7, 125.7, 120.5, 100.4, 13.0.

1-(4-bromophenyl)-5-methyl-4-(p-tolylthio)-1H-pyrazol-3-ol (6g). brown oil, ^{1}H-NMR (300 MHZ, Chloroform-*d*): δ (ppm) 9.20 (s, 1H, OH), 7.62 – 7.52 (m, 4H, Ar), 7.29 – 7.24 (m, 2H, Ar), 7.21 (dd, *J* = 7.6, 0.9 Hz, 2H, Ar), 2.52 (s, 3H, CH_3), 2.36 (s, 3H, CH_3). ^{13}C-NMR (75 MHZ, Chloroform-*d*): δ (ppm) 163.7, 144.9, 137.4, 133.5, 132.2, 132.2, 130.0, 129.9, 125.7, 120.5, 100.4, 21.1, 13.0.

Anal Calc. for $C_{17}H_{15}BrN_2OS$: C, 54.41; H, 4.03; Br, 21.29; N, 7.46; O, 4.26; S, 8.54. Found: C, 54.29; H, 4.13; N, 7.40; S, 8.50.

5-methyl-4-(p-tolylthio)-1H-pyrazol-3-ol (6h)[7]. brown oil, ^{1}H-NMR (300 MHZ, Chloroform-*d*): δ (ppm) 9.57 (s, 1H, NH), 9.39 (s, 1H, OH), 7.29 – 7.19 (m, 4H, Ar), 2.36 (s, 6H, 2×CH_3). ^{13}C-NMR (75 MHZ, Chloroform-*d*): δ (ppm) 161.0, 145.5, 137.4, 134.2, 130.0, 129.9, 97.0, 21.1, 11.4.

1-(4-bromophenyl)-4-(naphthalen-1-ylthio)-1H-pyrazole-3,5-diol (6i)[8]. brown oil, ^{1}H-NMR (300 MHZ, Chloroform-*d*): δ (ppm) 9.81 (s, 1H, OH), 9.77 (s, 1H, OH), 8.15 – 8.10 (m, 1H, Ar), 7.88 (dt, *J* = 7.7, 1.7 Hz, 1H, Ar), 7.79 (dt, *J* = 7.4, 1.4 Hz, 1H, Ar), 7.68 – 7.63 (m, 2H, Ar), 7.63 – 7.57 (m, 2H, Ar), 7.56 – 7.44 (m, 3H, Ar), 7.42 (dd, *J* = 6.9, 1.4 Hz, 1H, Ar). ^{13}C-NMR (75 MHZ, Chloroform-*d*): δ (ppm) 160.8, 160.7, 133.9, 133.5, 132.7, 132.6, 132.1, 129.7, 129.3, 127.0, 126.9, 126.8, 126.5, 126.1, 123.2, 120.6, 82.1.

4-(benzylthio)-1H-pyrazole-3,5-diol (6j). brown oil, ^{1}H-NMR (300 MHZ, Chloroform-*d*): δ (ppm) 9.87 (s, 1H, NH), 9.52 (s, 1H, OH), 9.31 (s, 1H, OH), 7.39 – 7.28 (m, 4H, Ar), 7.28 – 7.21 (m, 1H, Ar), 4.11 (s, 2H, CH_2). ^{13}C-NMR (75 MHZ, Chloroform-*d*): δ (ppm) 158.7, 155.6, 138.1, 129.3, 128.7, 127.7, 82.3, 41.3.

Anal Calc. for $C_{10}H_{10}N_2O_2S$: C, 54.04; H, 4.54; N, 12.60; O, 14.40; S, 14.42. Found: C, 54.15; H, 4.54; N, 12.73; S, 14.30.

4-(benzylthio)-1-(2-chlorophenyl)-3-methyl-1H-pyrazol-5-ol (6k). brown oil, ^{1}H-NMR (300 MHZ, Chloroform-*d*): δ (ppm) 9.84 (s, 1H, OH), 7.56 – 7.48 (m, 1H, Ar), 7.46 – 7.28 (m, 7H, Ar), 7.24 (m, 1H, Ar), 4.21 (s, 2H, CH_2), 2.32 (s, 3H, CH_3). ^{13}C-NMR (75 MHZ, Chloroform-*d*): δ (ppm) 158.8, 153.6, 138.2, 137.4, 130.4, 129.3, 129.2, 129.0, 128.7, 128.5, 127.7, 123.5, 95.2, 40.6, 13.4.

Anal Calc. for $C_{17}H_{15}ClN_2OS$: C, 61.72; H, 4.57; Cl, 10.72; N, 8.47; O, 4.84; S, 9.69. Found: C, 61.60; H, 4.63; N, 8.40; S, 9.77.

4-(hexylthio)-3-methyl-1-phenyl-1H-pyrazol-5-ol (6l). brown oil, ^{1}H-NMR (300 MHZ, Chloroform-*d*): δ (ppm) 9.63 (s, 1H, OH), 7.81 – 7.75 (m, 2H, Ar), 7.56 – 7.48 (m, 2H, Ar), 7.37 (m, 1H, Ar), 3.04 (t, *J* = 6.2 Hz, 2H, CH_2), 2.30 (s, 3H, CH_3), 1.62 (p, *J* = 6.2 Hz, 2H, CH_2), 1.45 – 1.37 (m, 2H, CH_2), 1.37 – 1.26 (m, 4H, 2×CH_2), 0.92 – 0.86 (m, 3H, CH_3). ^{13}C-NMR (75 MHZ, Chloroform-*d*): δ (ppm) 159.2, 152.2, 139.5, 129.2, 127.0, 122.0, 95.9, 35.7, 31.5, 28.9, 28.3, 22.6, 14.1, 13.3.

Anal Calc. for $C_{16}H_{22}N_2OS$: C, 66.17; H, 7.64; N, 9.65; O, 5.51; S, 11.04. Found: C, 66.28; H, 7.70; N, 9.69; S, 10.94.

References:

1. P. Sun, D. Yang, W. Wei, X. Sun, W. Zhang, H. Zhang, H. Wang, Tetrahedron, 2017, 73, 2022–2029.
2. J. Sun, J.-K. Qiu, Y.-L. Zhu, C. Guo, W.-J. Hao, B. Jiang, S.-J. Tu, J. Org. Chem., 2015, 80, 8217–8224.
3. S. Paul, S. Das, T. Choudhuri, P. Sikdar, A. K. Bagdi, J. Org. Chem., 2023, 88, 4187–4198.
4. K. Tanimoto, R. Ohkado, H. Iida, J. Org. Chem., 2019, 84, 14980–14986.
5. A. Saeed, P. A. Channar, J. Heterocycl. Chem., 2017, 54, 780–783.
6. Y. Siddaraju, K. R. Prabhu, Org. Biomol. Chem., 2017, 15, 5191–5196.
7. M. Messaad, I. Dhouib, M. Abdelhedi, B. Khemakhem, J. Mol. Struct., 2022, 1263, 133105.
8. Y. Yuan, L.-S. Li, L. Zhang, F. Wang, L. Jiang, L. Zuo, Q. Wang, J.-G. Hu, A. Lei, Chem. Commun., 2021, 57, 2768–2771.

^{1}H NMR and ^{13}C NMR spectra of the synthesized compounds

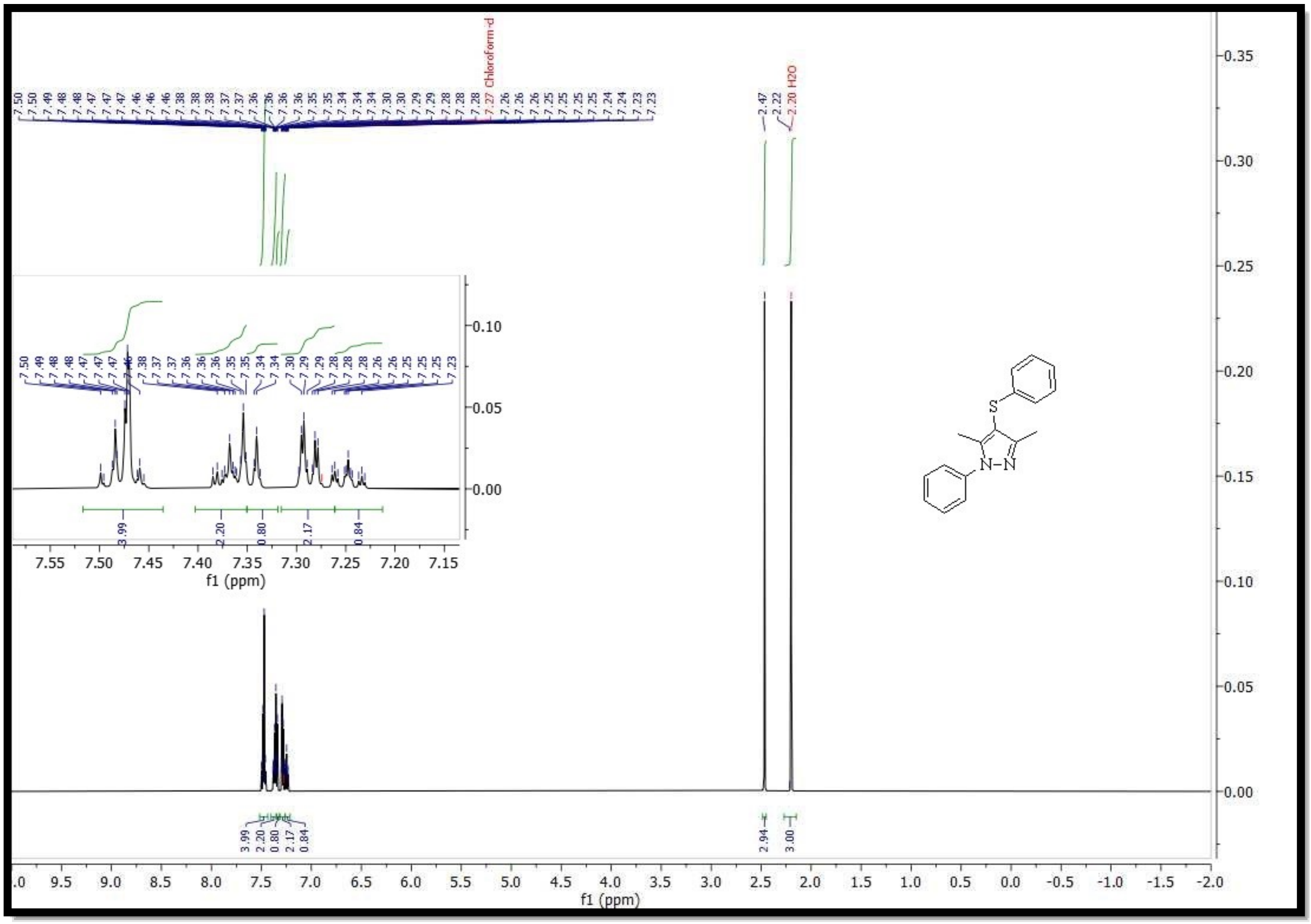

Figure 1: ^{1}H-NMR spectrum of compound **4a**

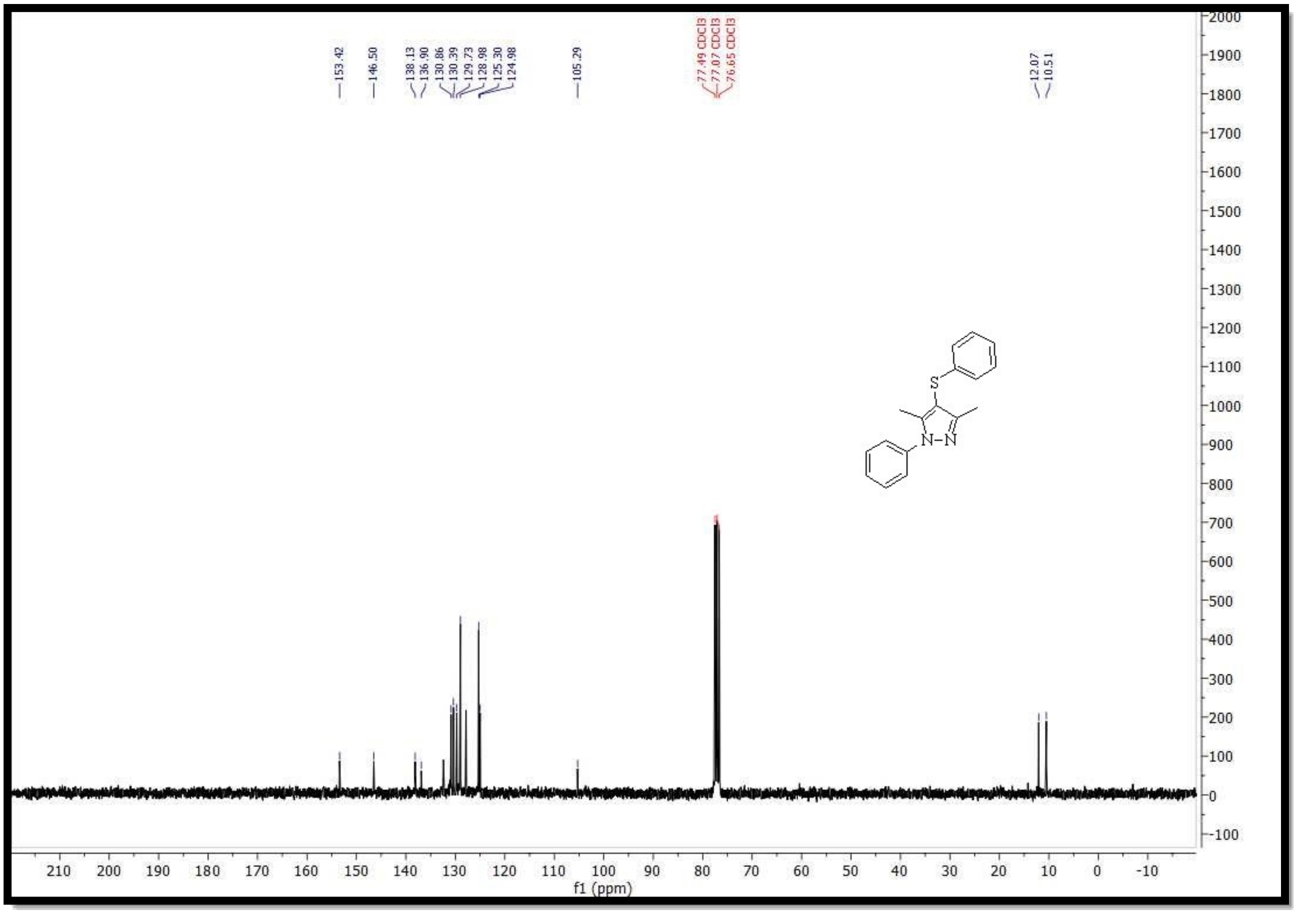

Figure 2: ^{13}C-NMR spectrum of compound **4a**

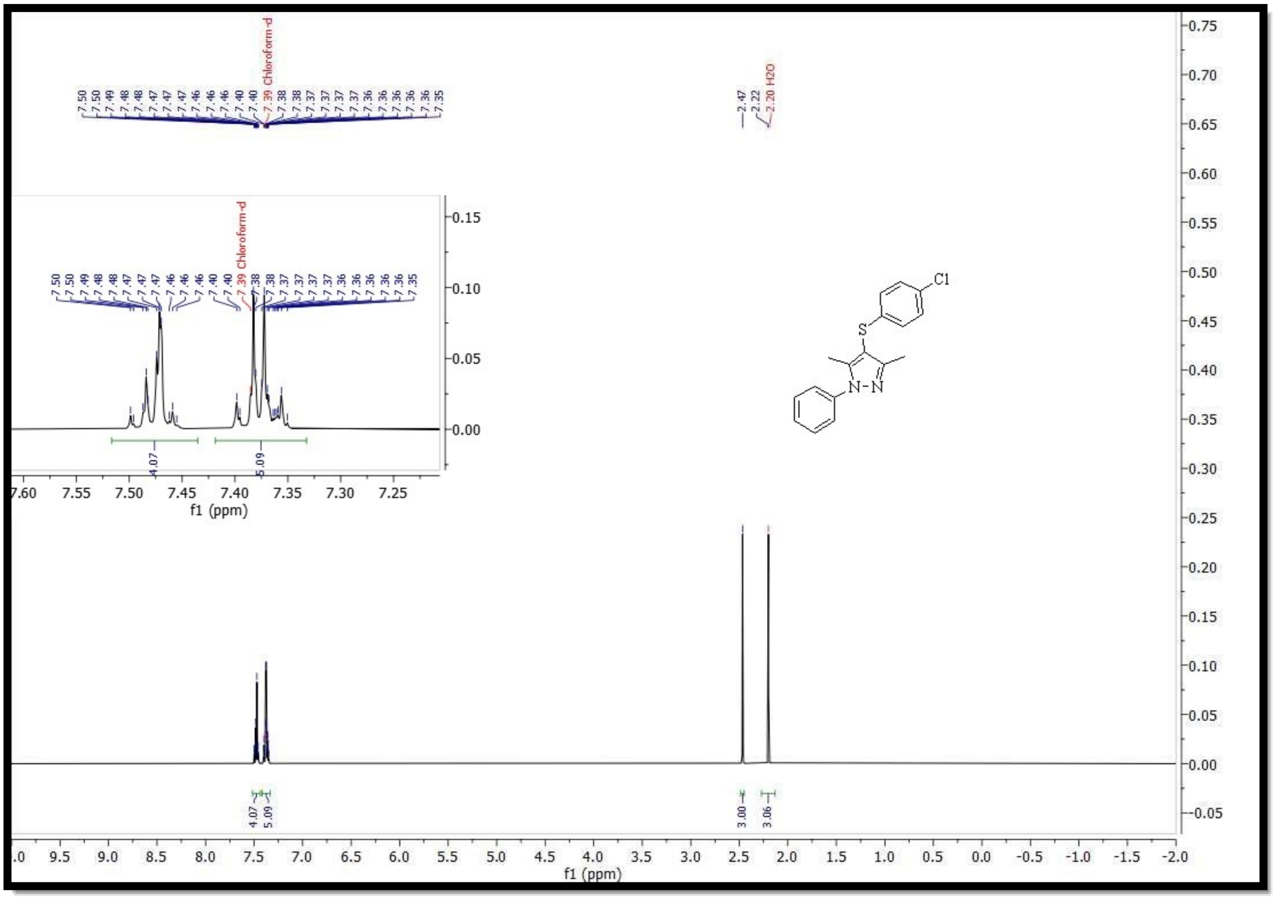

Figure 3: ^{1}H-NMR spectrum of compound **4b**

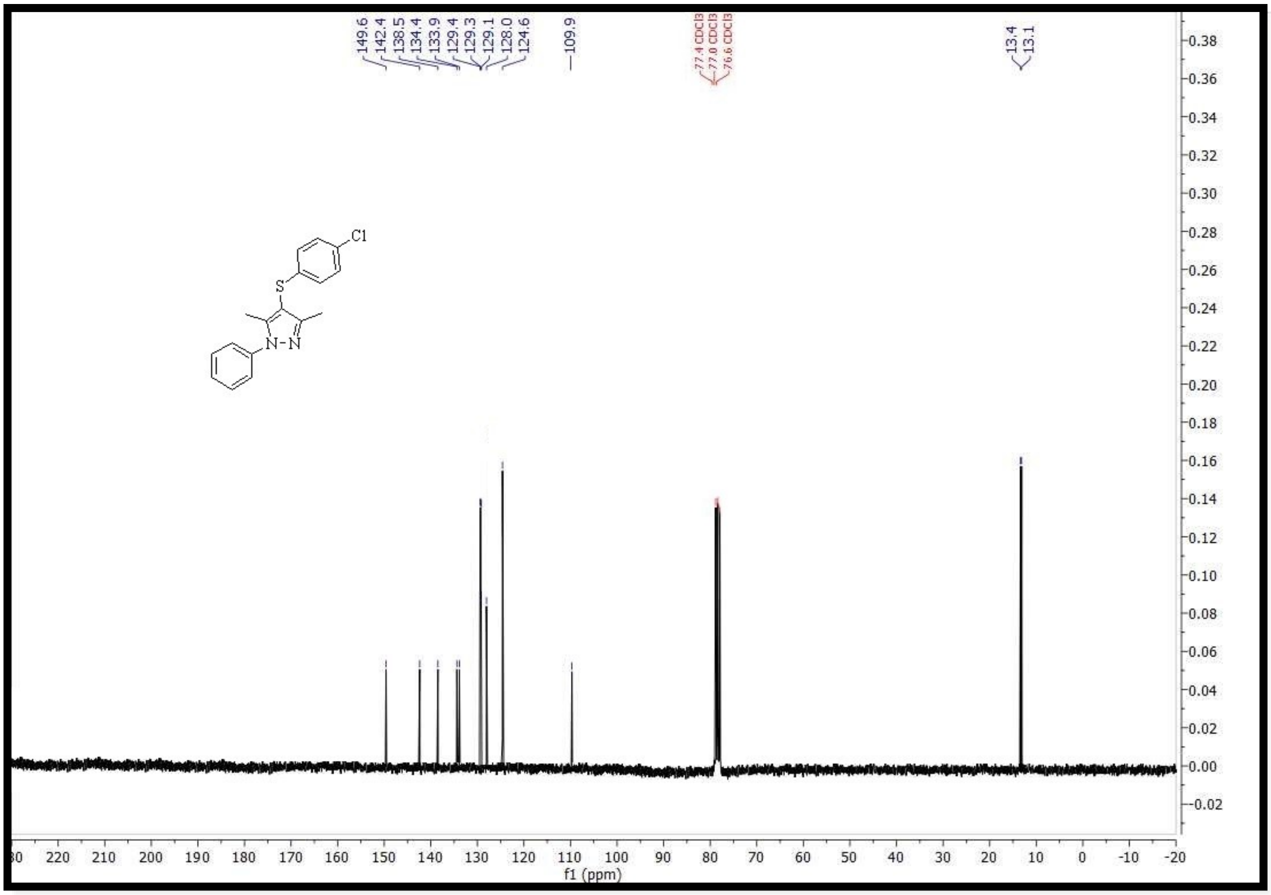

Figure 4: ^{13}C-NMR spectrum of compound **4b**

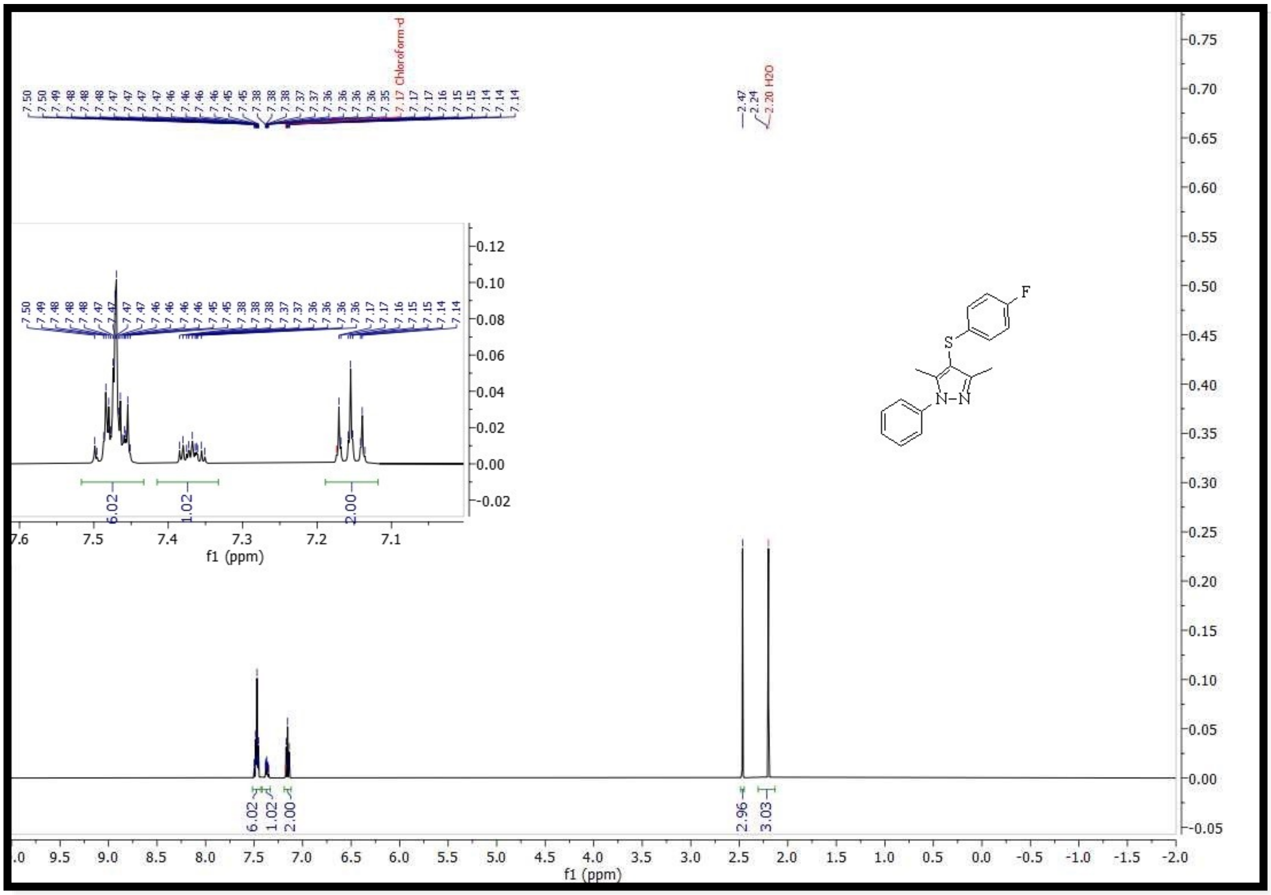

Figure 5: ^{1}H-NMR spectrum of compound **4c**

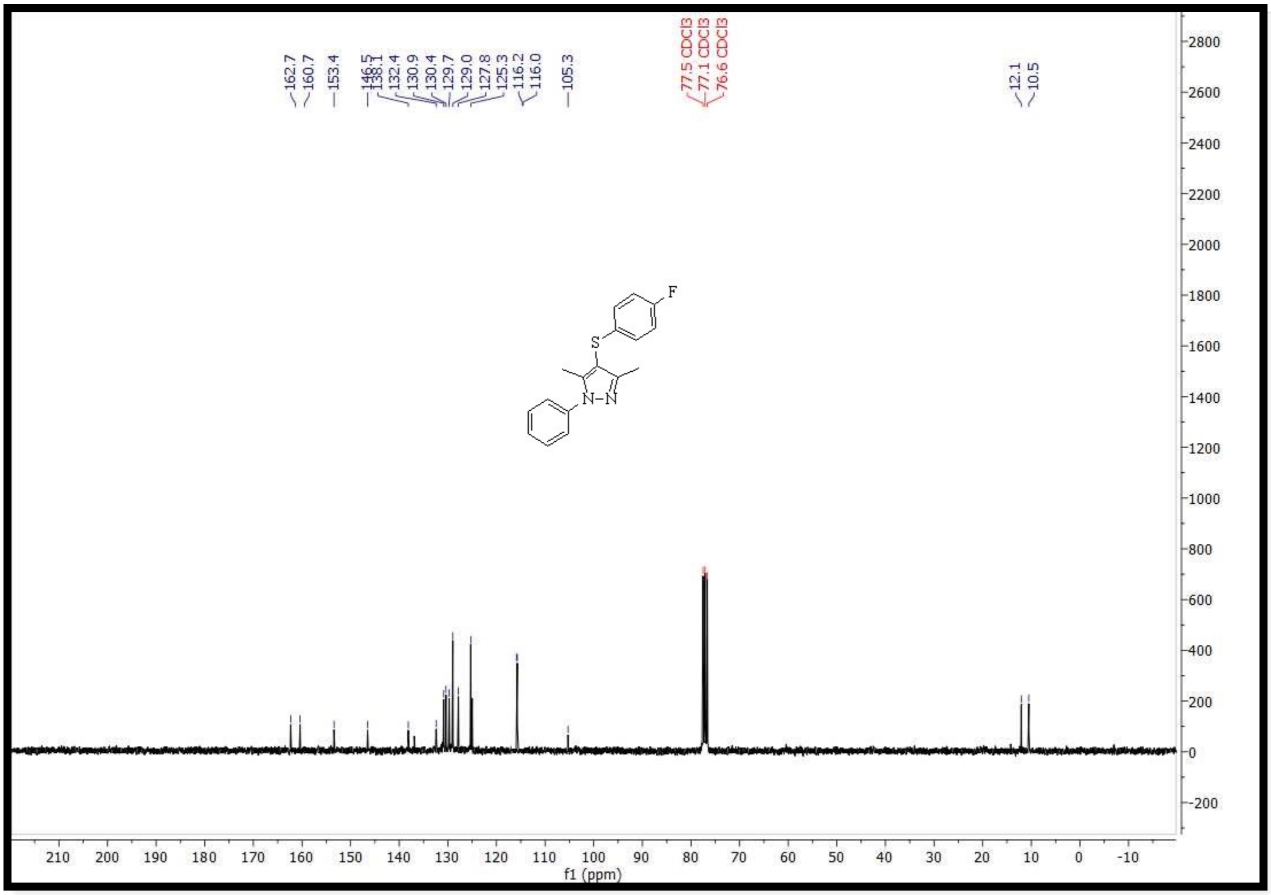

Figure 6: ^{13}C-NMR spectrum of compound **4c**

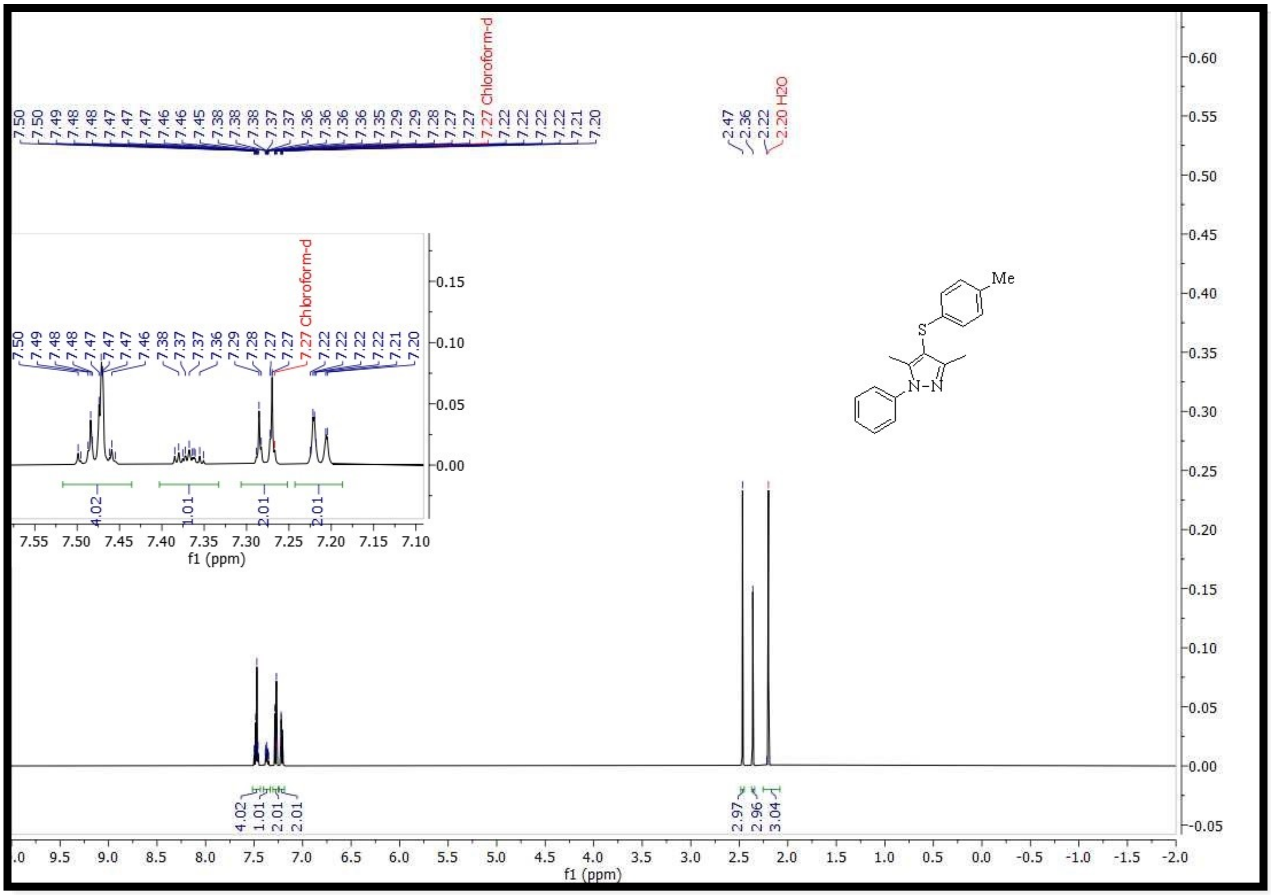

Figure 7: ^{1}H-NMR spectrum of compound **4d**

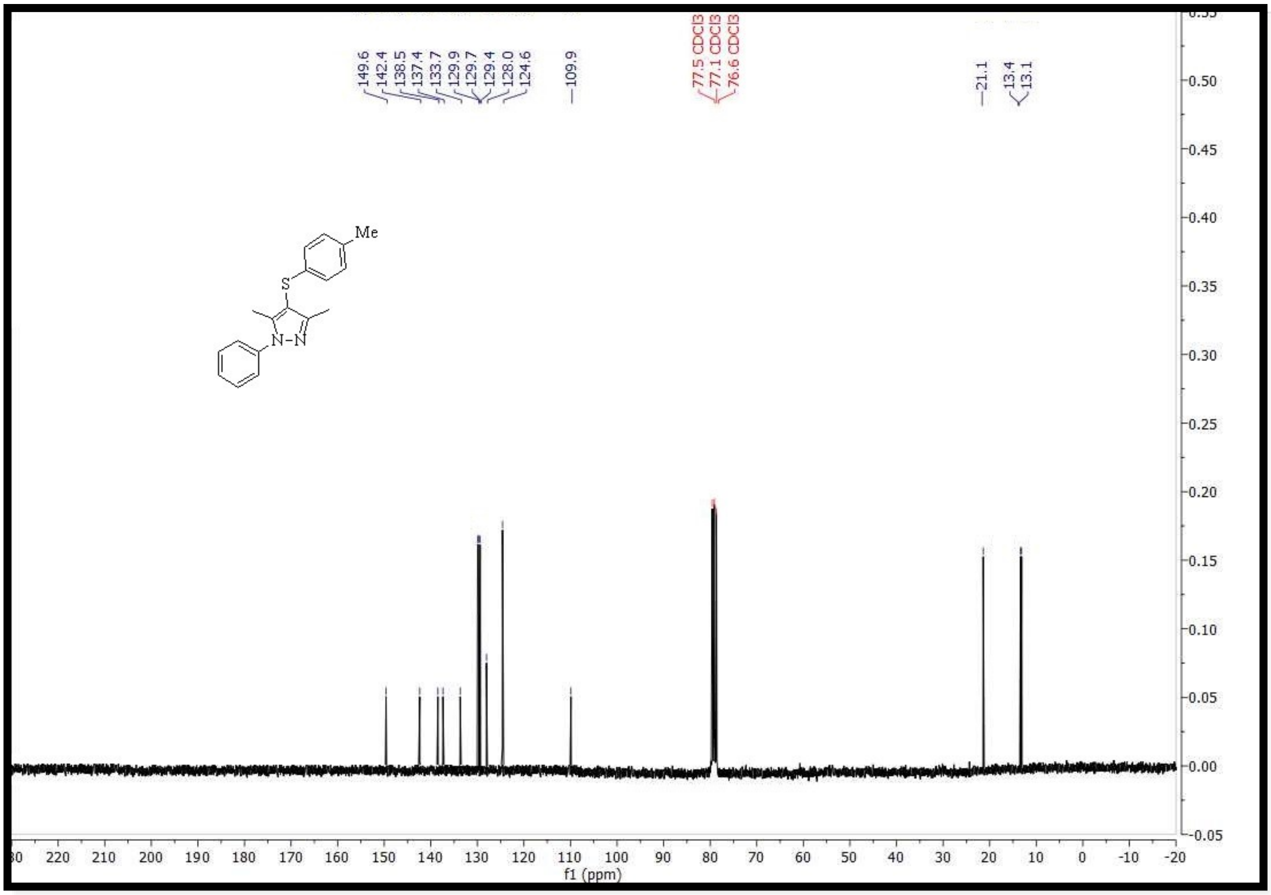

Figure 8: ^{13}C-NMR spectrum of compound **4d**

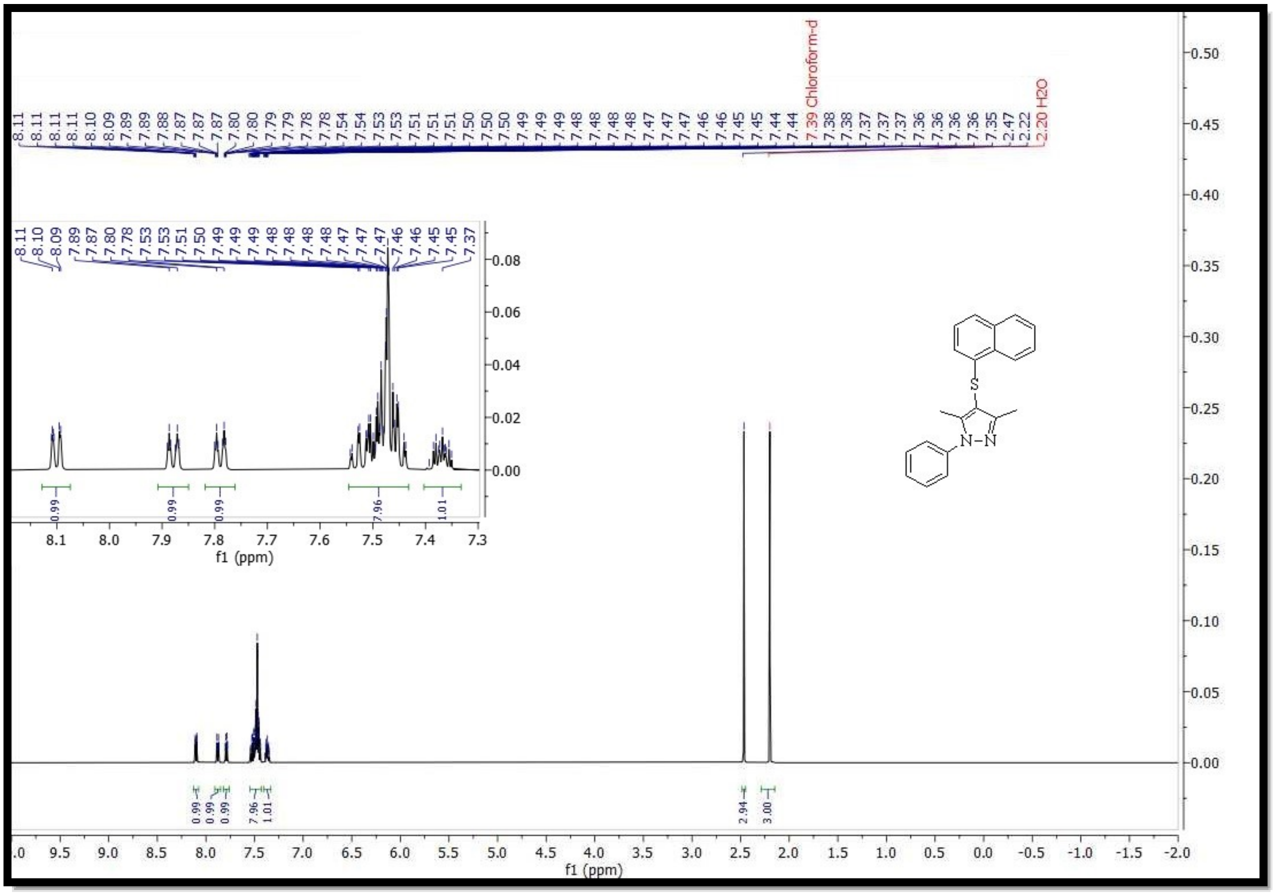

Figure 9: ^{1}H-NMR spectrum of compound **4e**

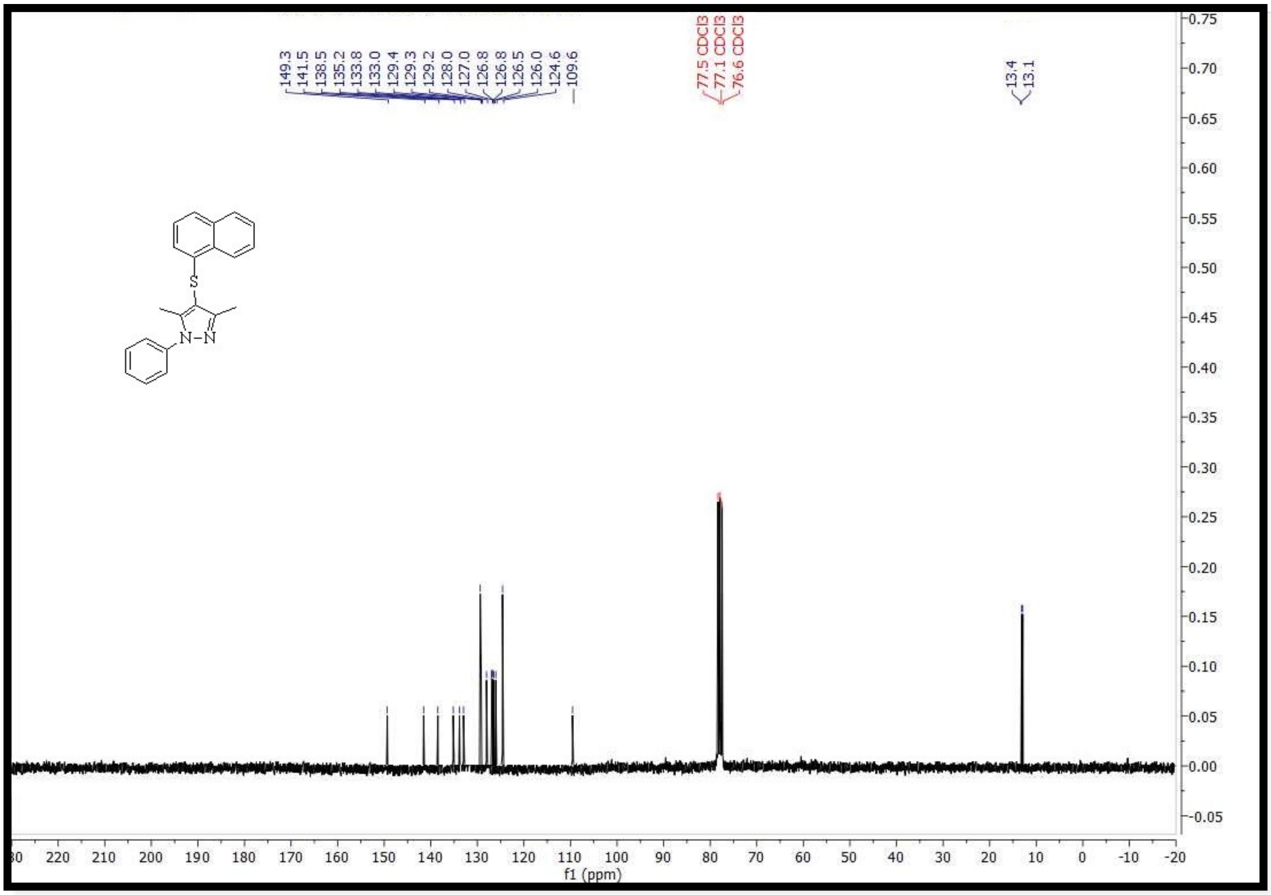

Figure 10: ^{13}C-NMR spectrum of compound **4e**

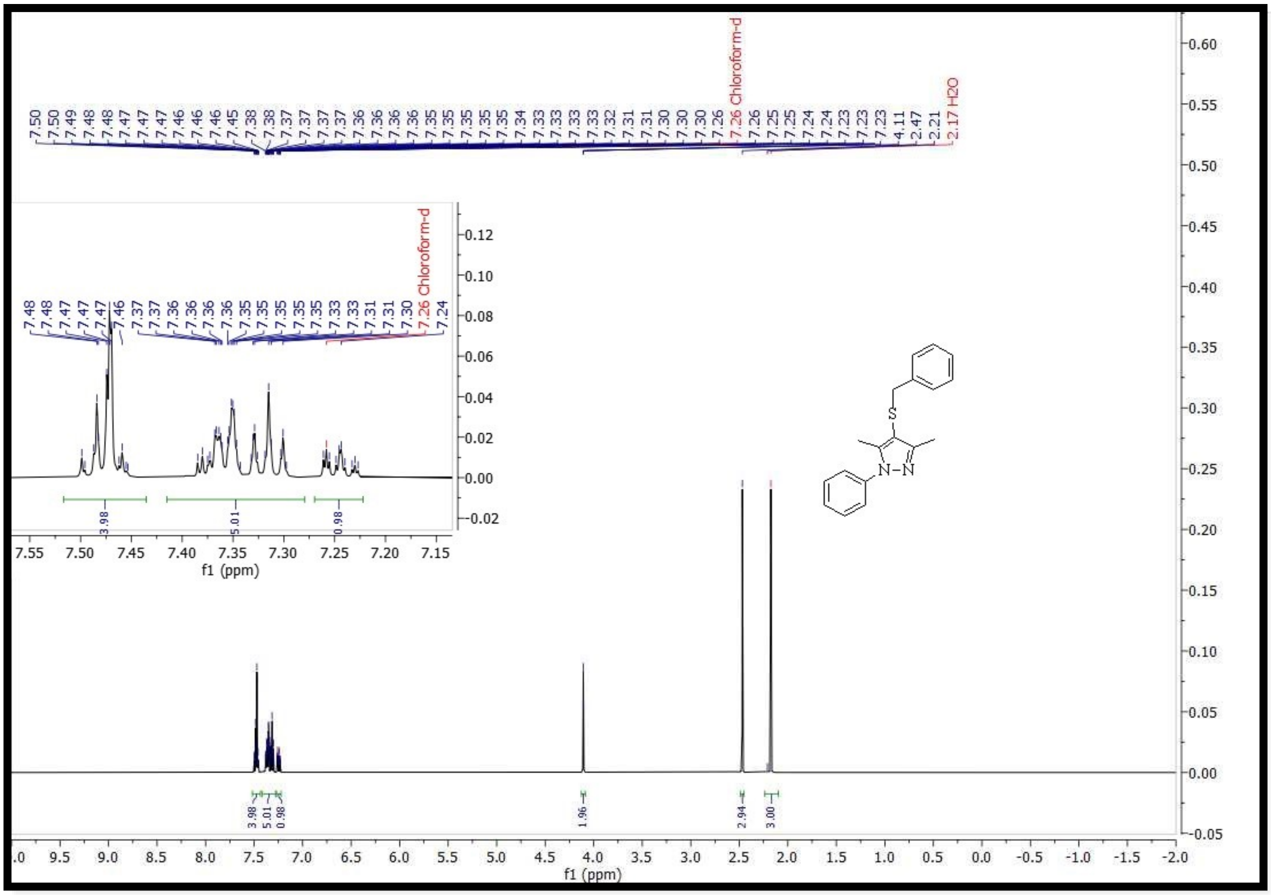

Figure 11: ^{1}H-NMR spectrum of compound **4f**

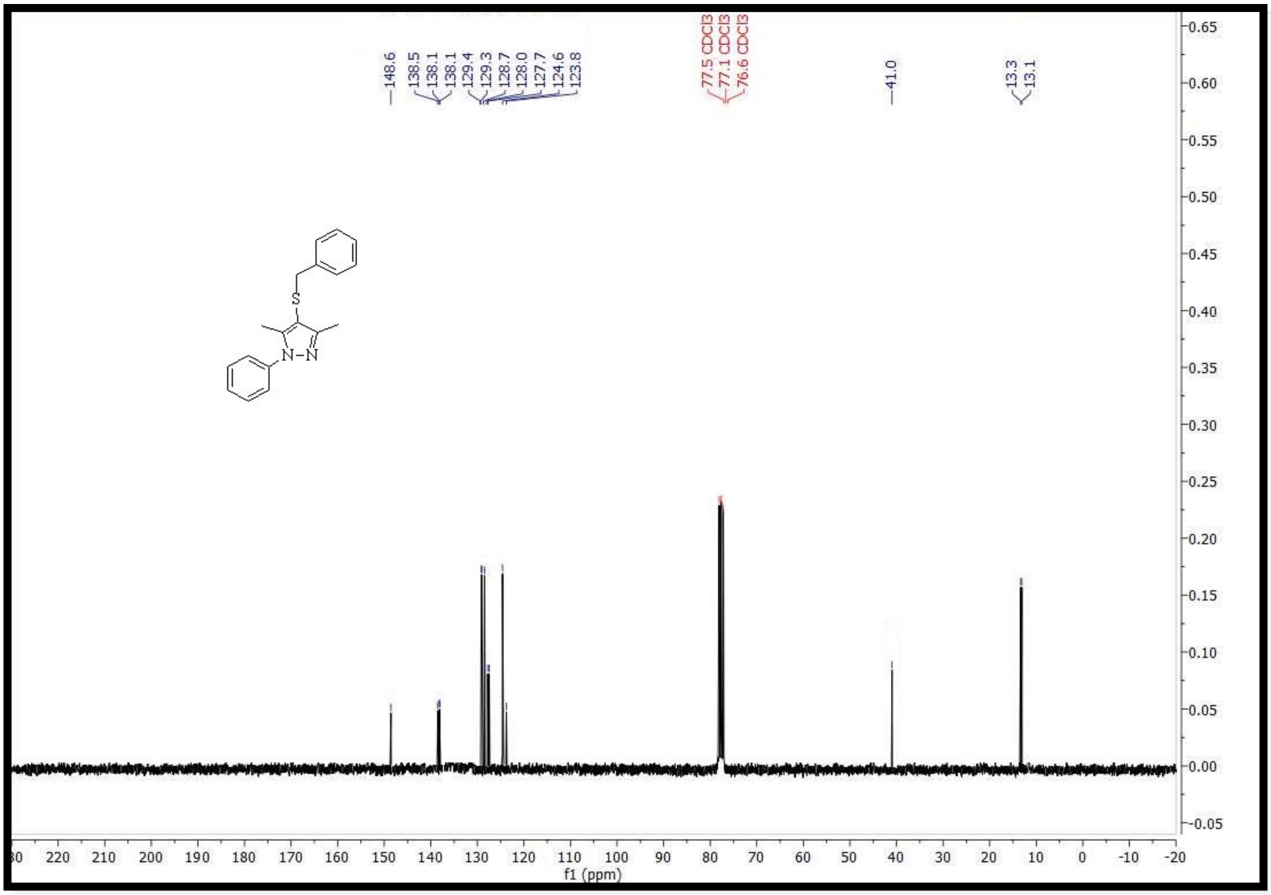

Figure 12: ^{13}C-NMR spectrum of compound **4f**

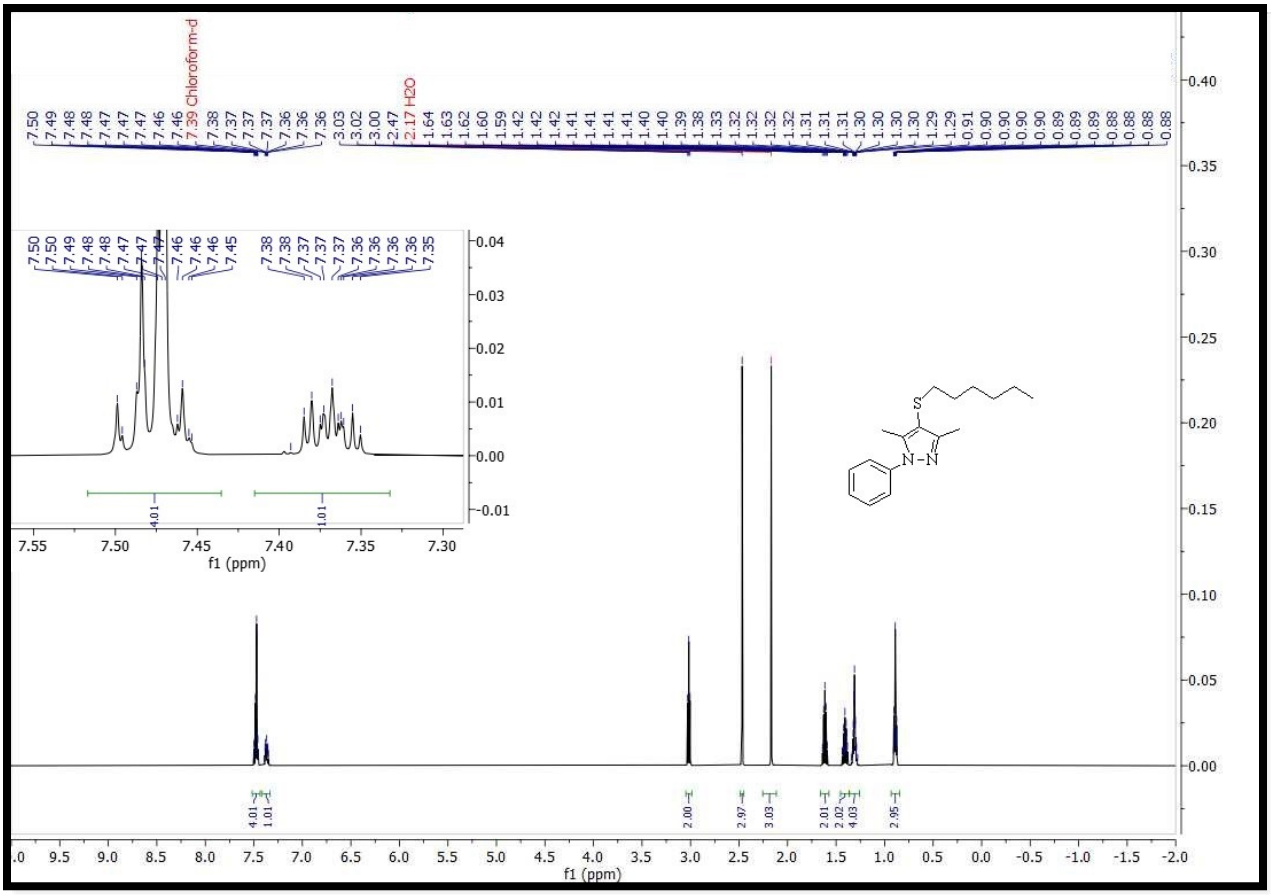

Figure 13: ^{1}H-NMR spectrum of compound **4g**

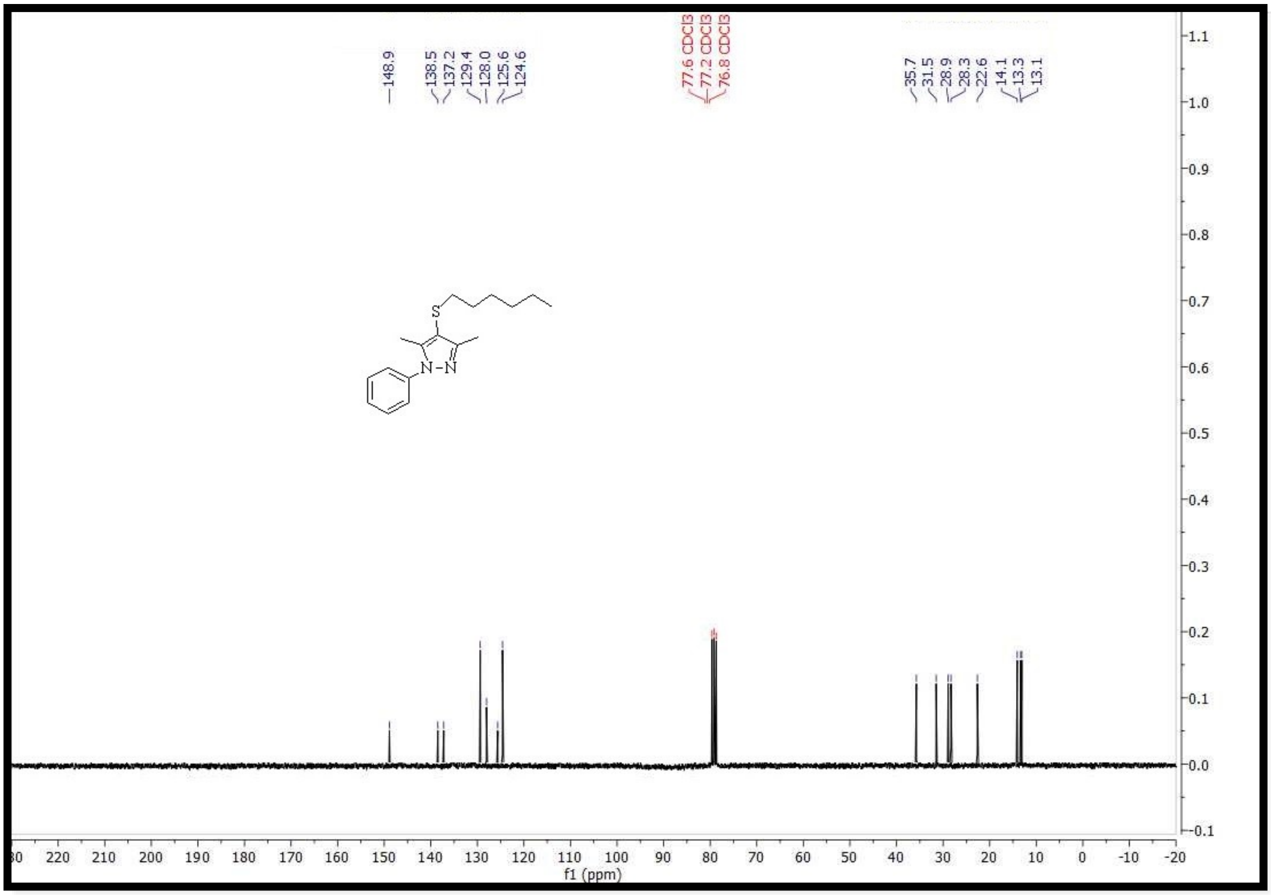

Figure 14: ^{13}C-NMR spectrum of compound **4g**

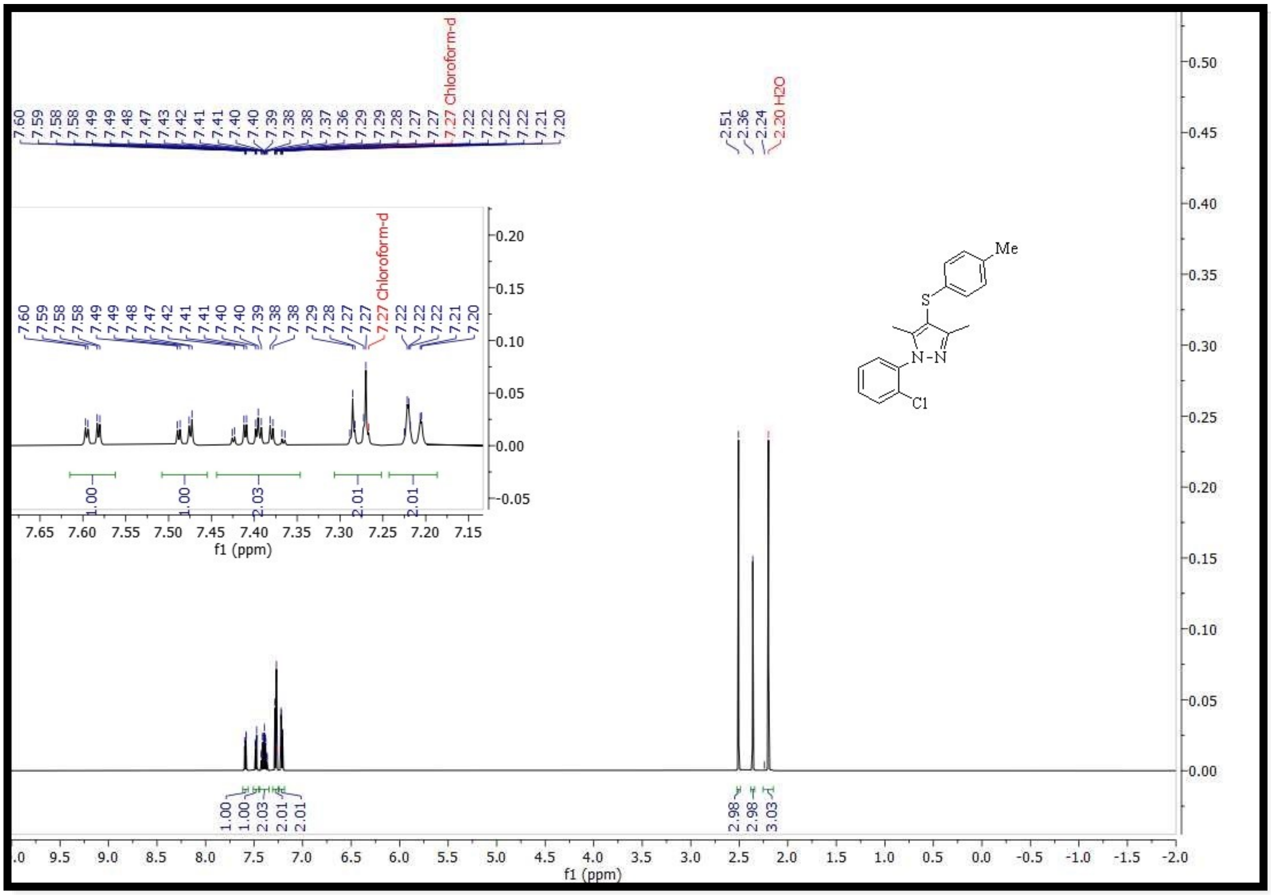

Figure 15: ^{1}H-NMR spectrum of compound **4h**

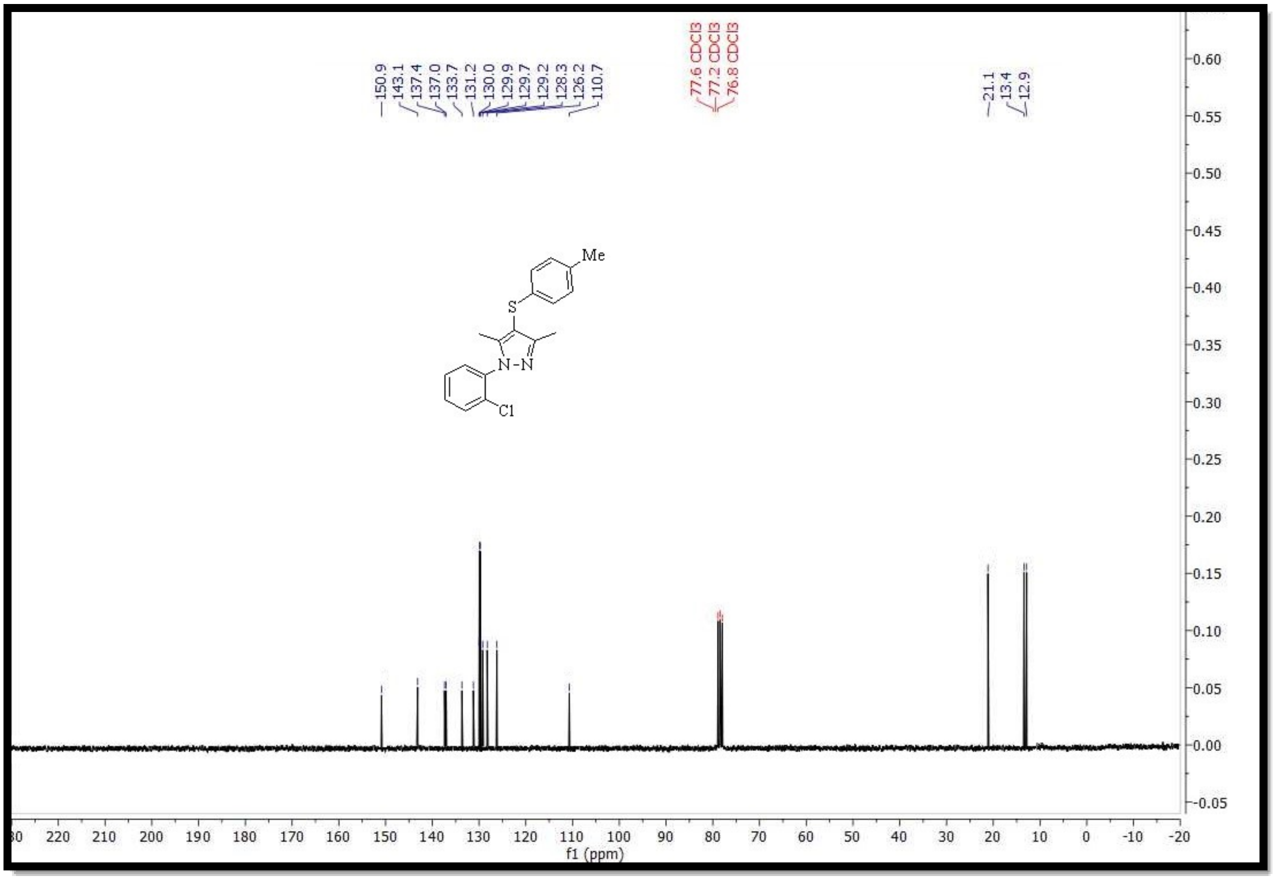

Figure 16: ^{13}C-NMR spectrum of compound **4h**

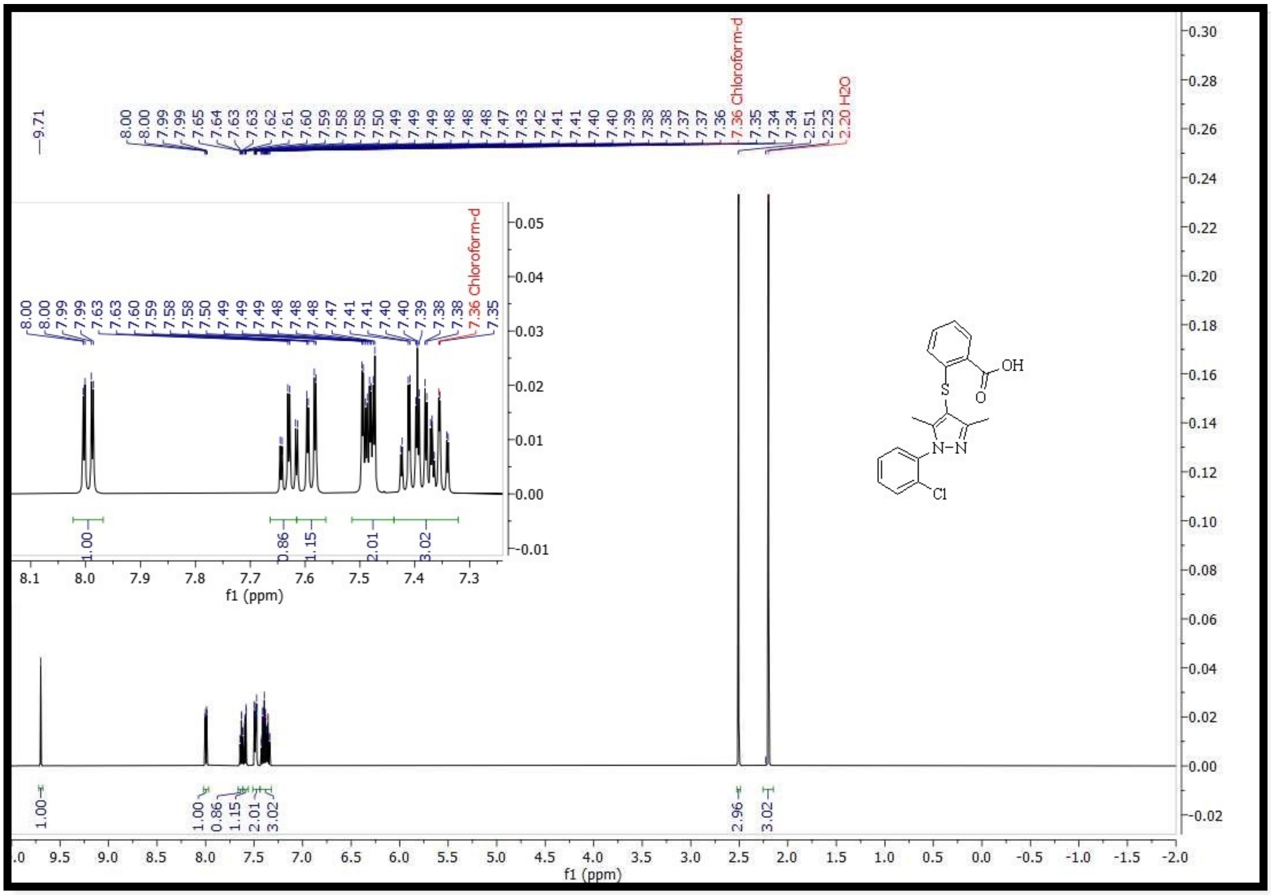

Figure 17: ^{1}H-NMR spectrum of compound **4i**

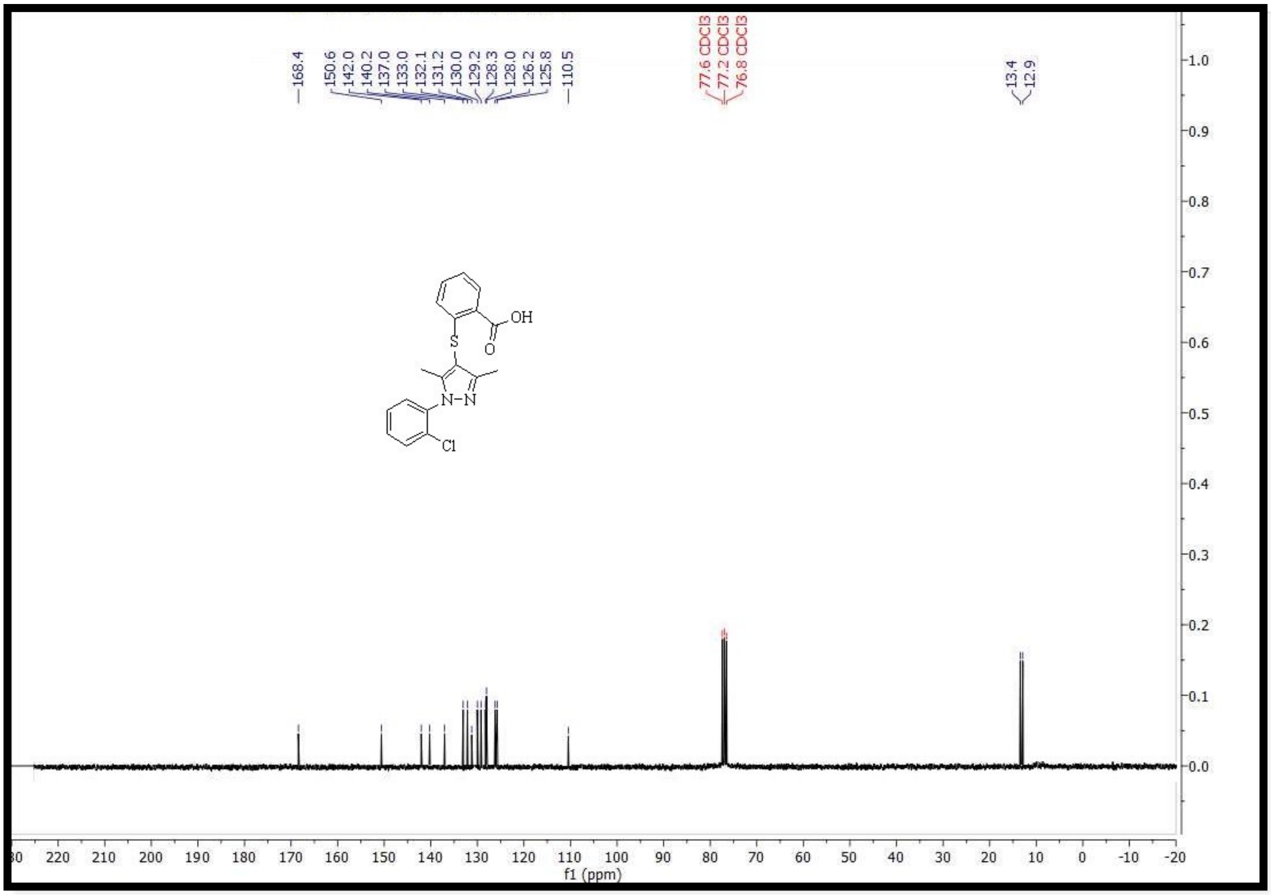

Figure 18: ^{13}C-NMR spectrum of compound **4i**

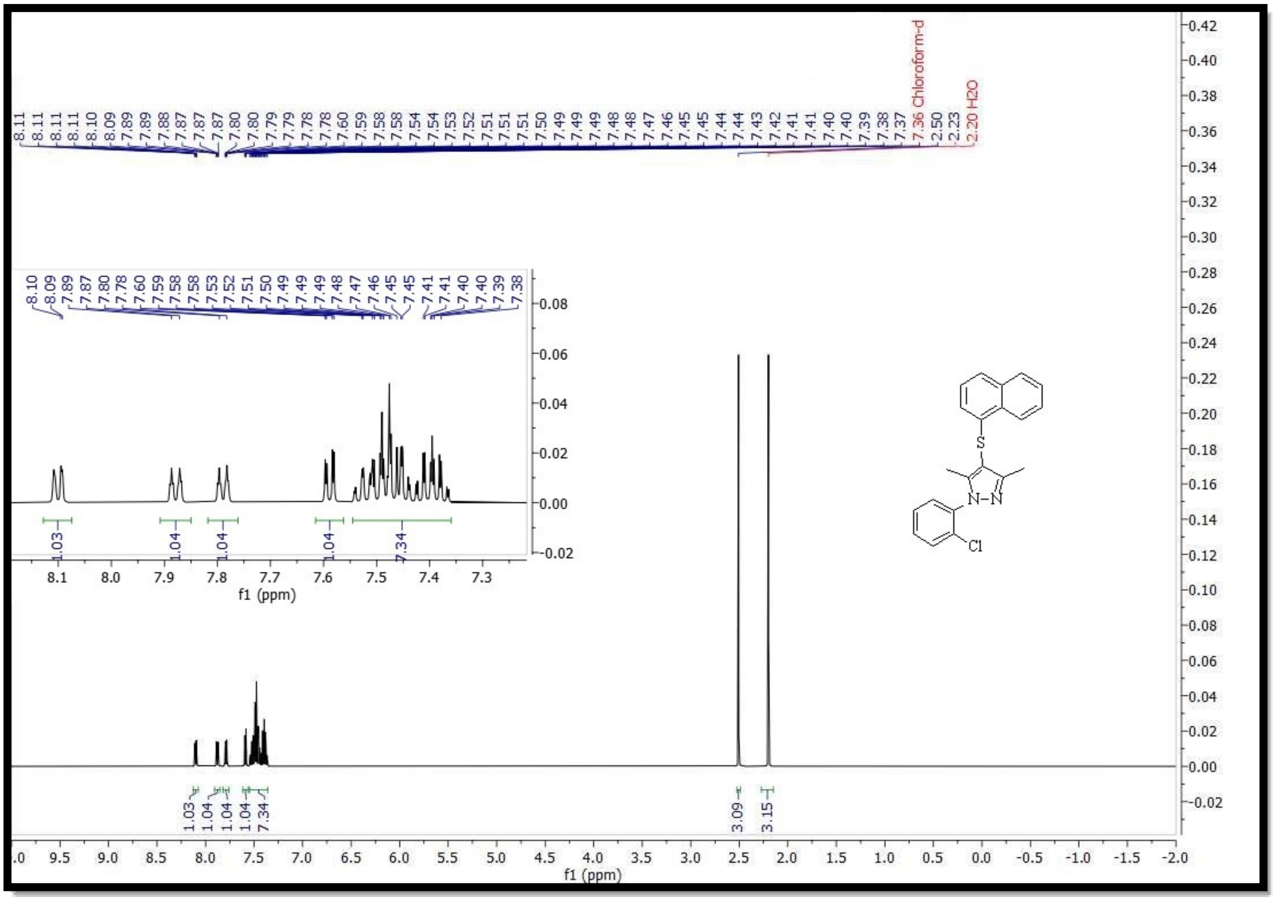

Figure 19: ^{1}H-NMR spectrum of compound **4j**

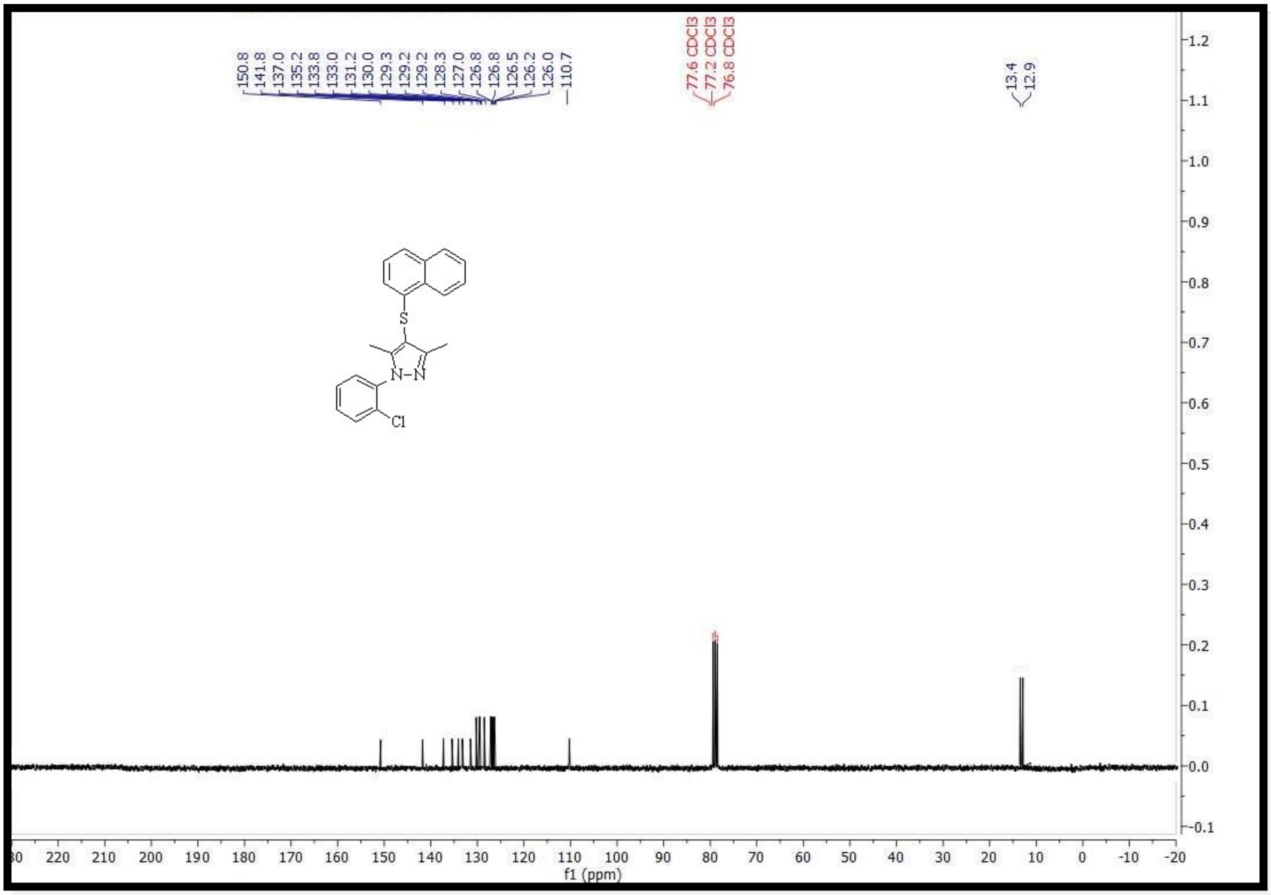

Figure 20: ^{13}C-NMR spectrum of compound **4j**

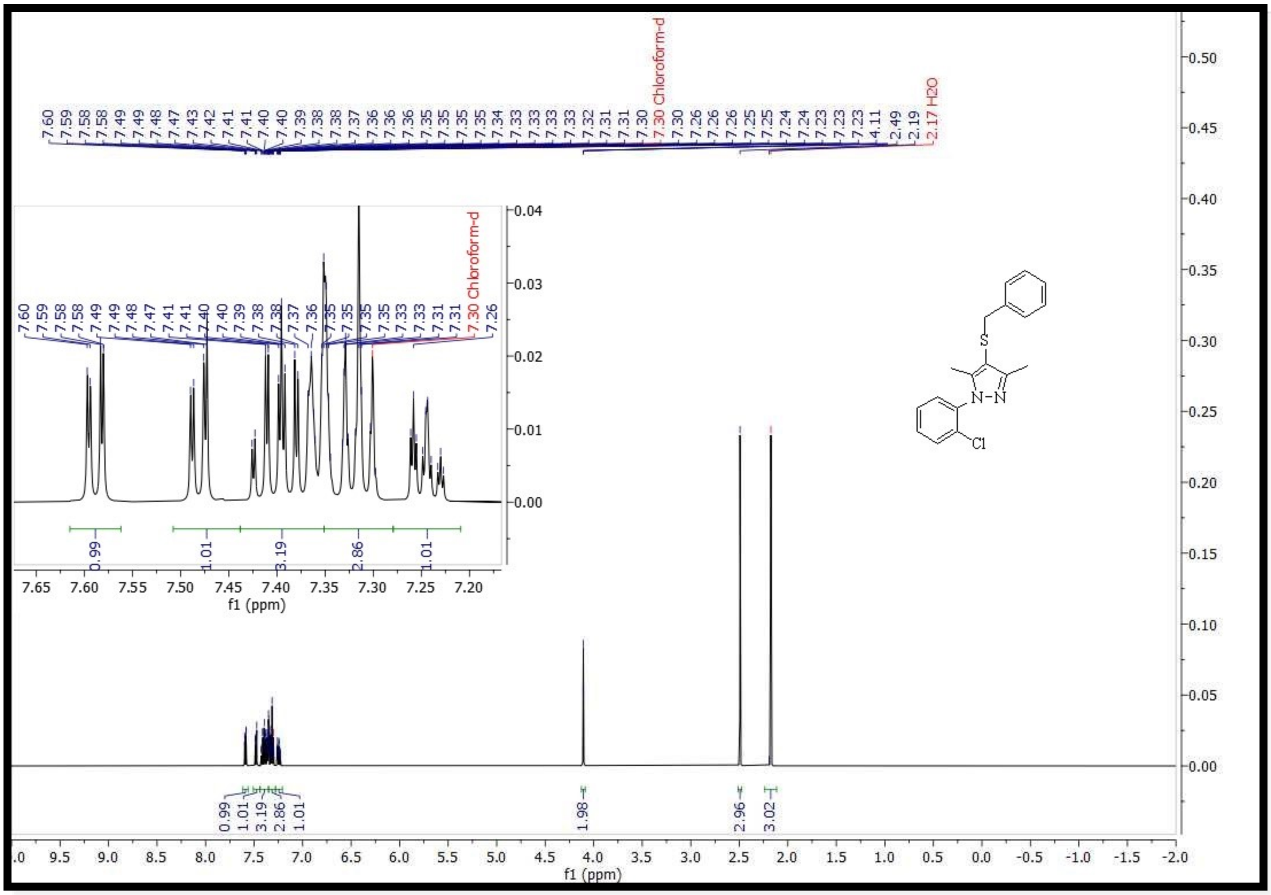

Figure 21: ^{1}H-NMR spectrum of compound **4k**

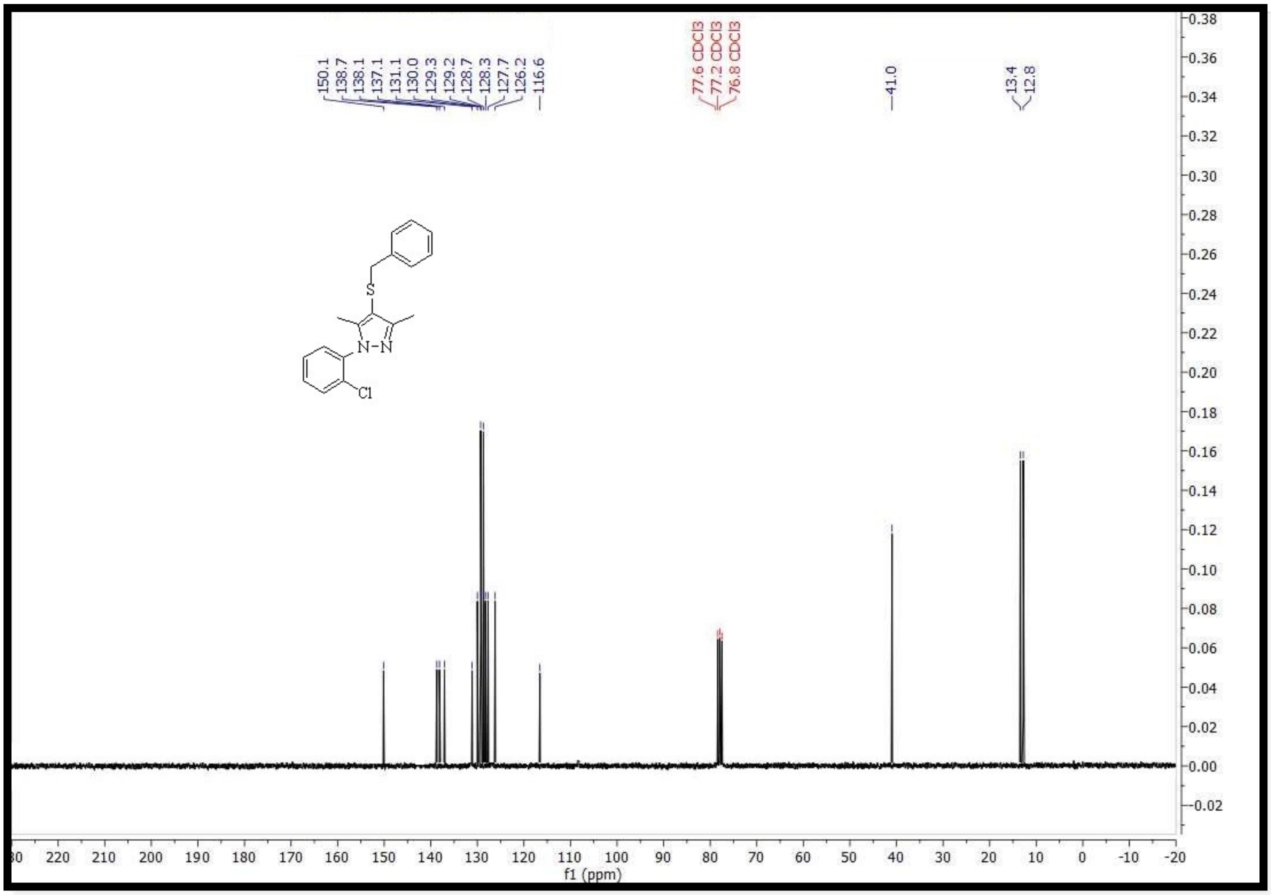

Figure 22: ^{13}C-NMR spectrum of compound **4k**

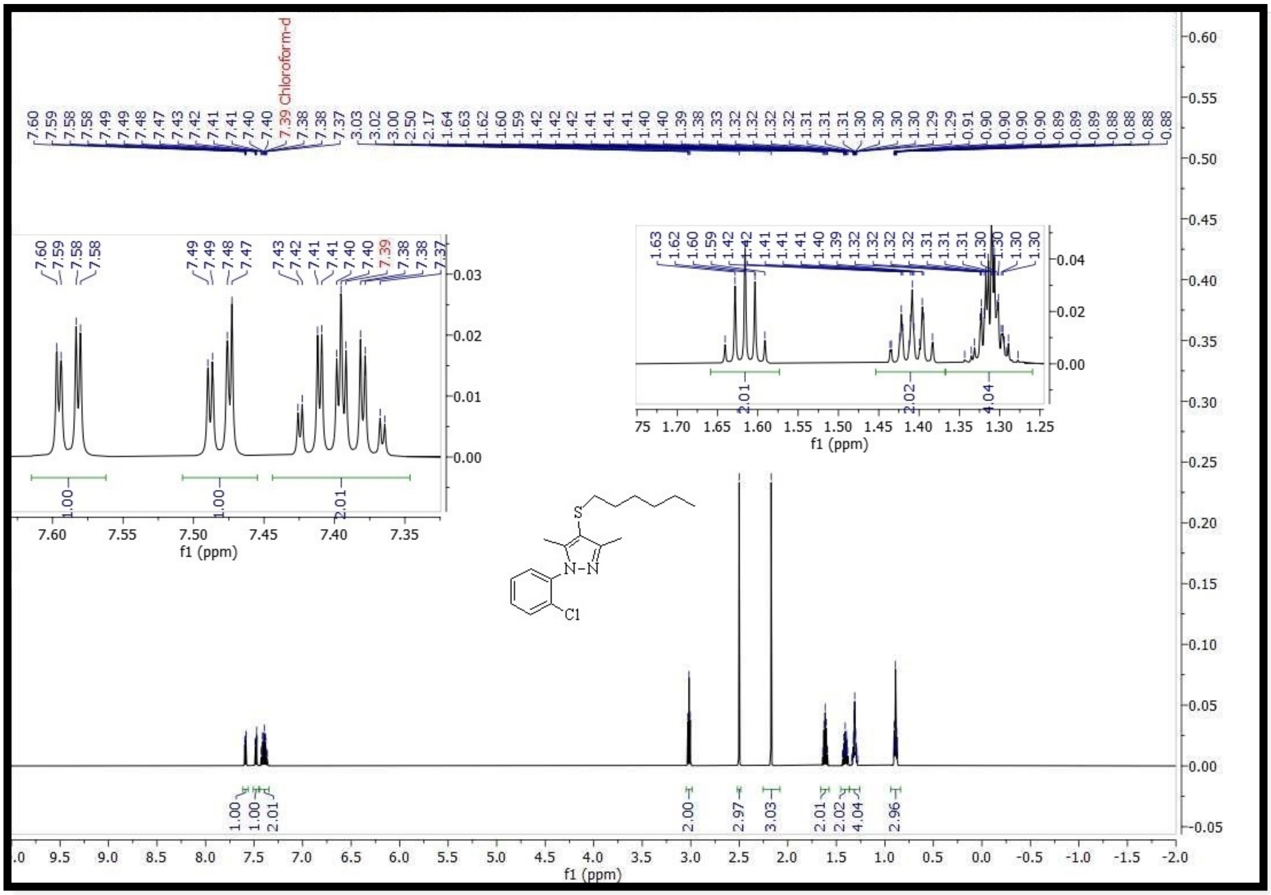

Figure 23: ^{1}H-NMR spectrum of compound **4l**

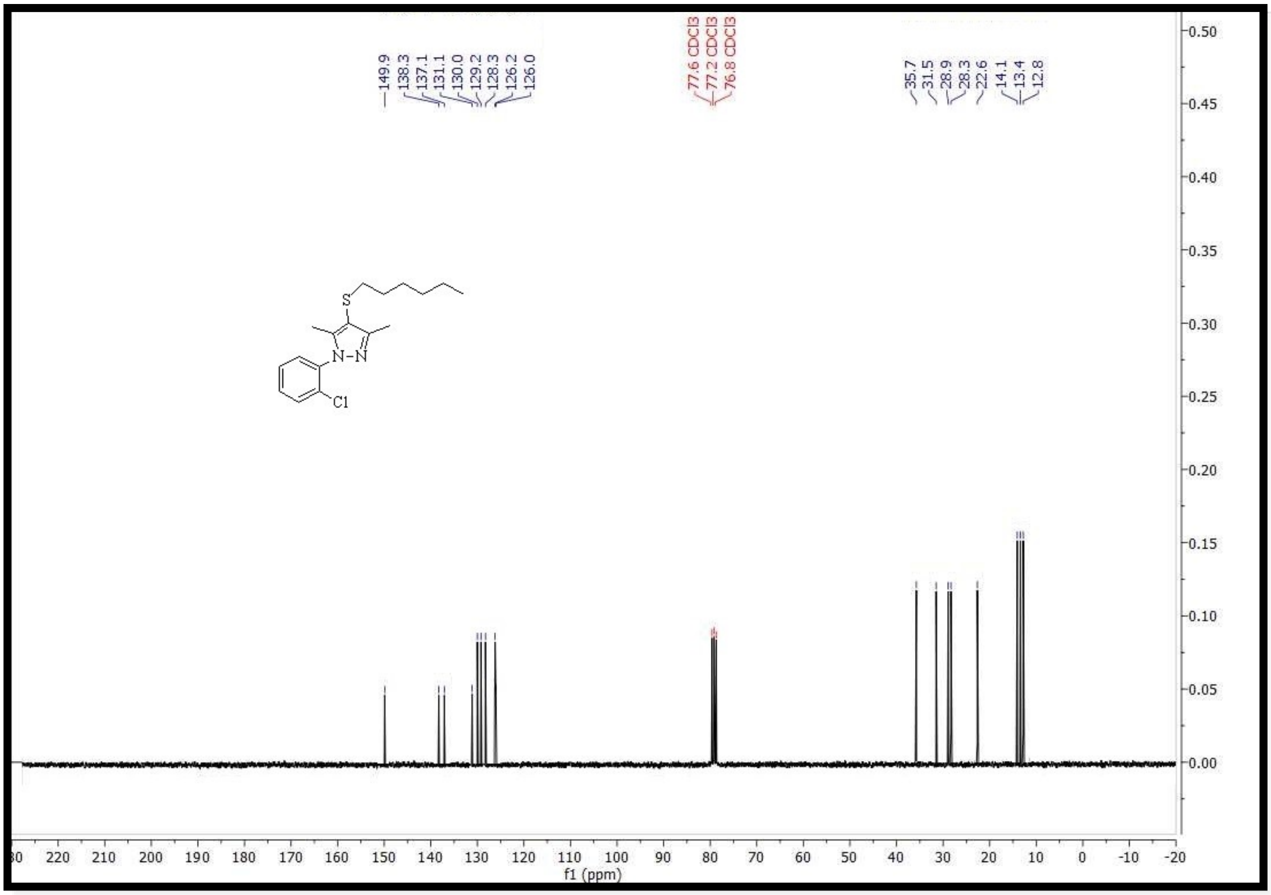

Figure 24: ^{13}C-NMR spectrum of compound **4l**

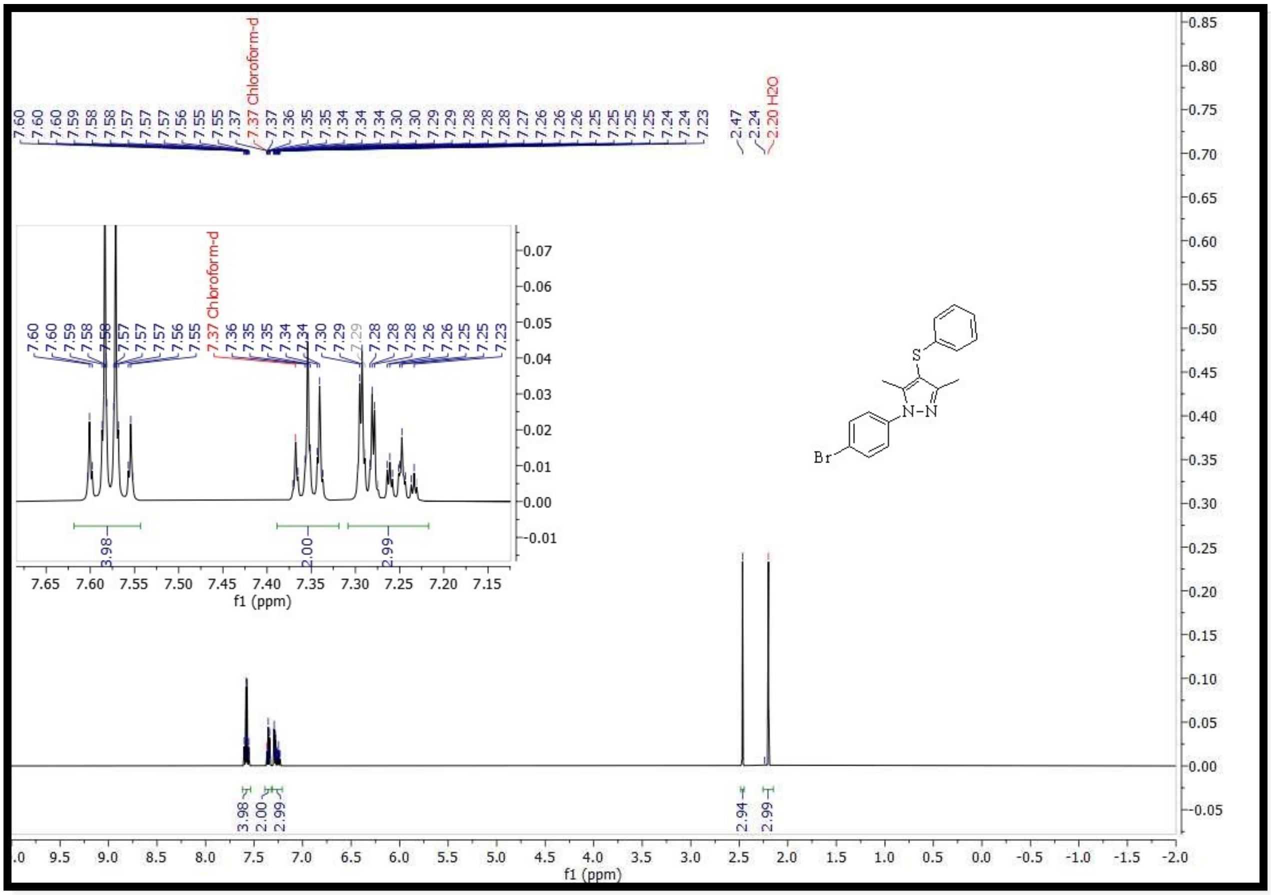

Figure 25: ^{1}H-NMR spectrum of compound **4m**

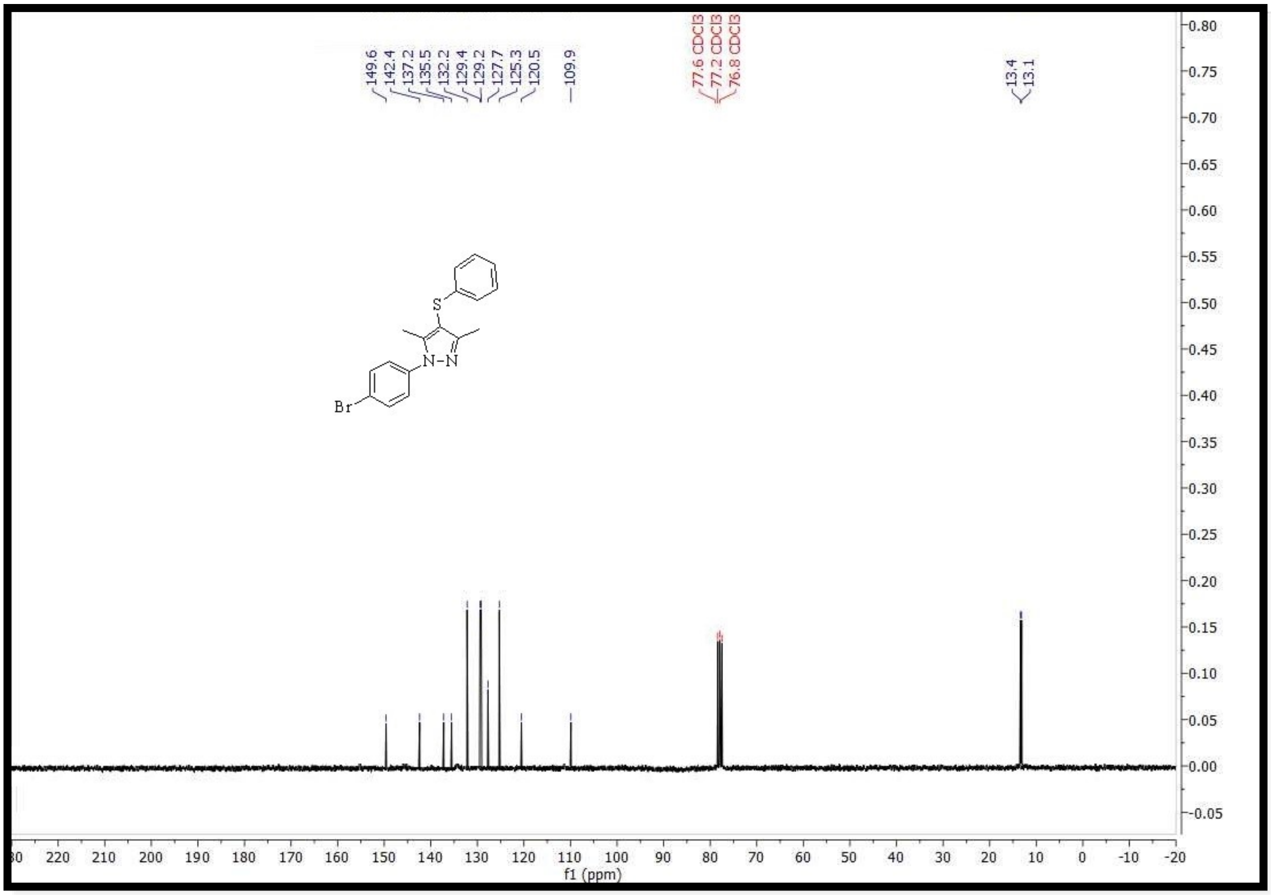

Figure 26: ^{13}C-NMR spectrum of compound **4m**

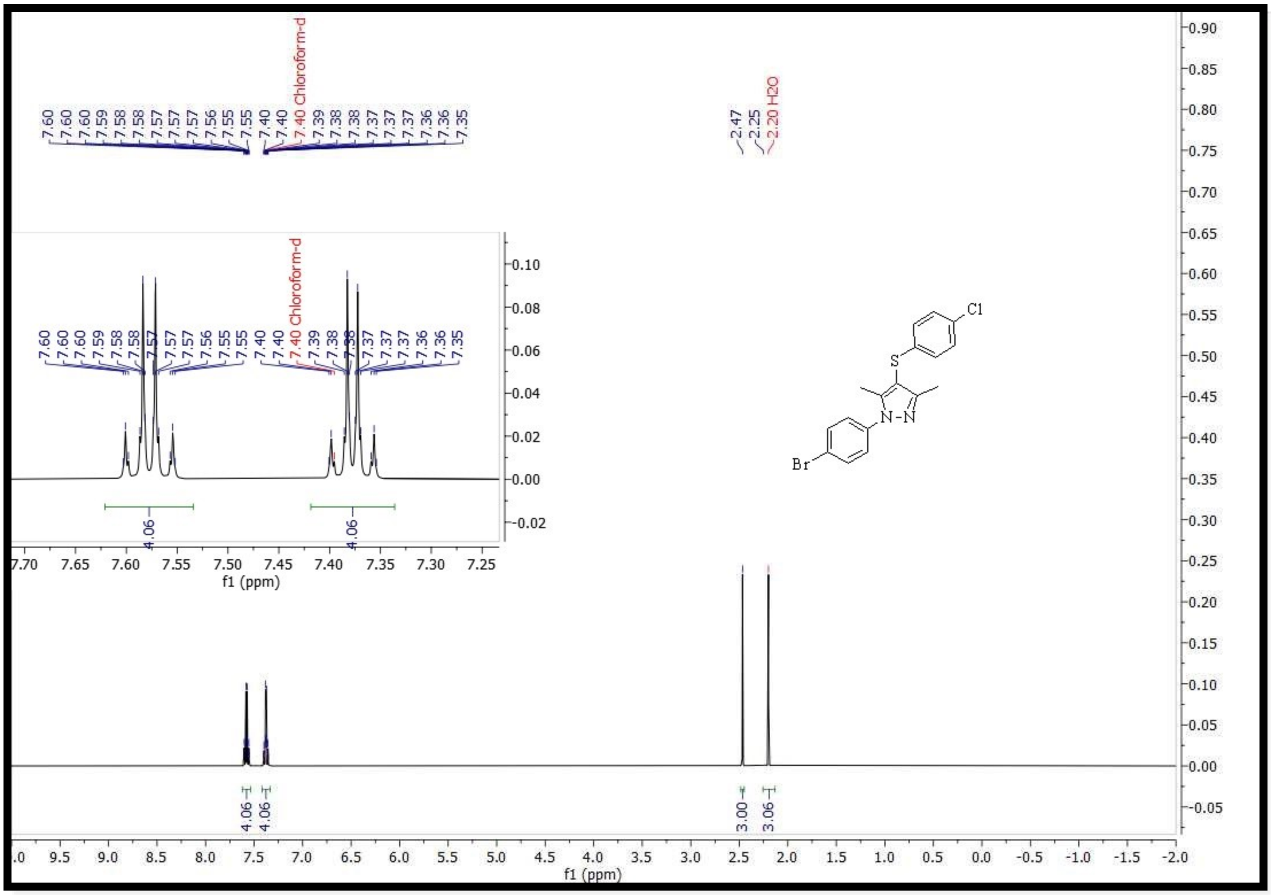

Figure 27: ^{1}H-NMR spectrum of compound **4n**

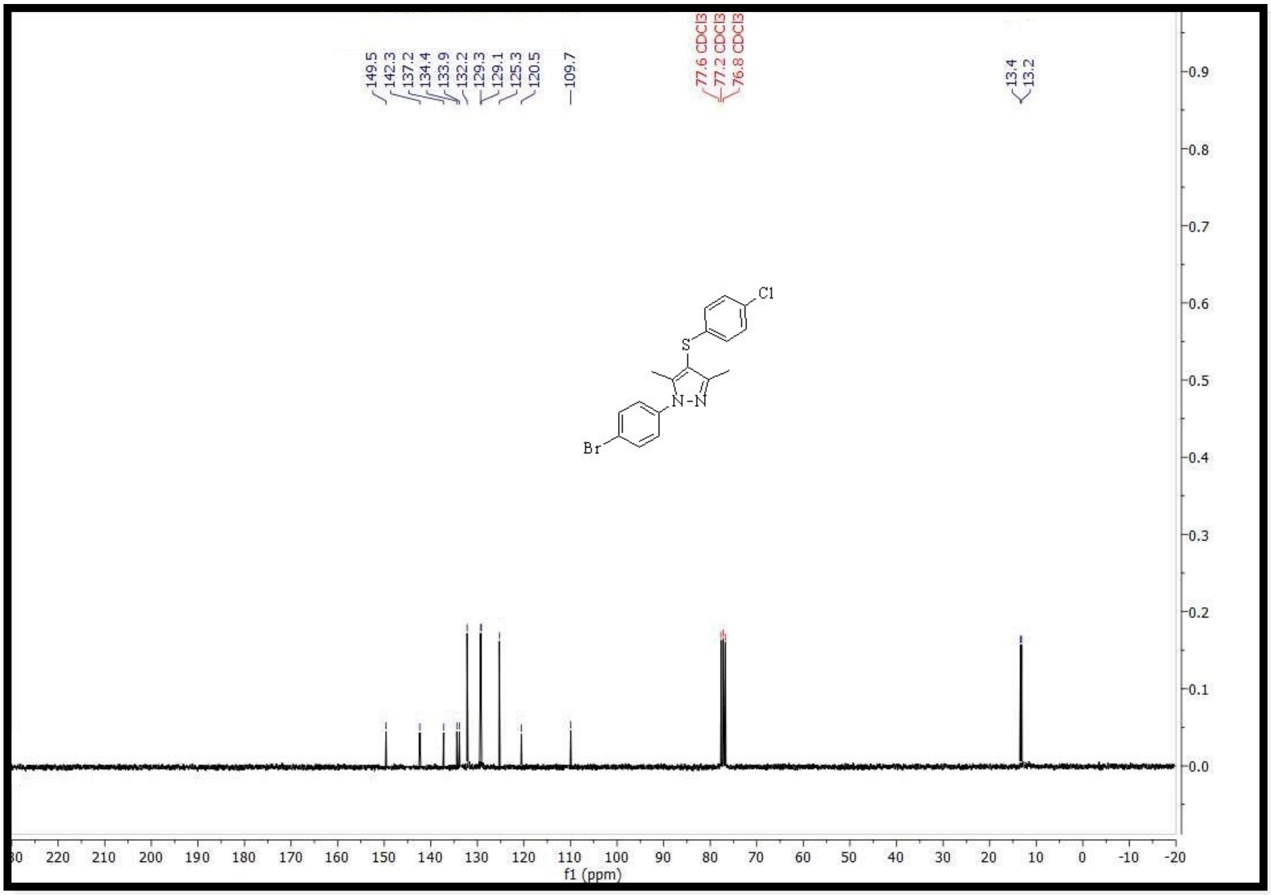

Figure 28: ^{13}C-NMR spectrum of compound **4n**

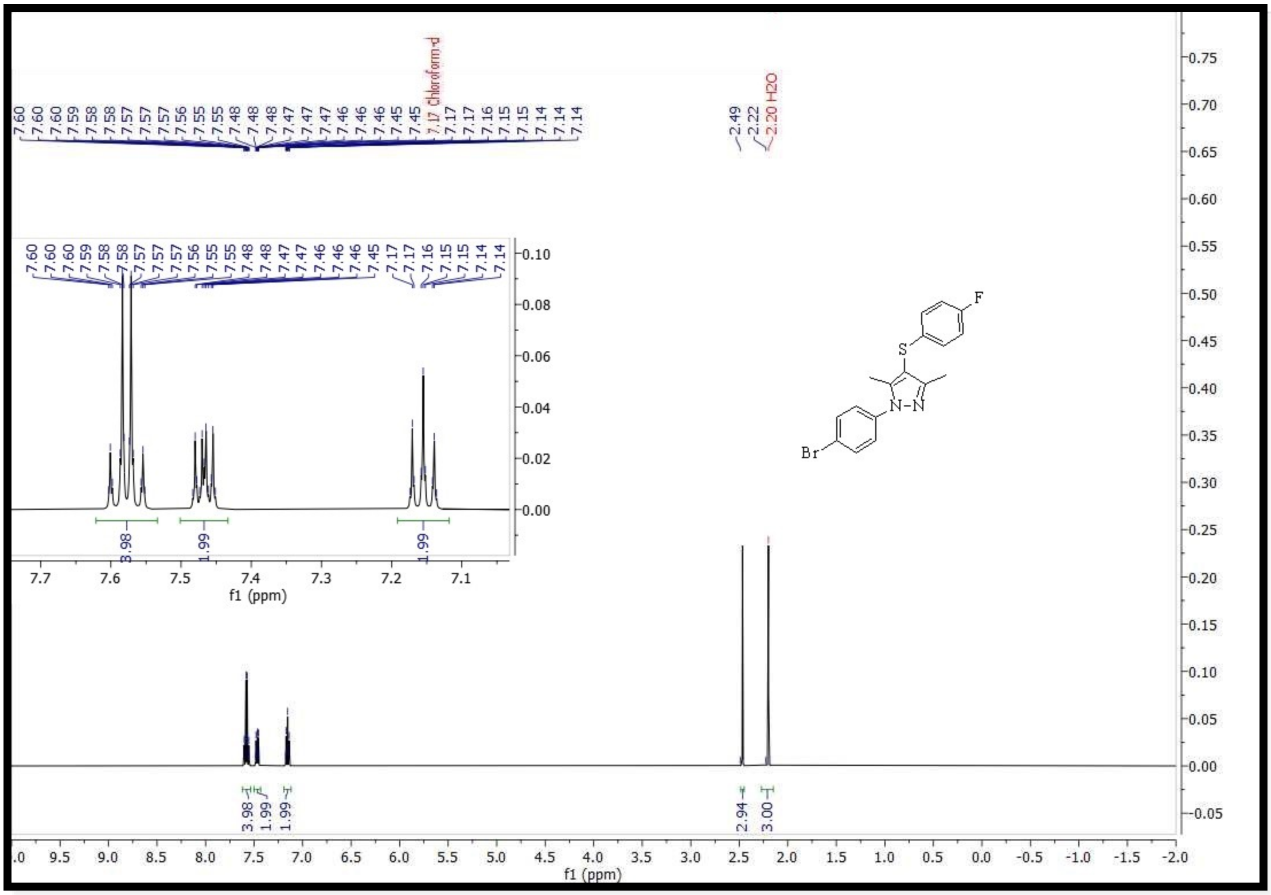

Figure 29: ^{1}H-NMR spectrum of compound **4o**

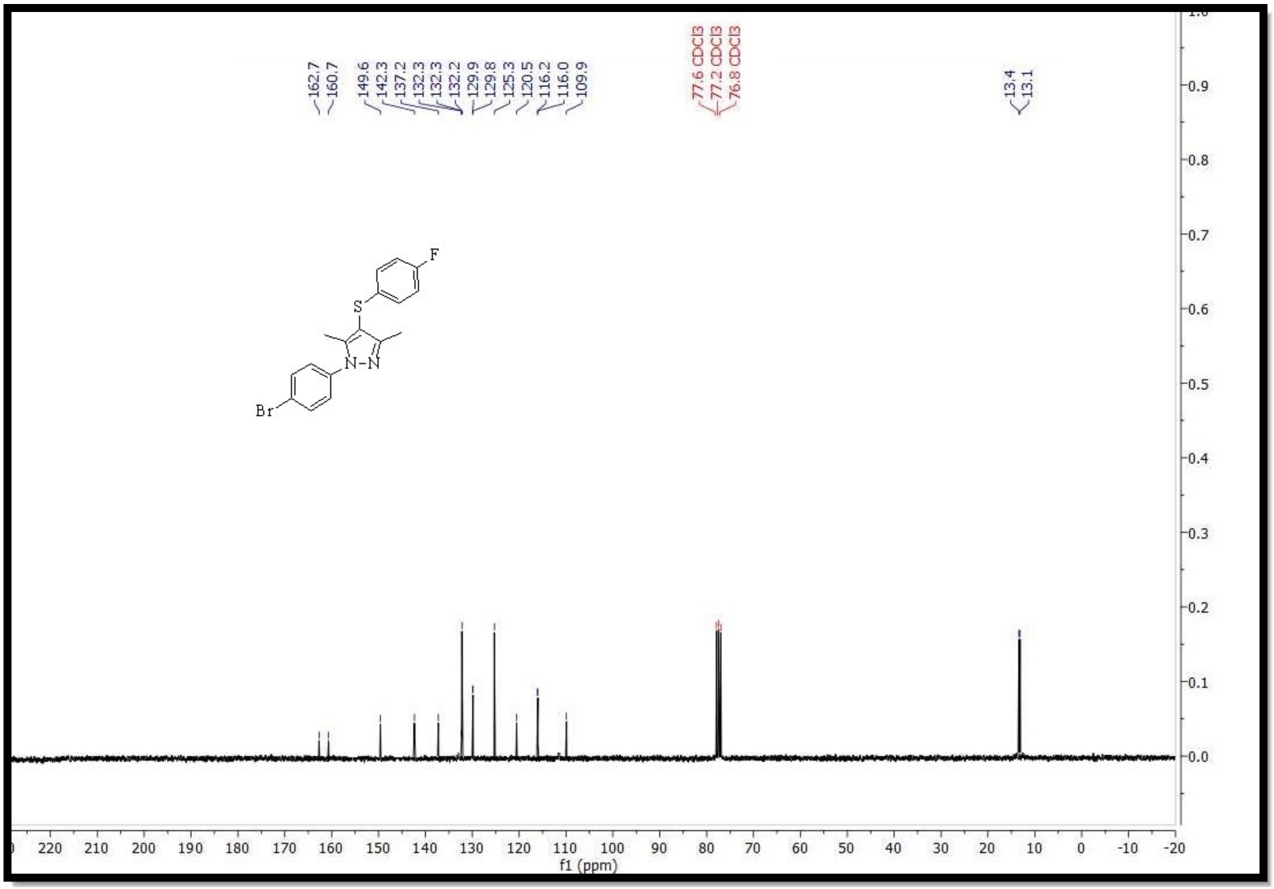

Figure 30: ^{13}C-NMR spectrum of compound **4o**

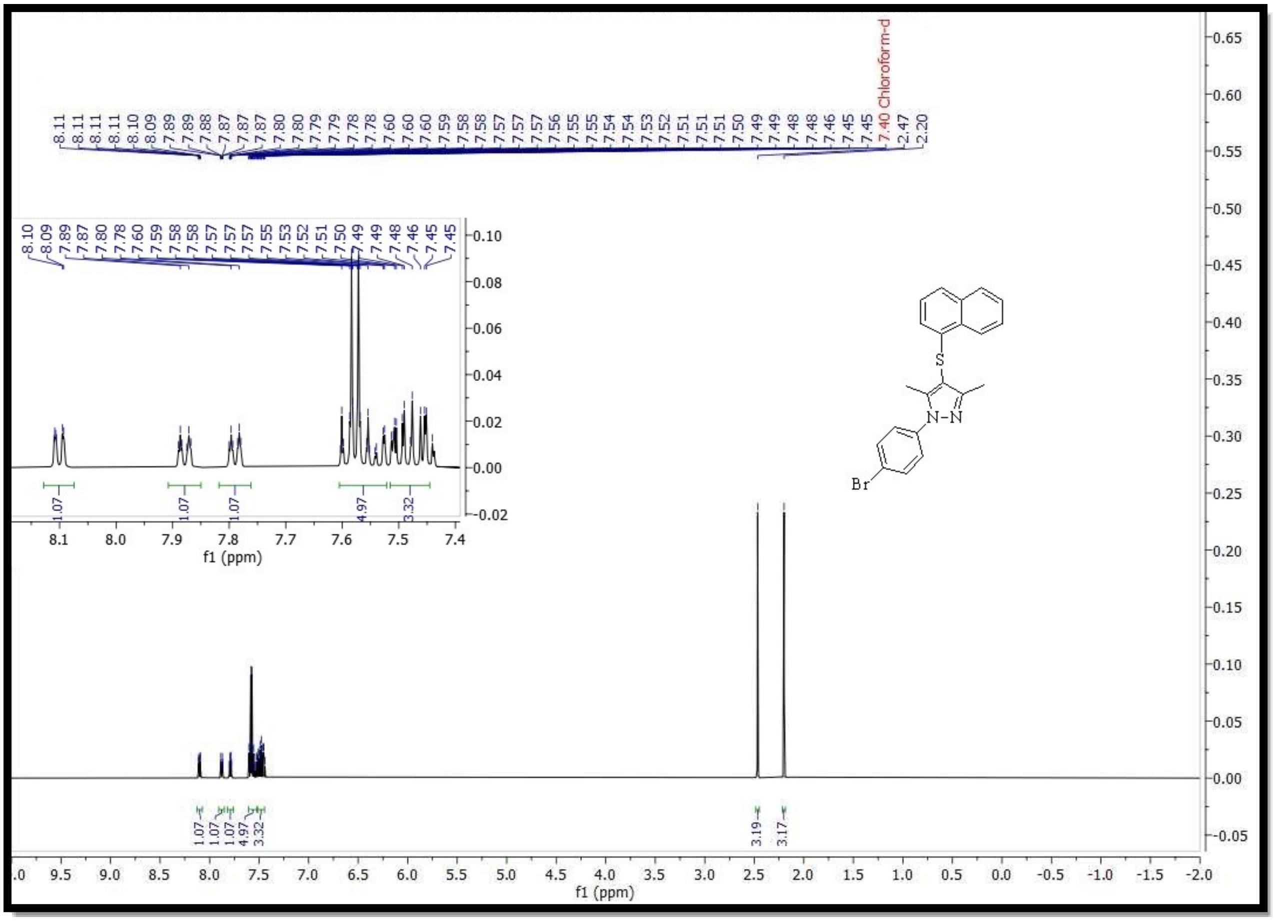

Figure 31: ^{1}H-NMR spectrum of compound **4p**

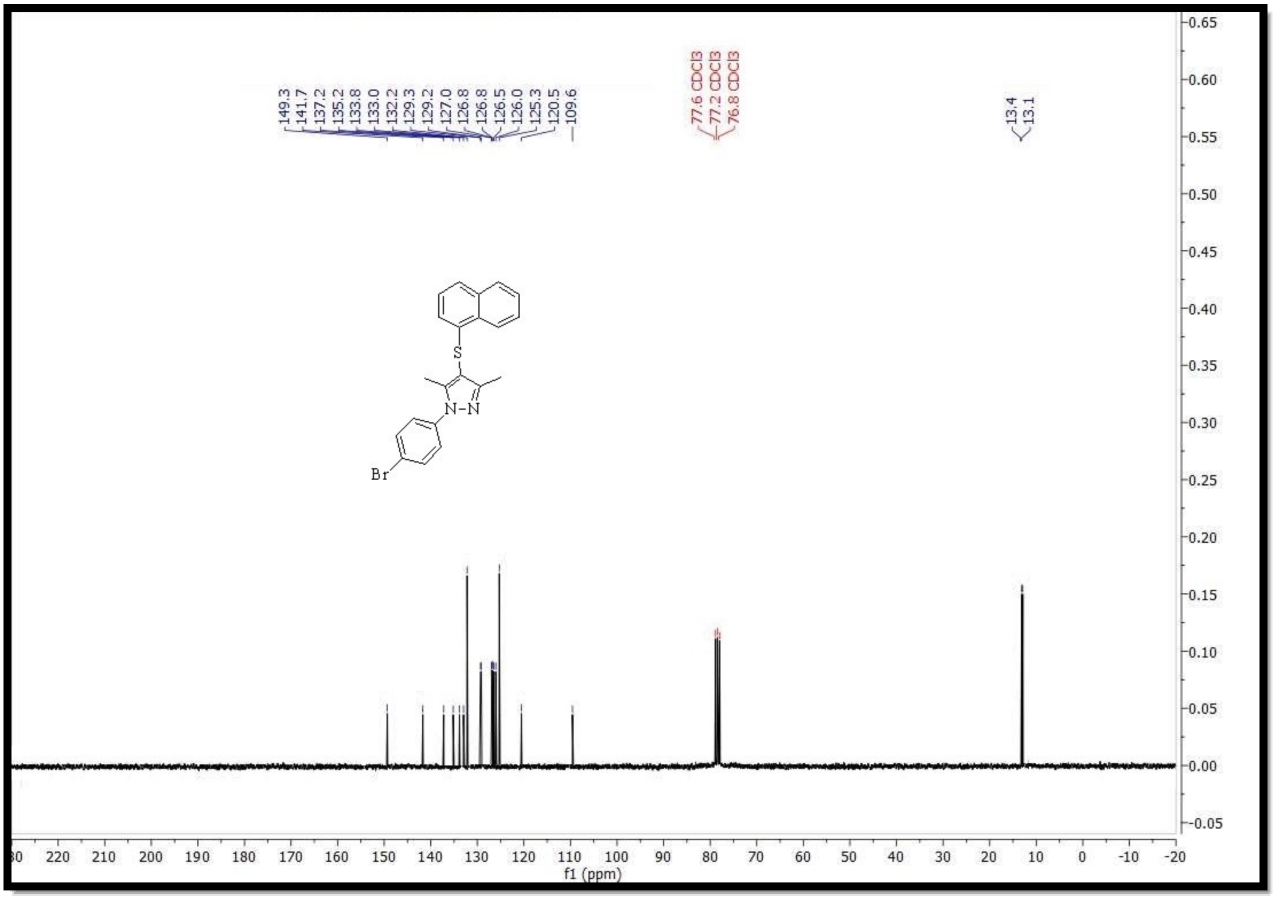

Figure 32: ^{13}C-NMR spectrum of compound **4p**

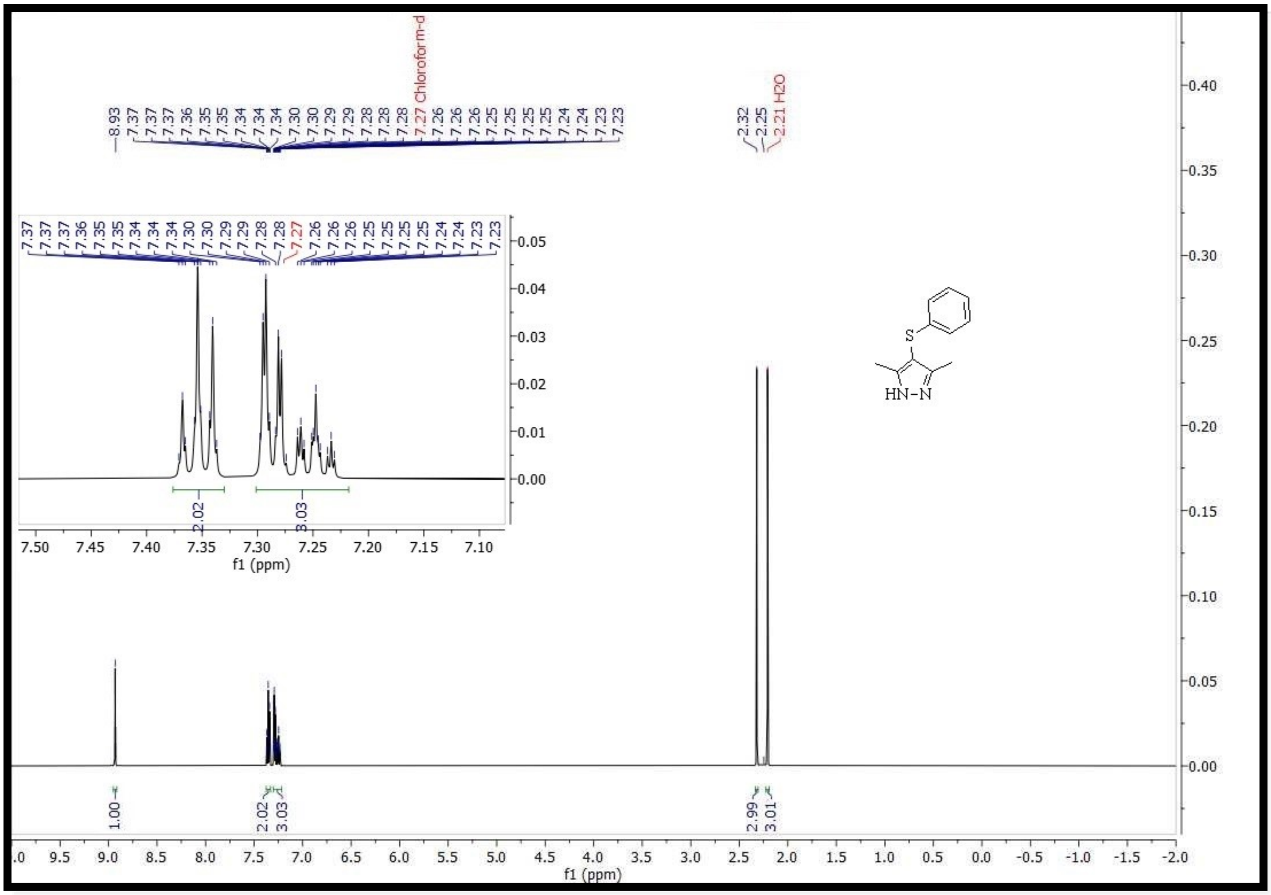

Figure 33: ^{1}H-NMR spectrum of compound **4q**

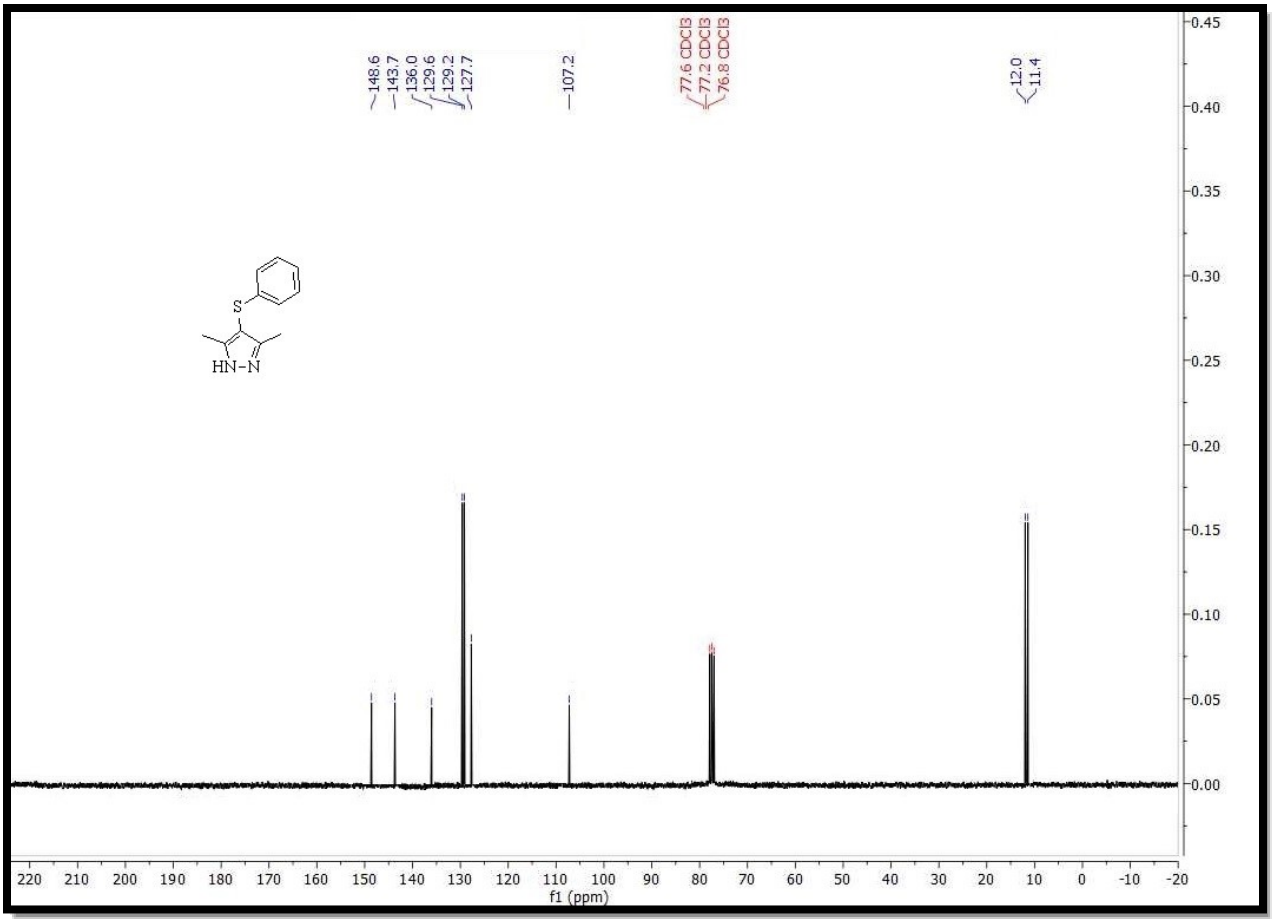

Figure 34: ^{13}C-NMR spectrum of compound **4q**

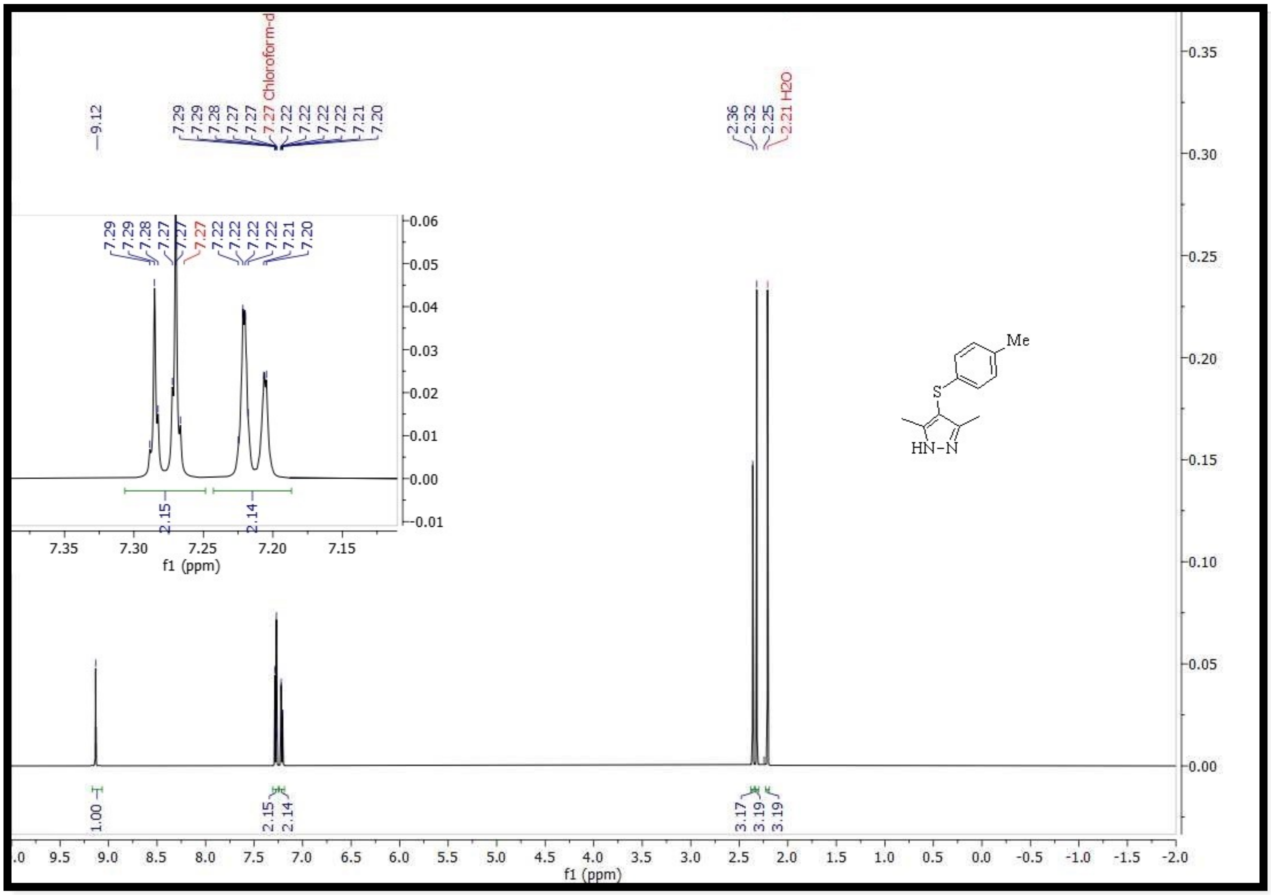

Figure 35: ^{1}H-NMR spectrum of compound **4r**

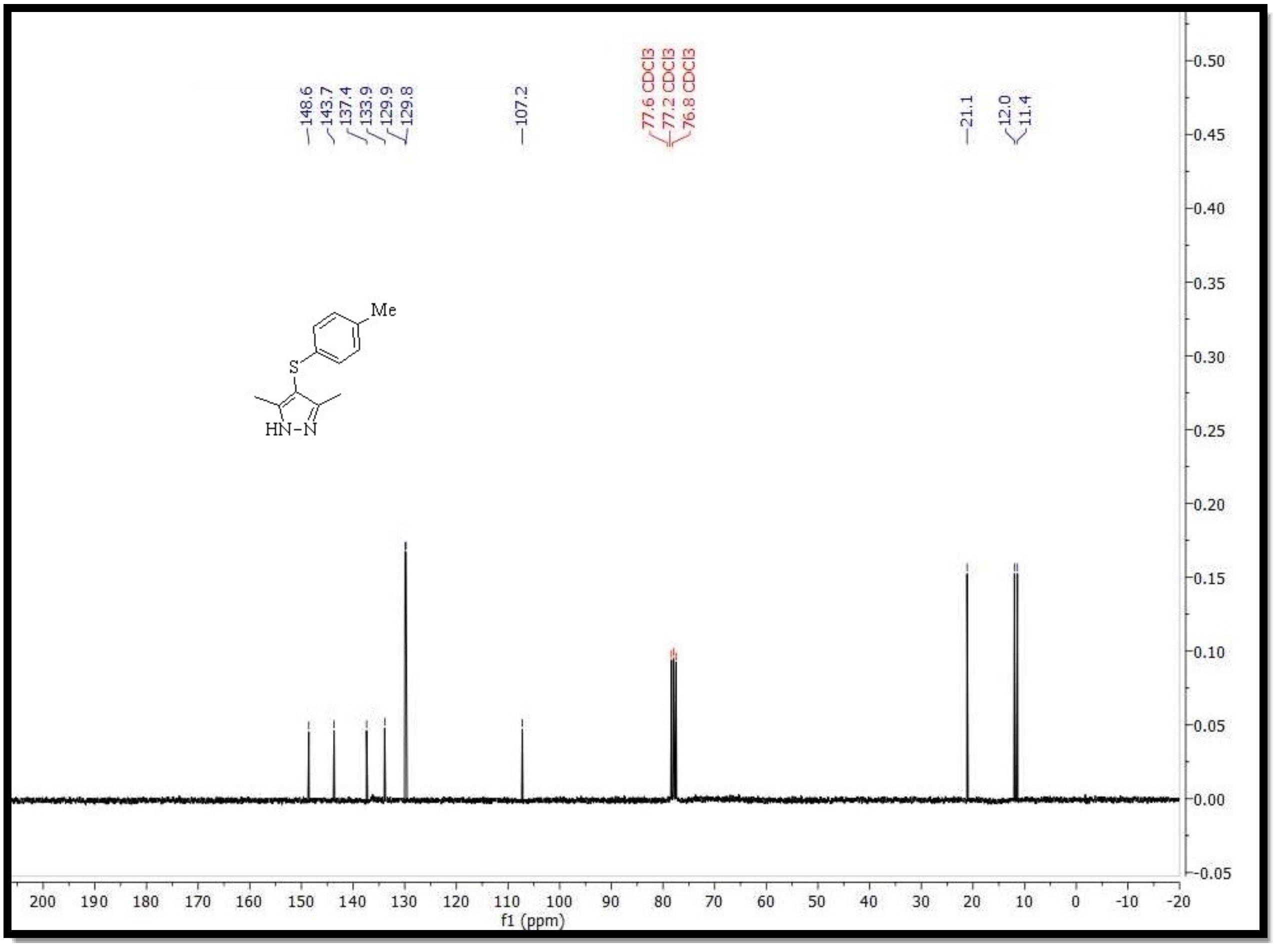

Figure 36: ^{13}C-NMR spectrum of compound **4r**

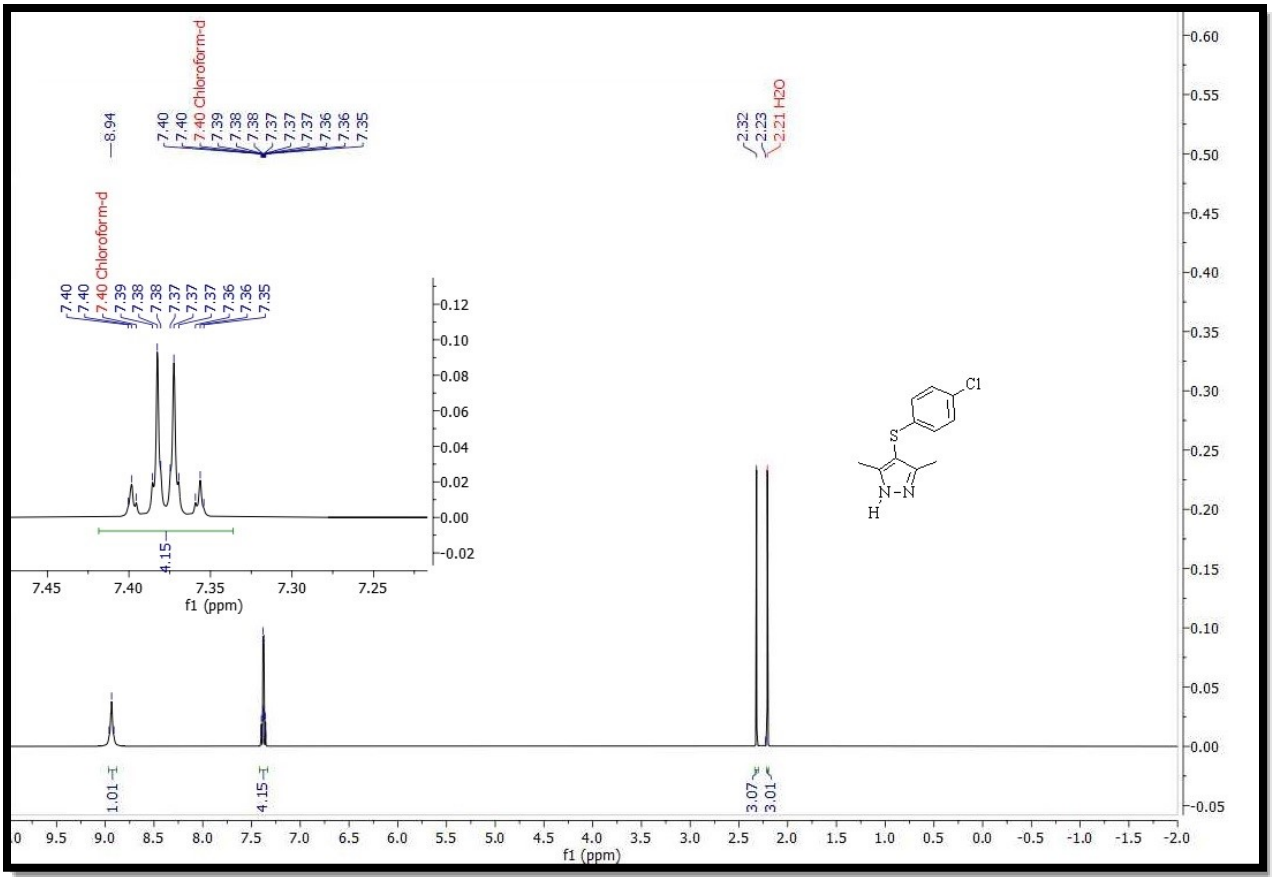

Figure 37: ^{1}H-NMR spectrum of compound **4s**

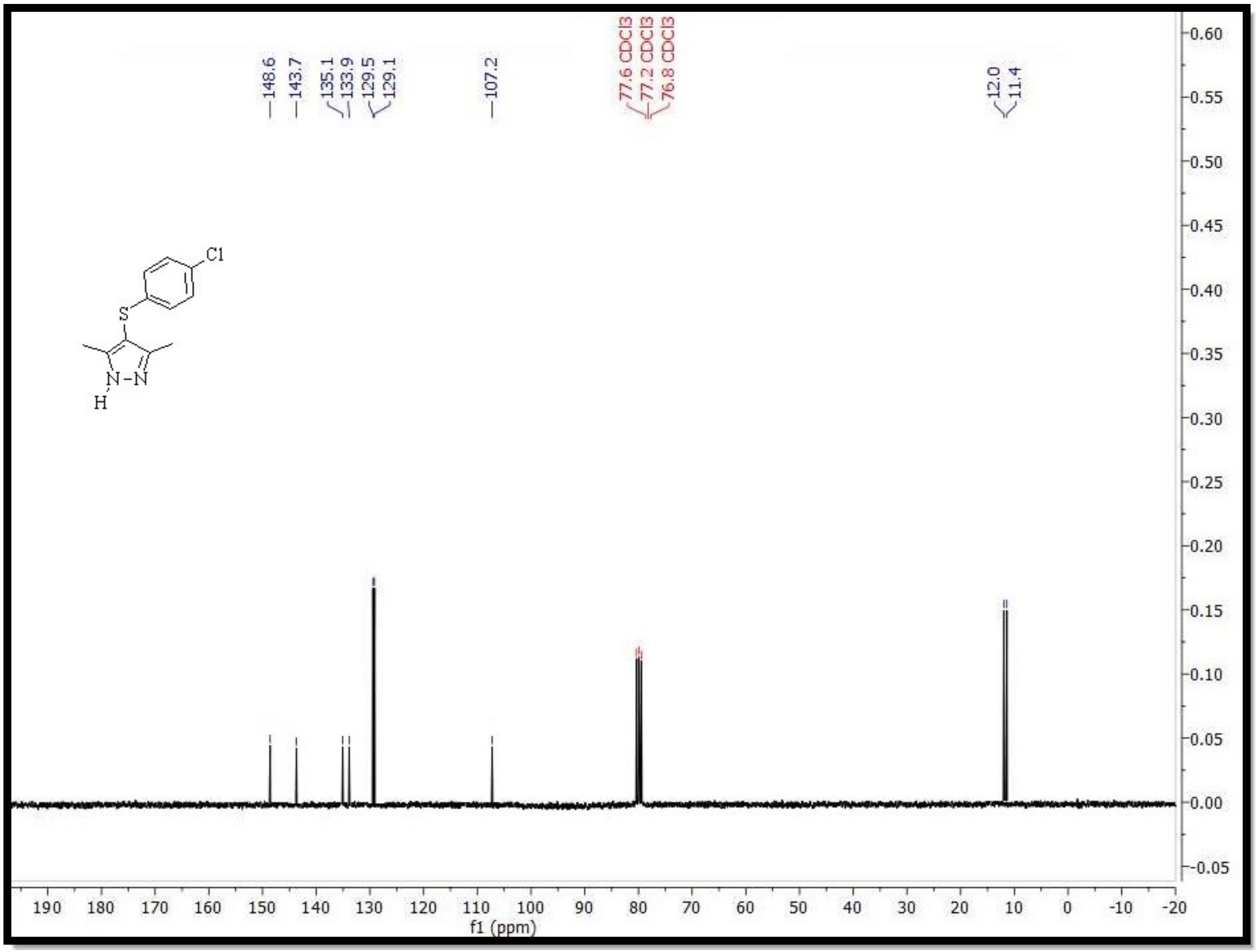

Figure 38: ^{13}C-NMR spectrum of compound **4s**

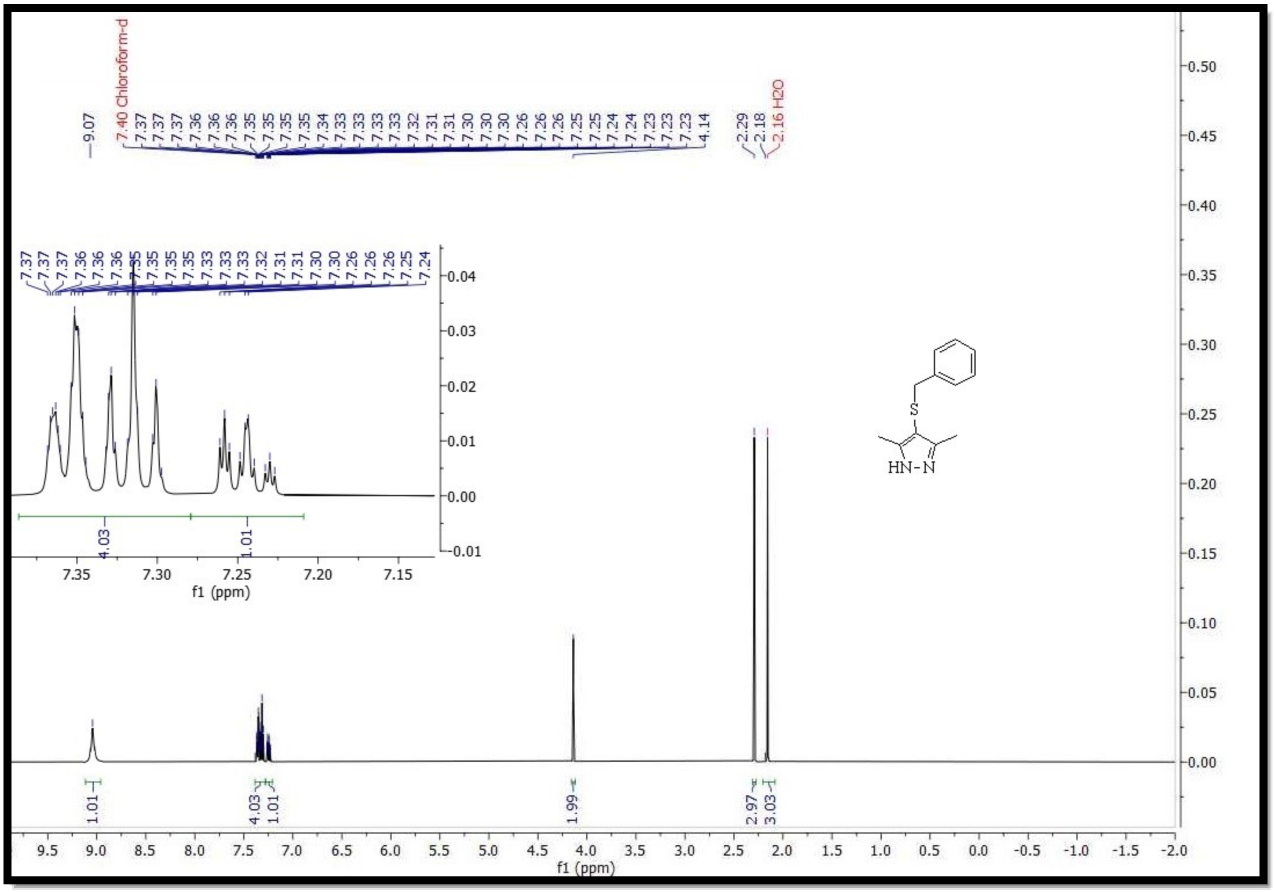

Figure 39: ^{1}H-NMR spectrum of compound **4t**

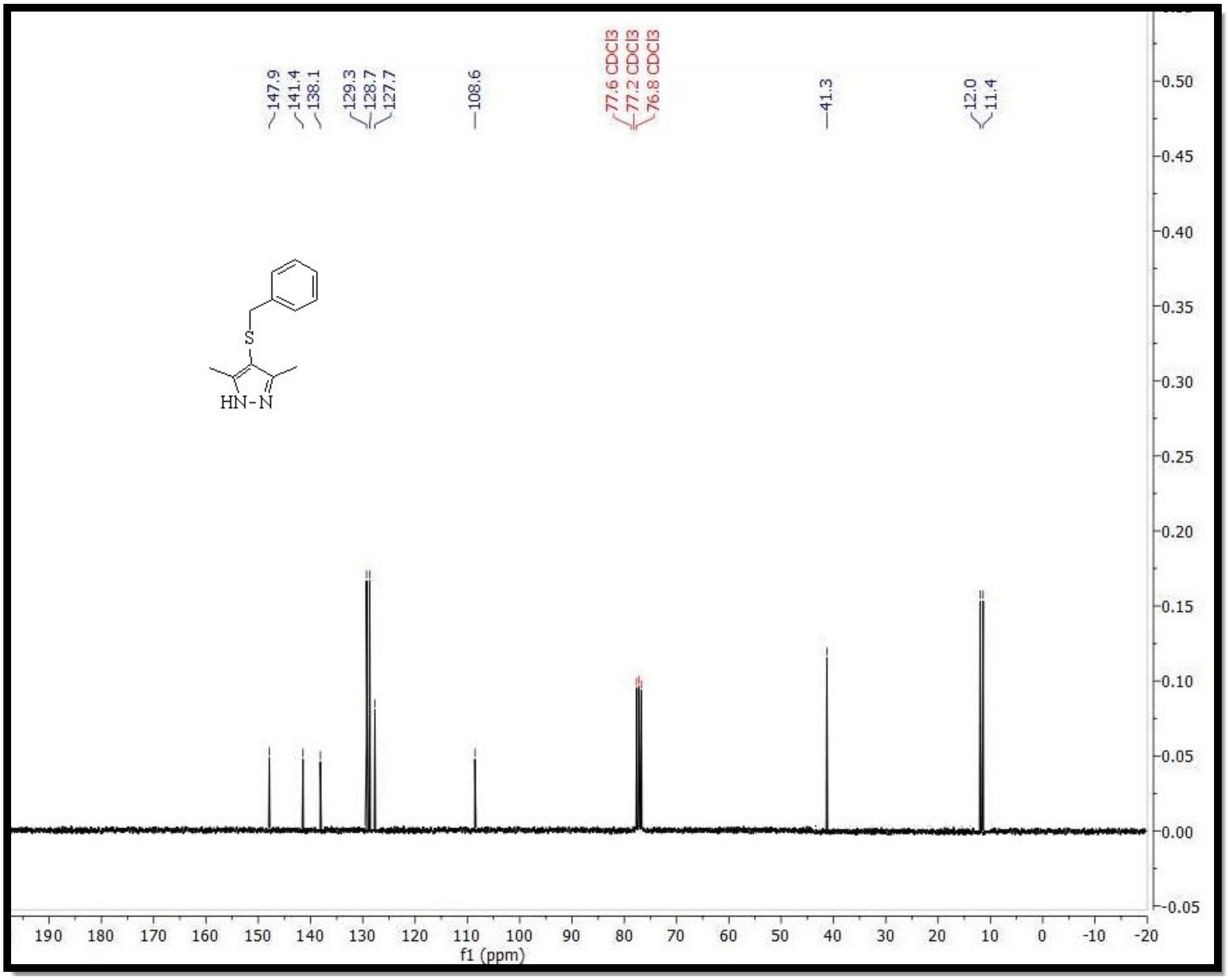

Figure 40: ^{13}C-NMR spectrum of compound **4t**

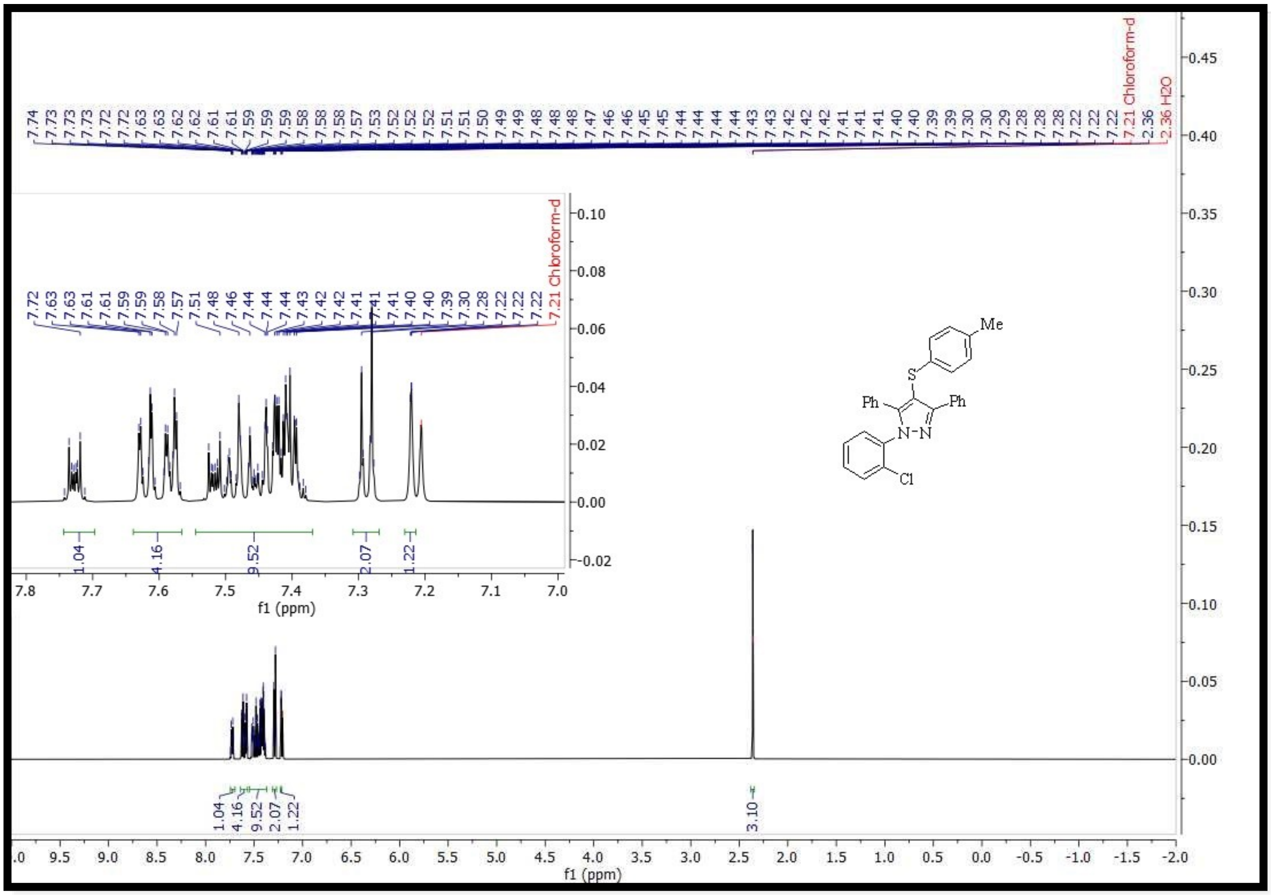

Figure 41: ^{1}H-NMR spectrum of compound **4u**

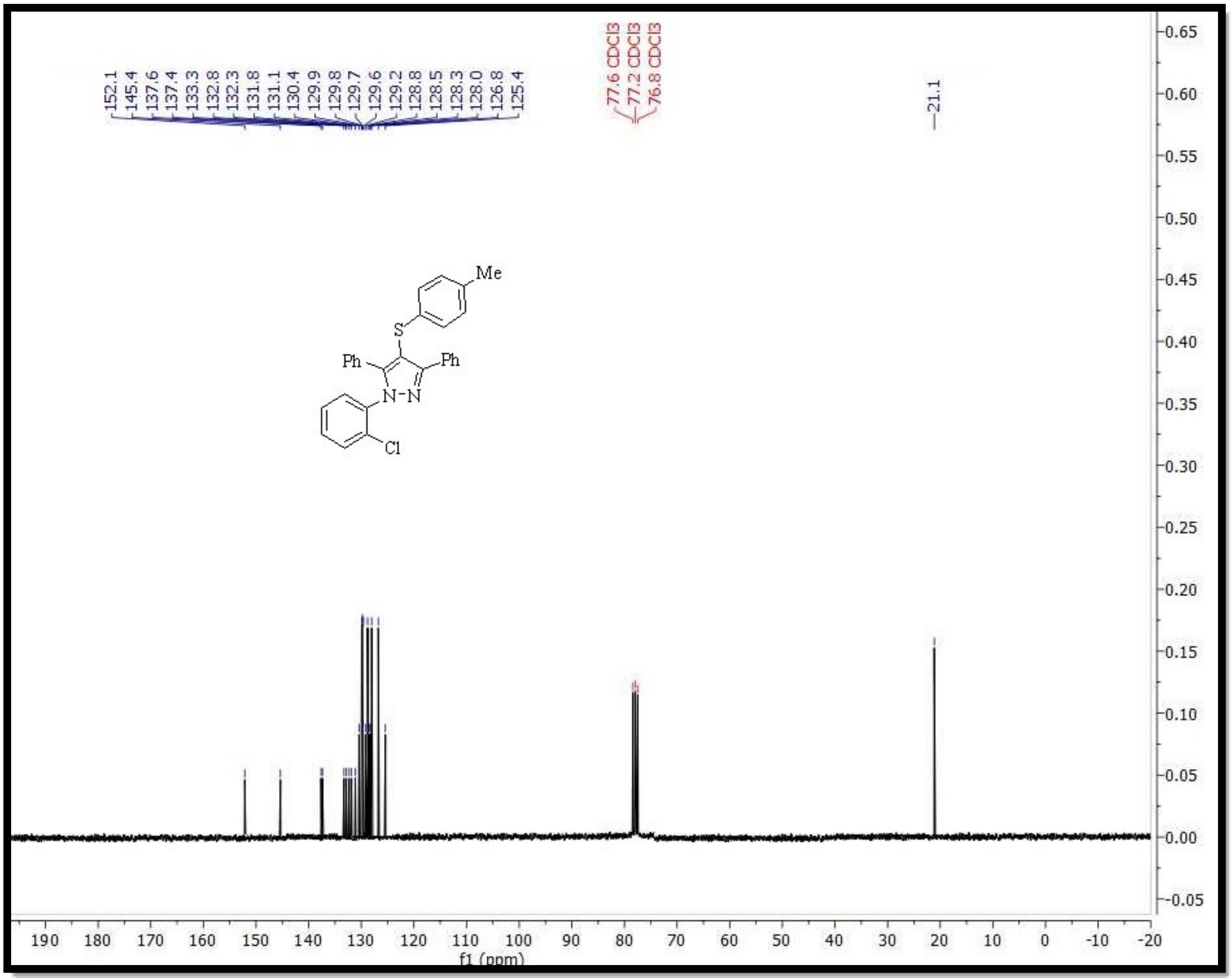

Figure 42: ^{13}C-NMR spectrum of compound **4u**

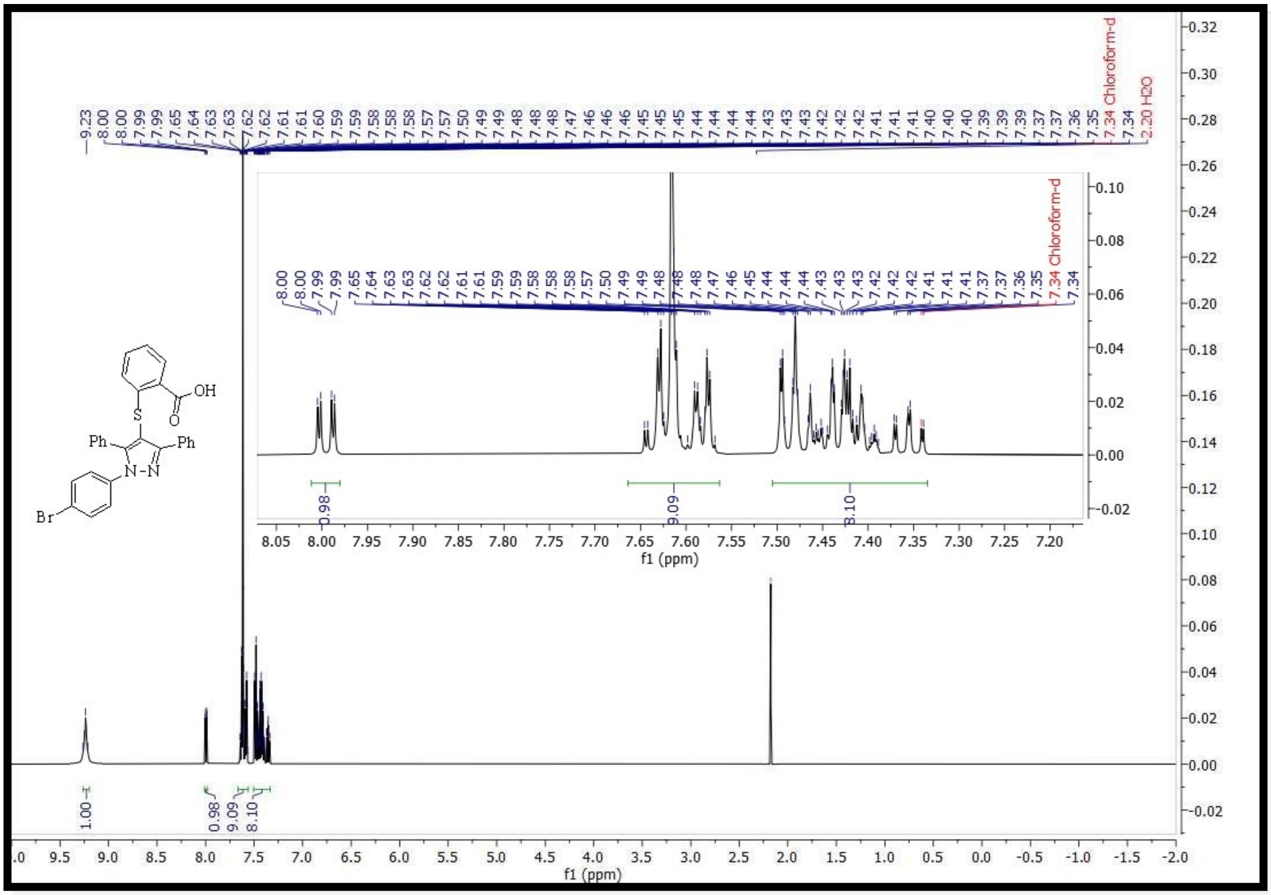

Figure 43: ^{1}H-NMR spectrum of compound **4v**

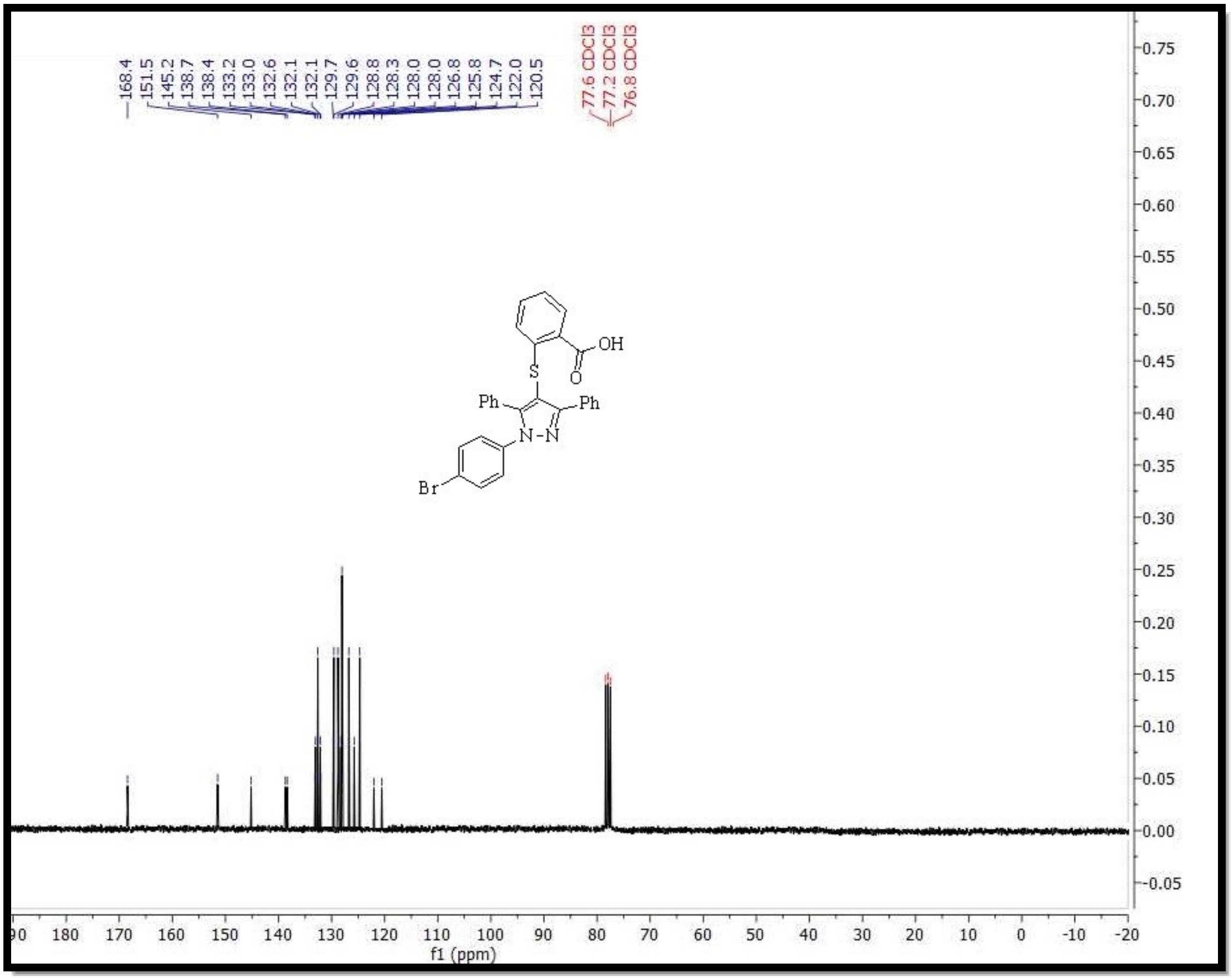

Figure 44: ^{13}C-NMR spectrum of compound **4v**

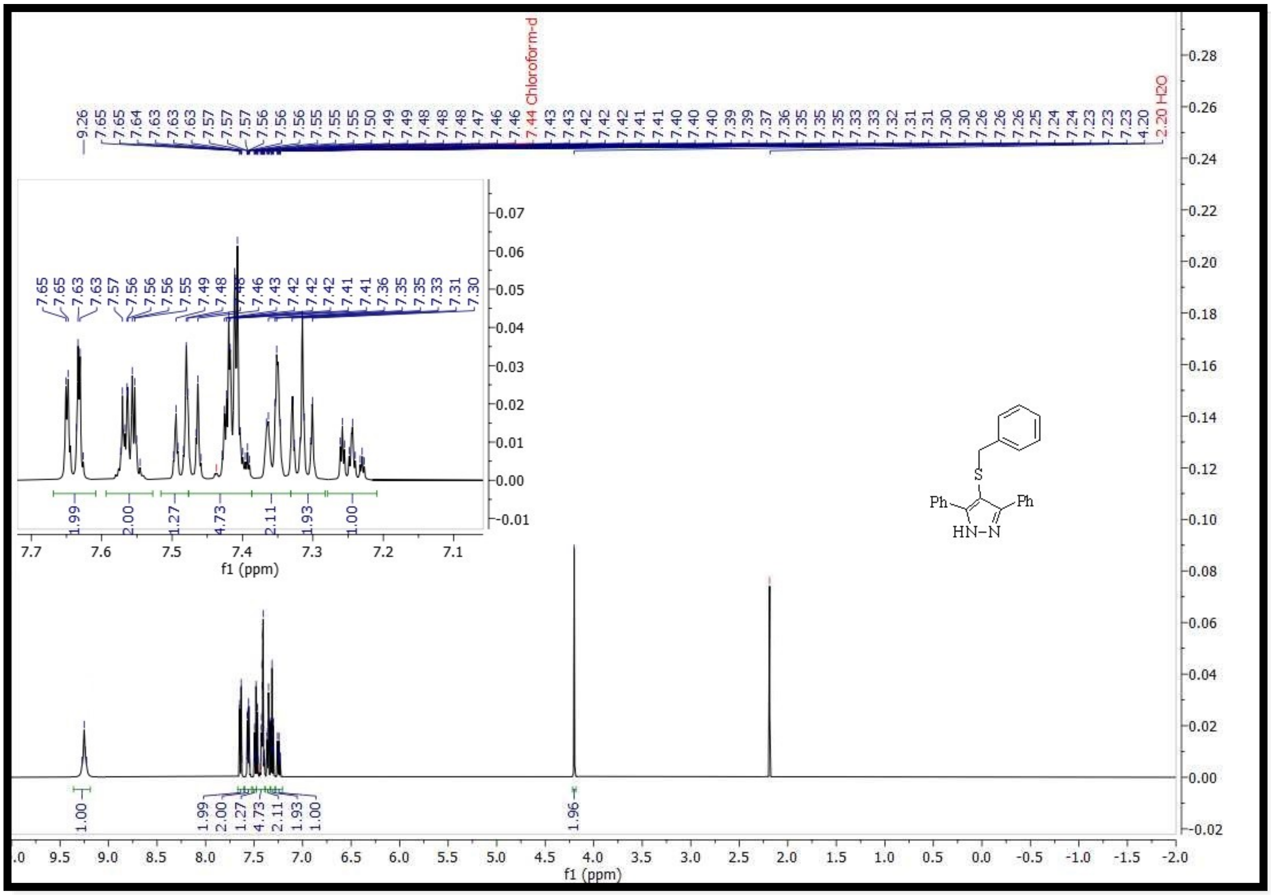

Figure 45: ^{1}H-NMR spectrum of compound **4w**

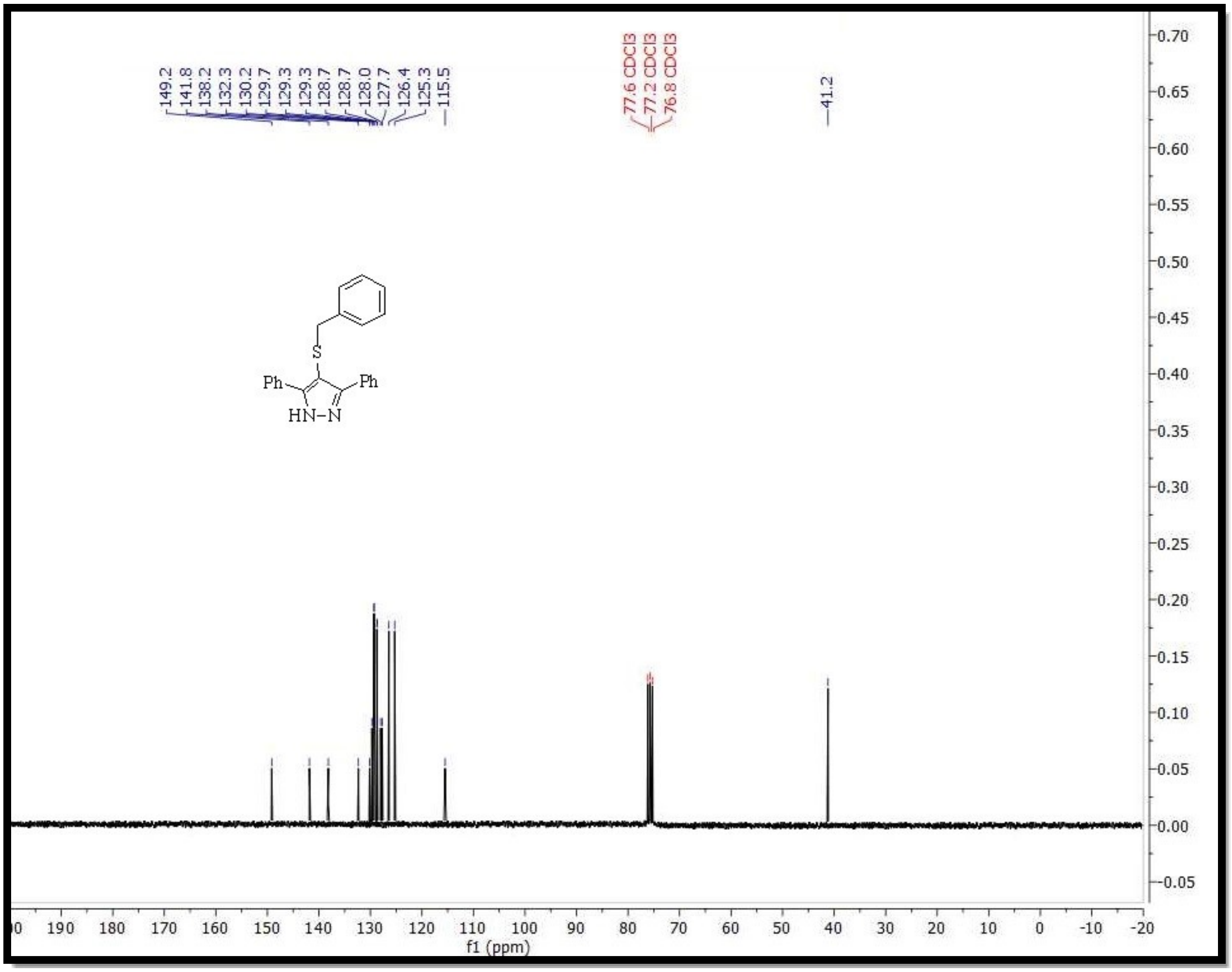

Figure 46: ^{13}C-NMR spectrum of compound **4w**

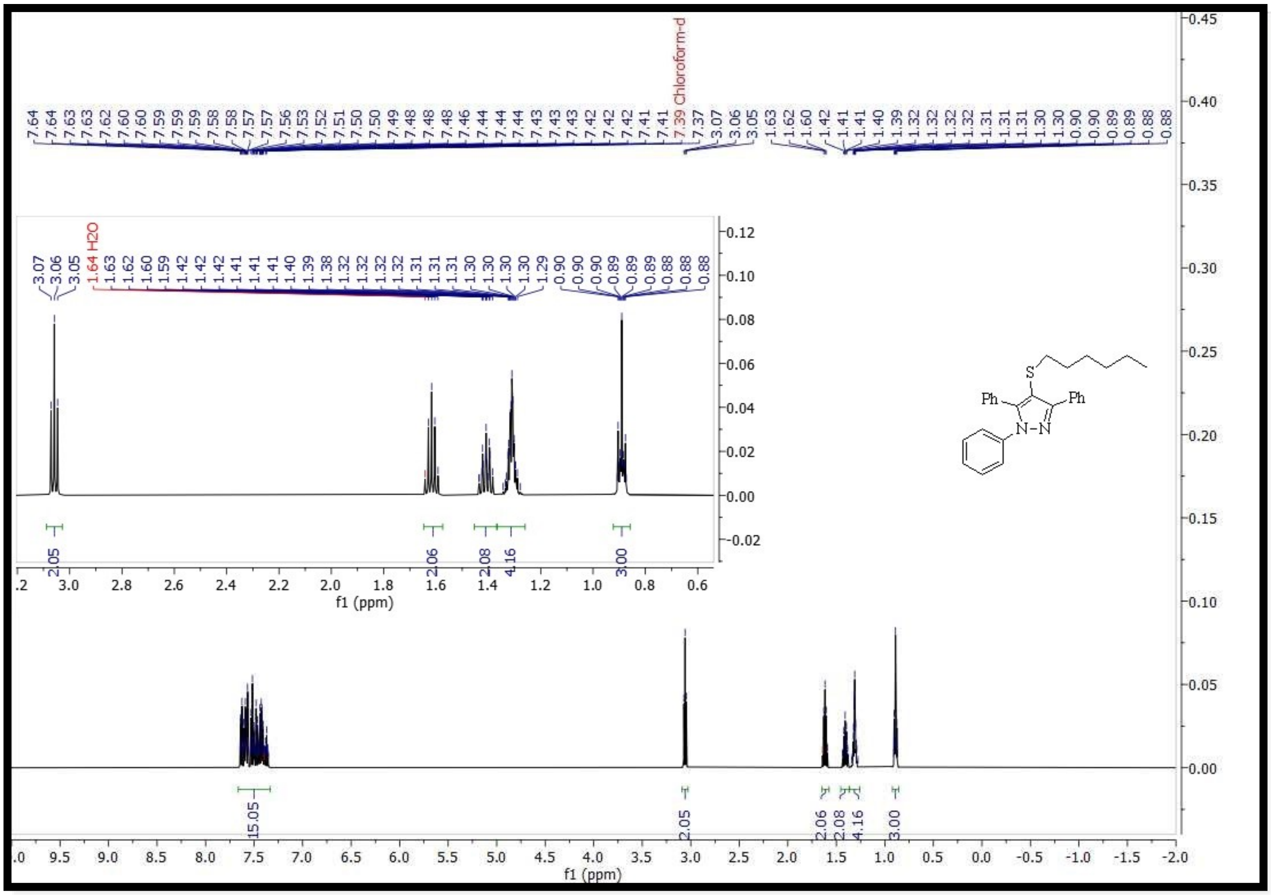

Figure 47: ^{1}H-NMR spectrum of compound **4x**

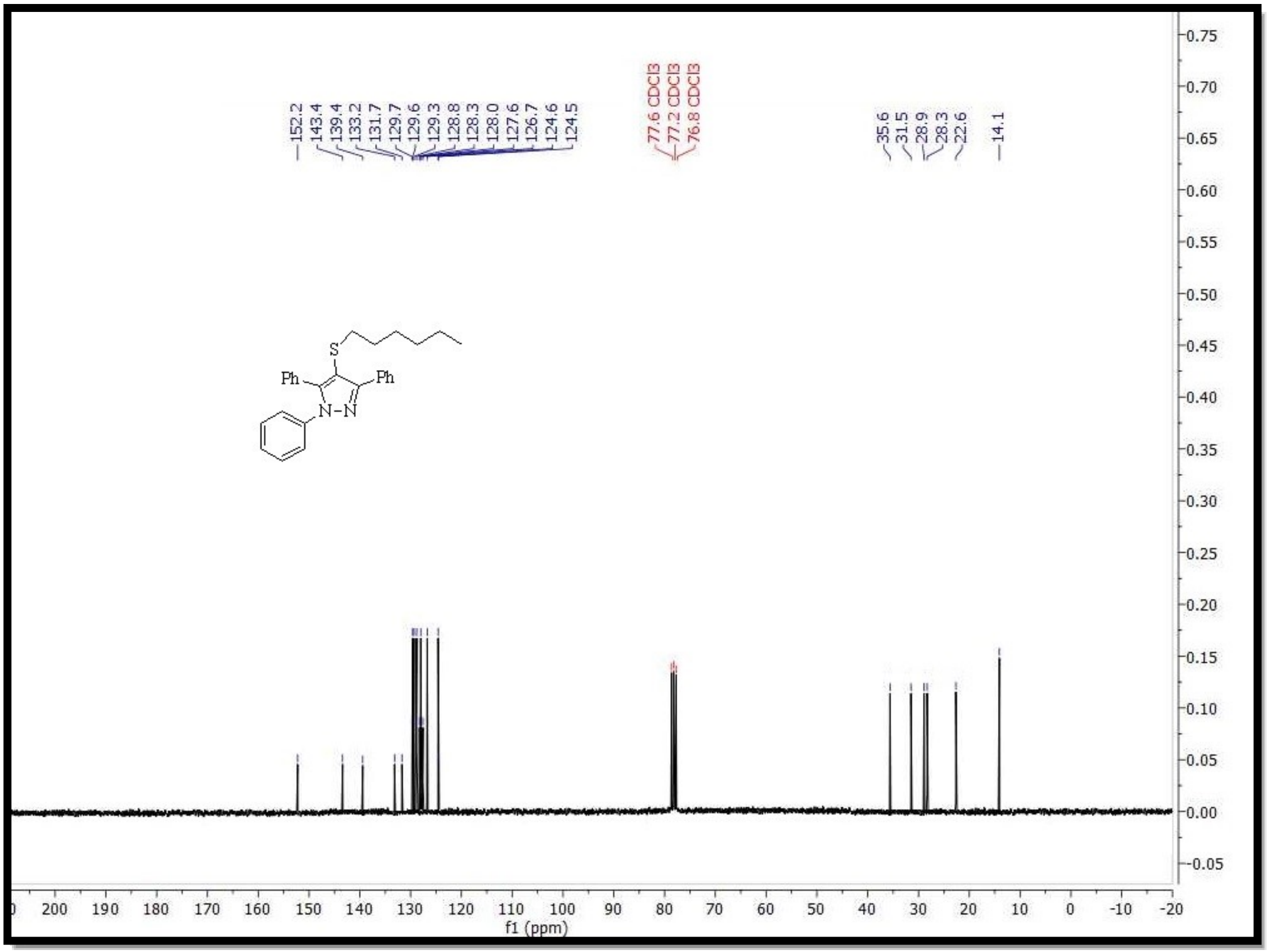

Figure 48: ^{13}C-NMR spectrum of compound **4x**

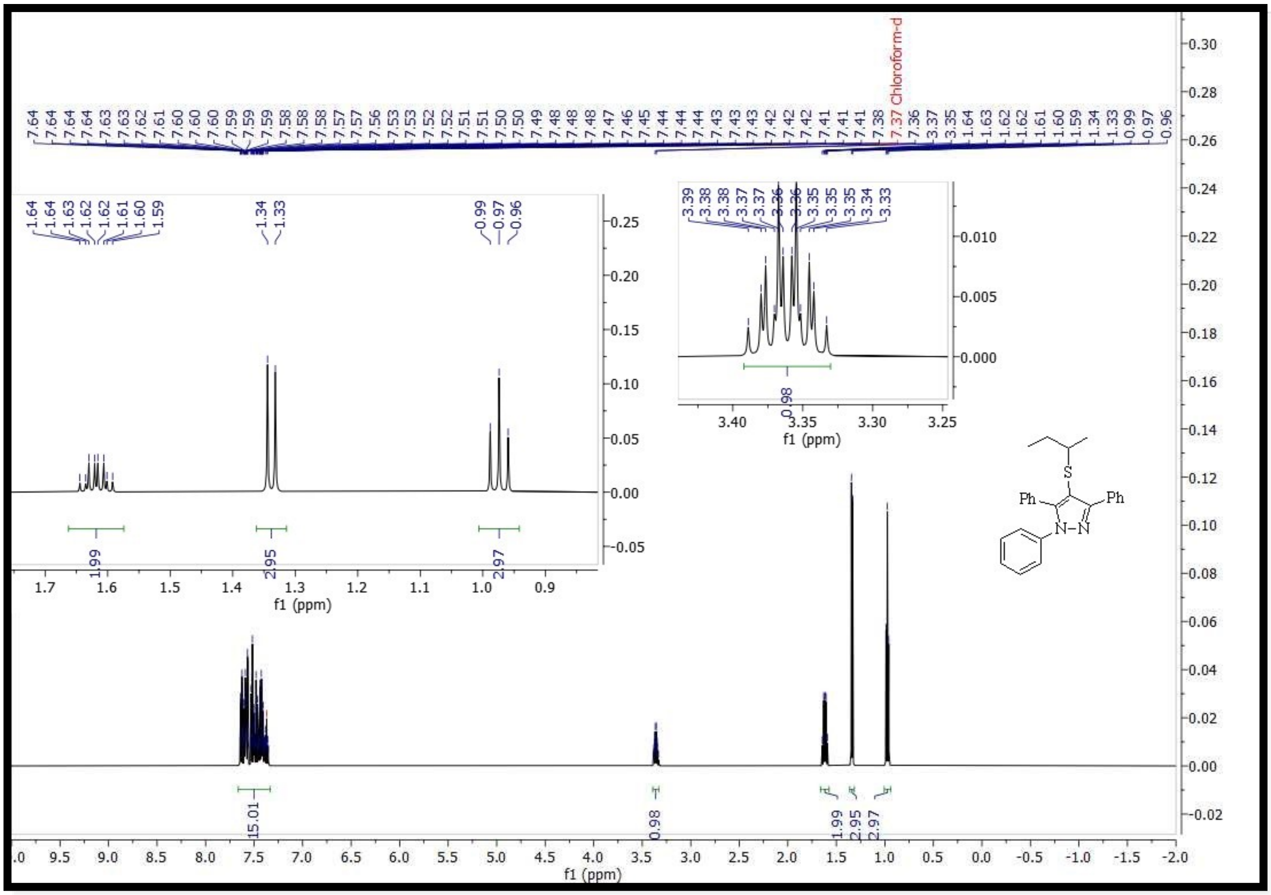

Figure 49: ^{1}H-NMR spectrum of compound **4y**

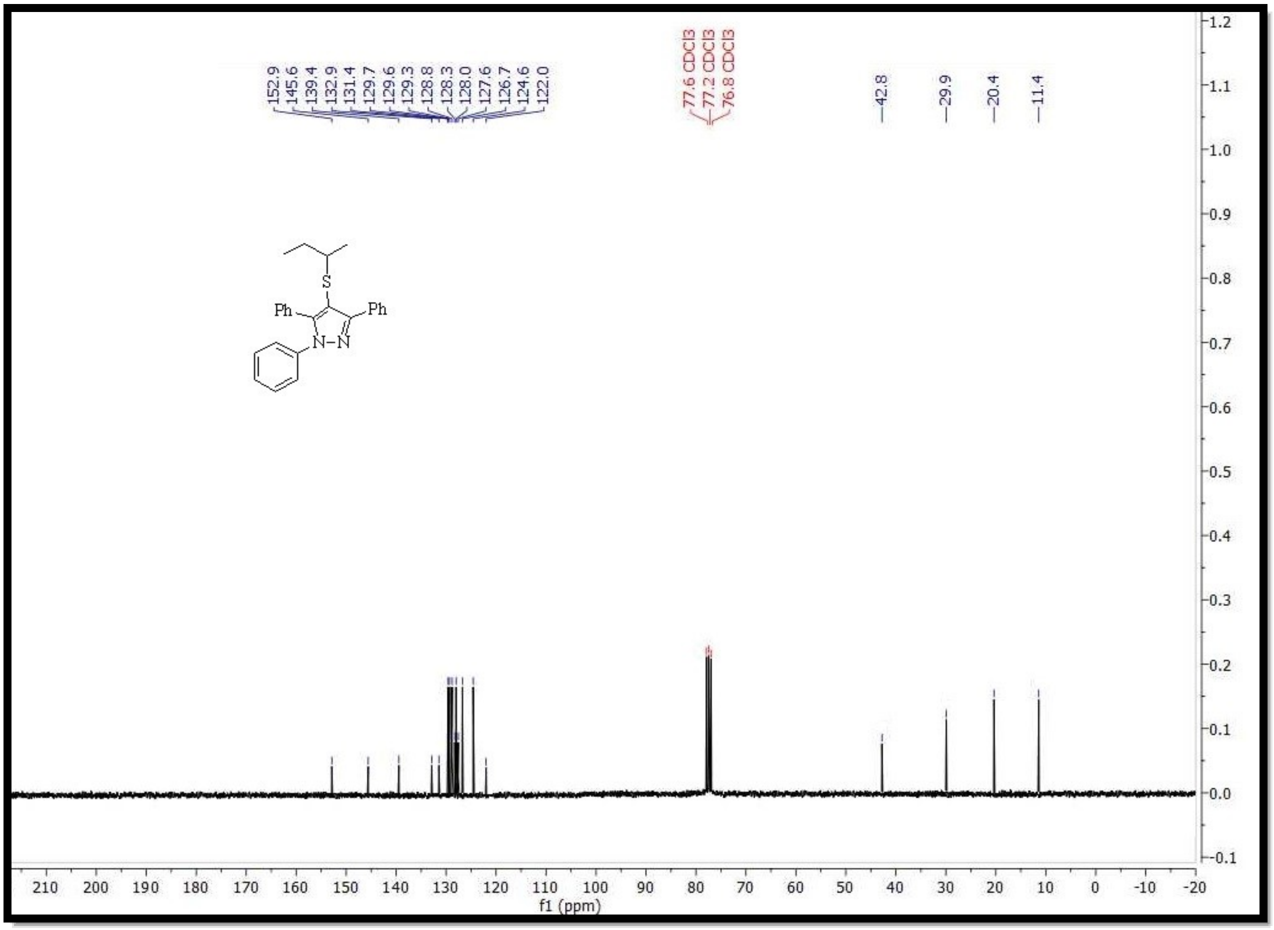

Figure 50: ^{13}C-NMR spectrum of compound **4y**

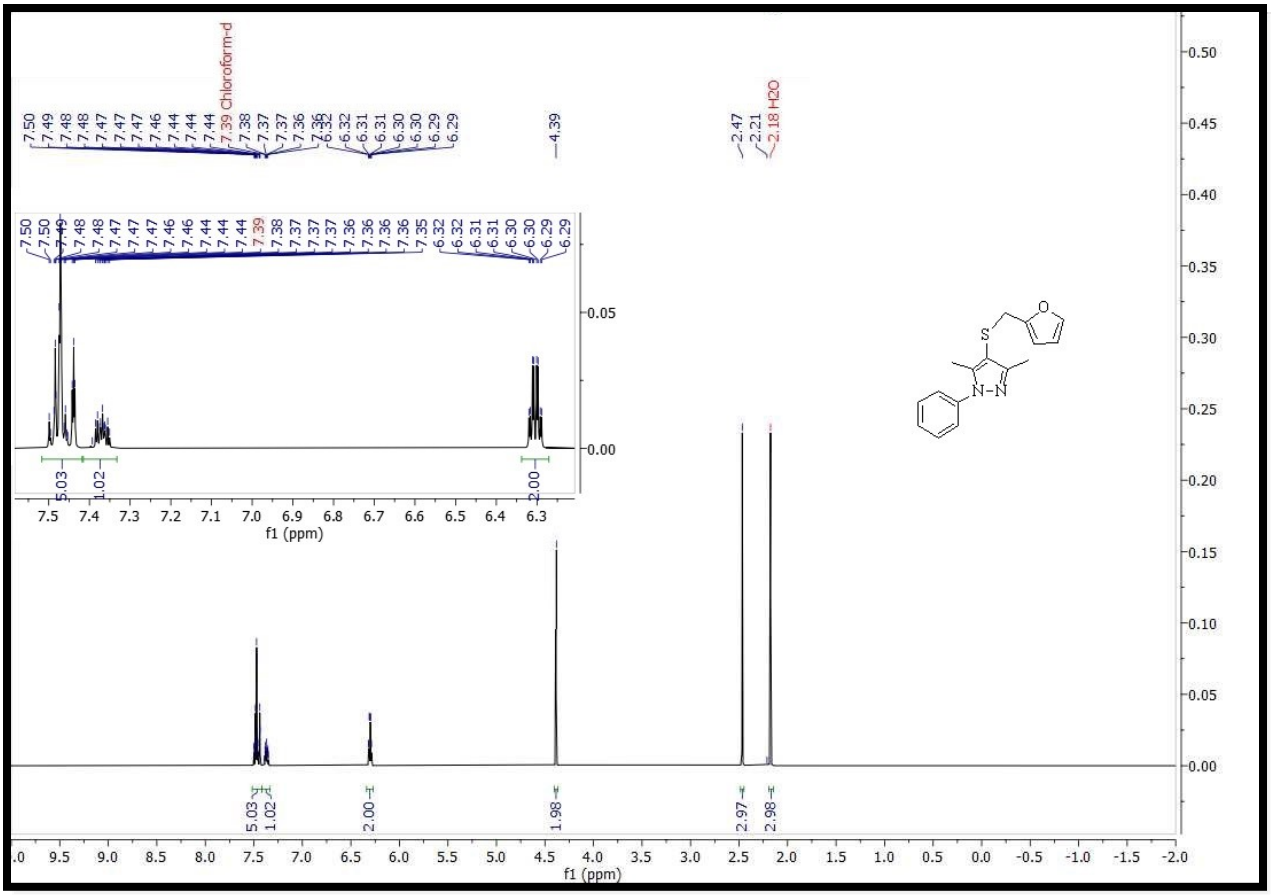

Figure 51: ^{1}H-NMR spectrum of compound **4z**

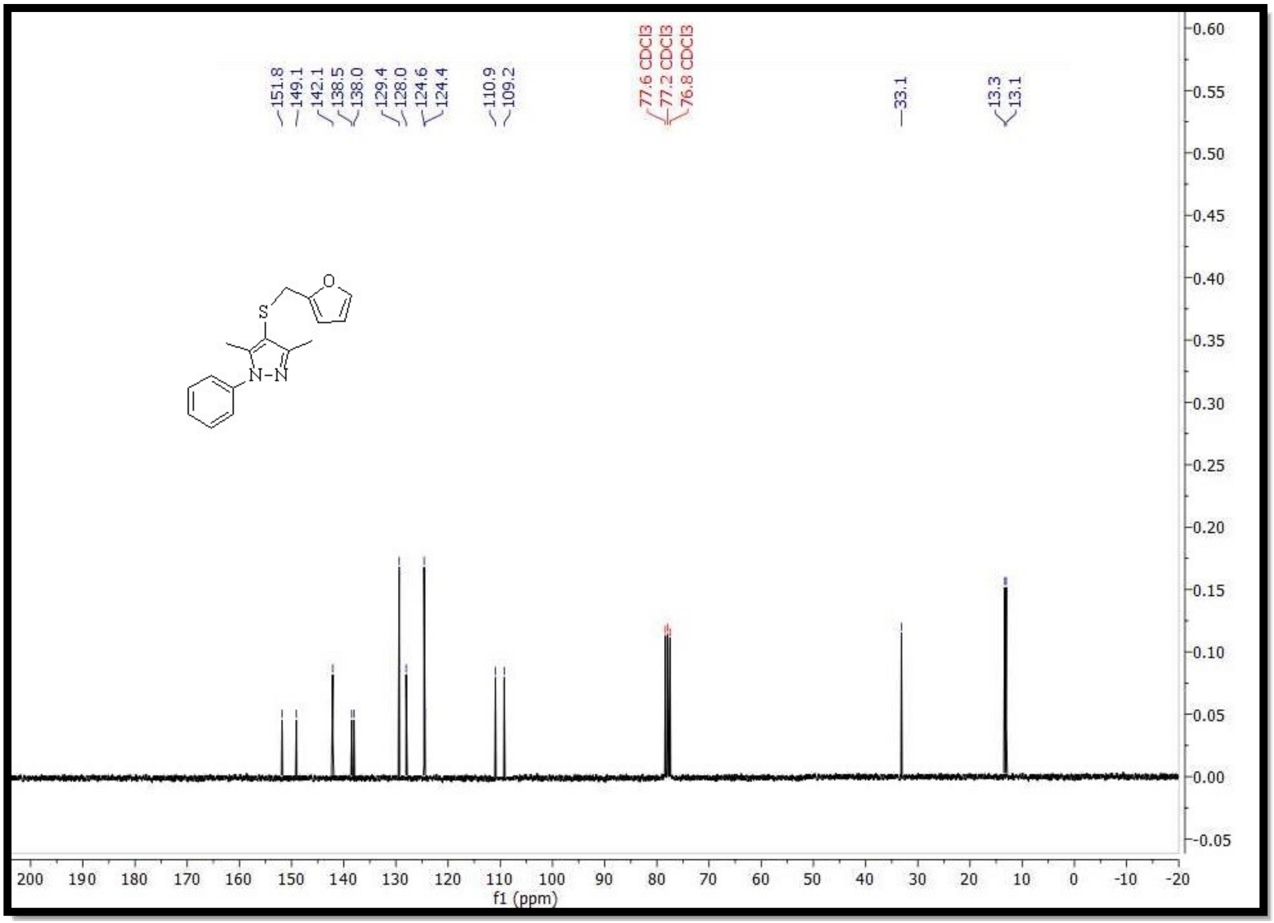

Figure 52: ^{13}C-NMR spectrum of compound **4z**

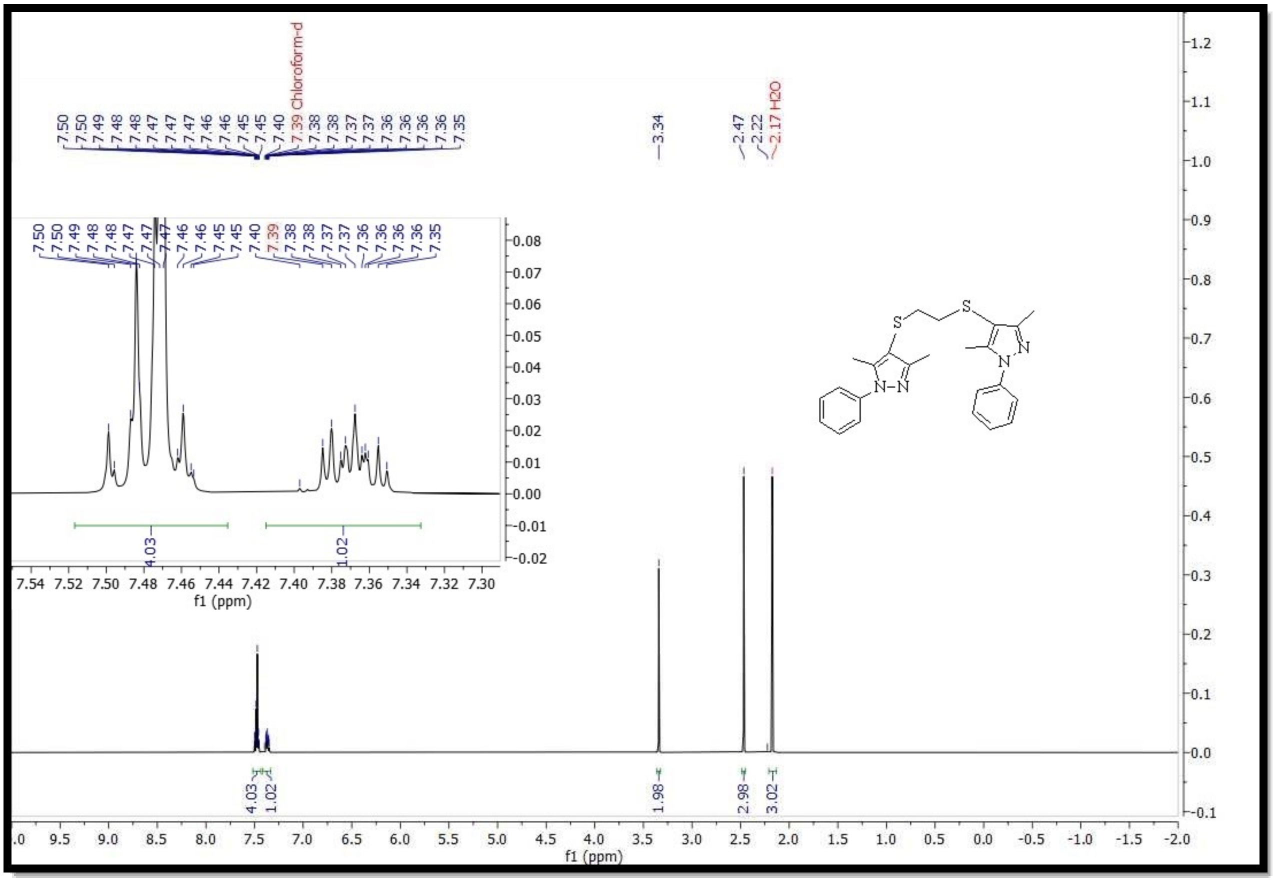

Figure 53: ^{1}H-NMR spectrum of compound **4aa**

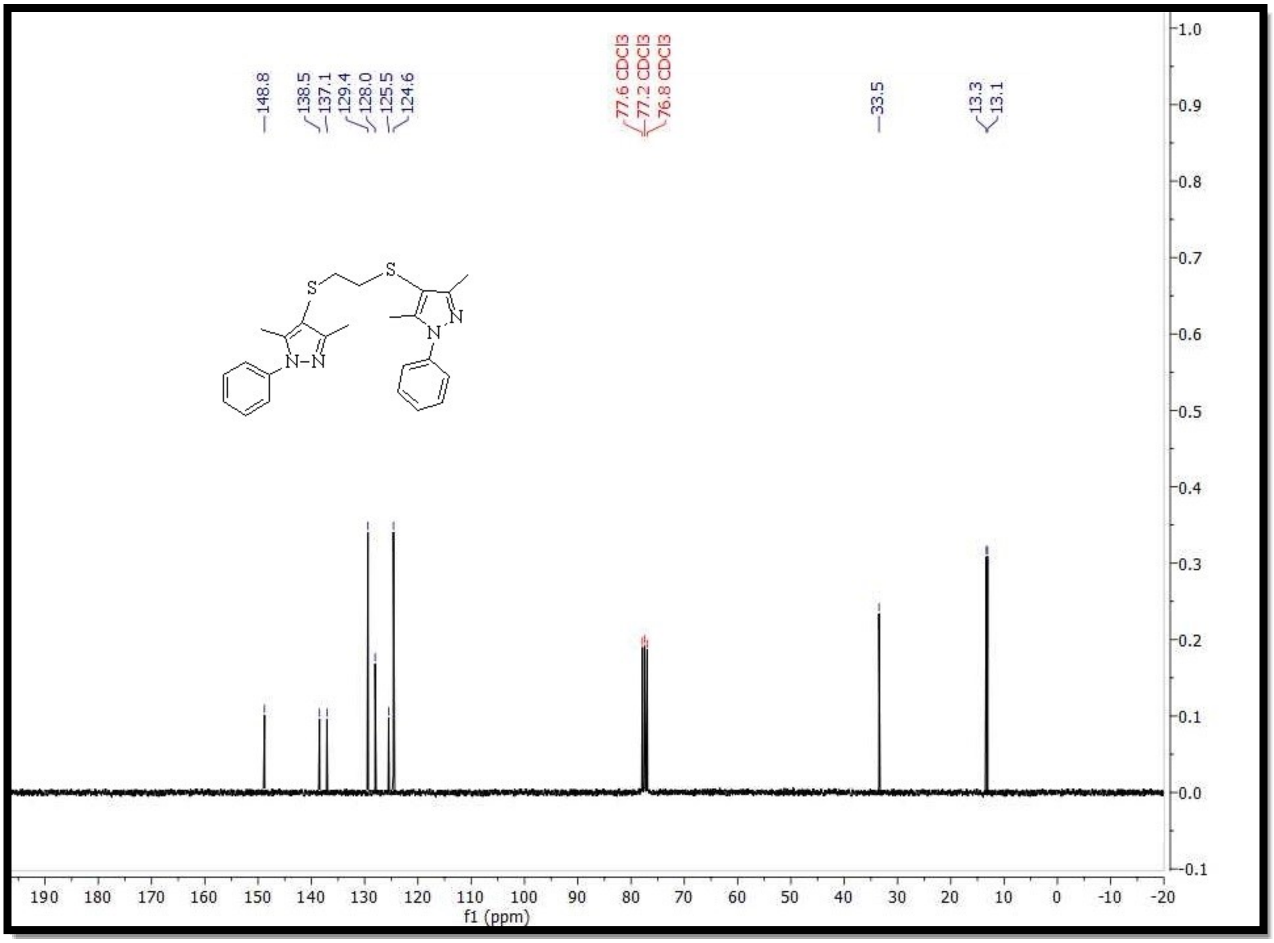

Figure 54: ^{13}C-NMR spectrum of compound **4aa**

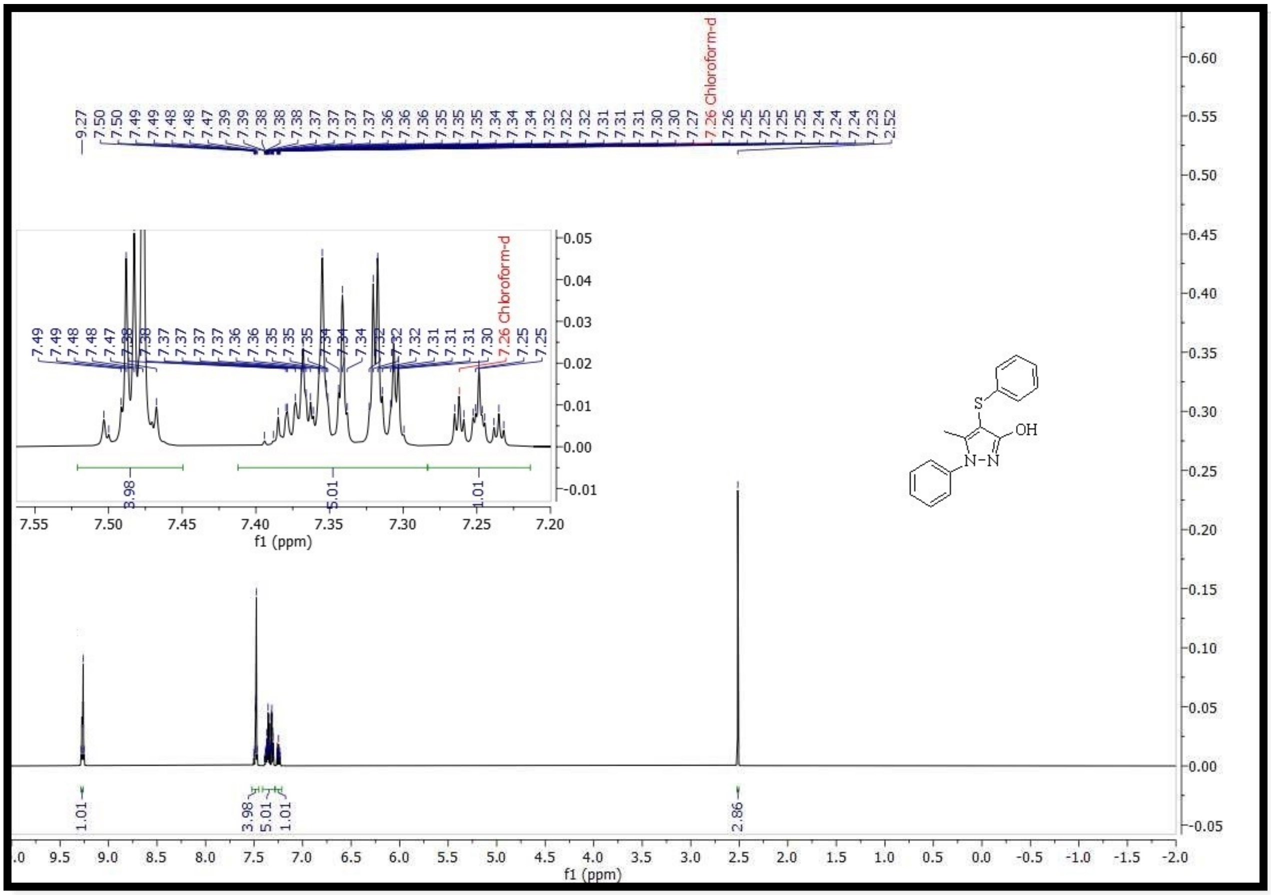

Figure 55: ^{1}H-NMR spectrum of compound **6a**

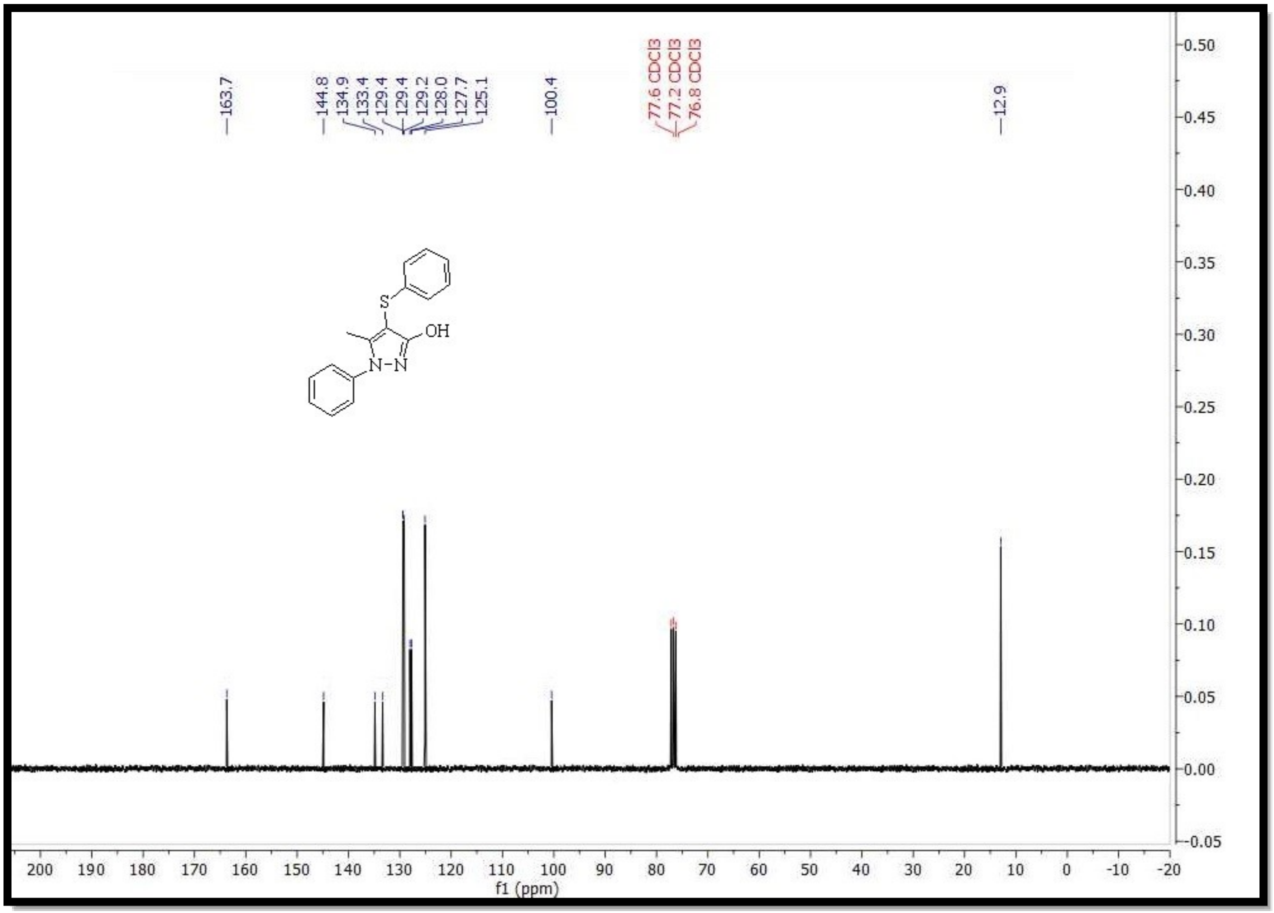

Figure 56: ^{13}C-NMR spectrum of compound **6a**

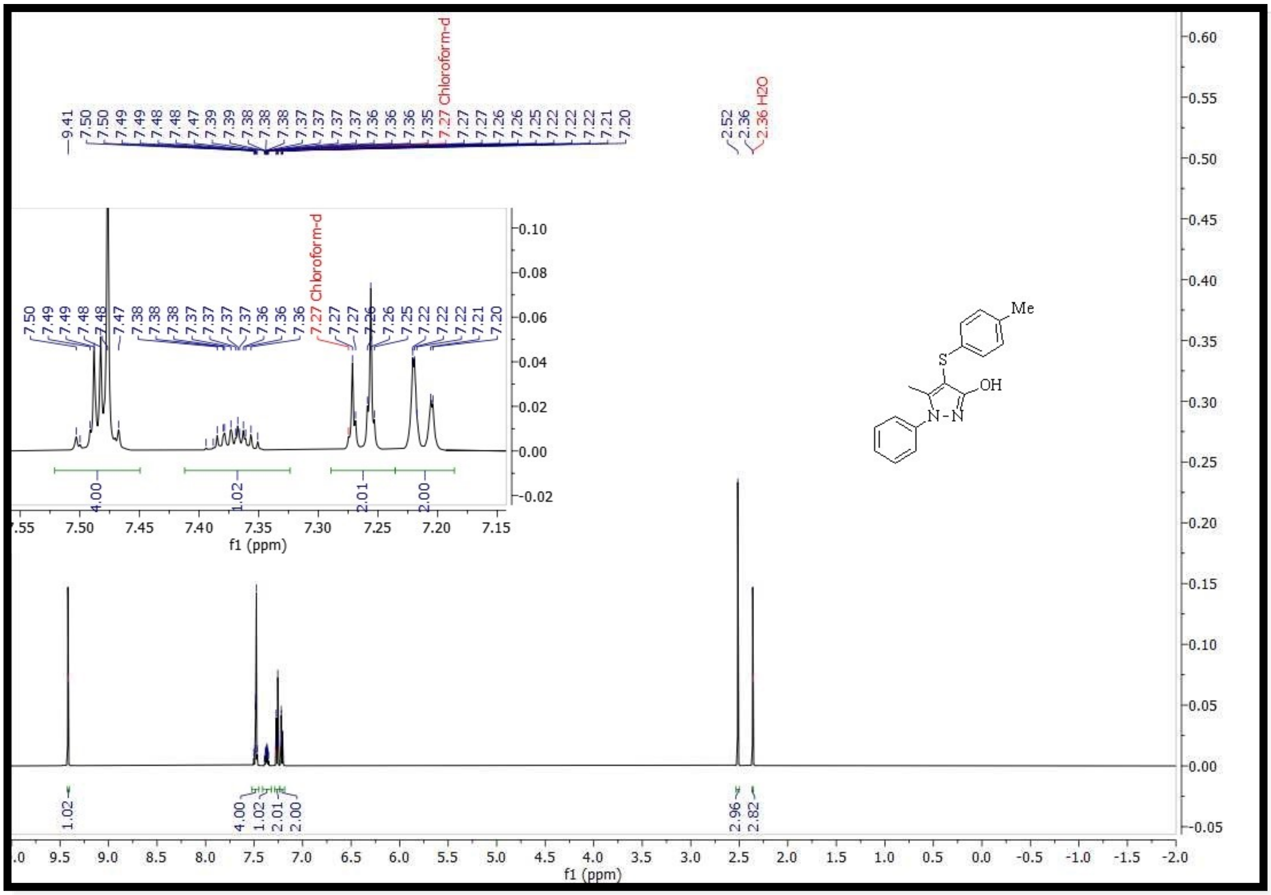

Figure 57: ^{1}H-NMR spectrum of compound **6b**

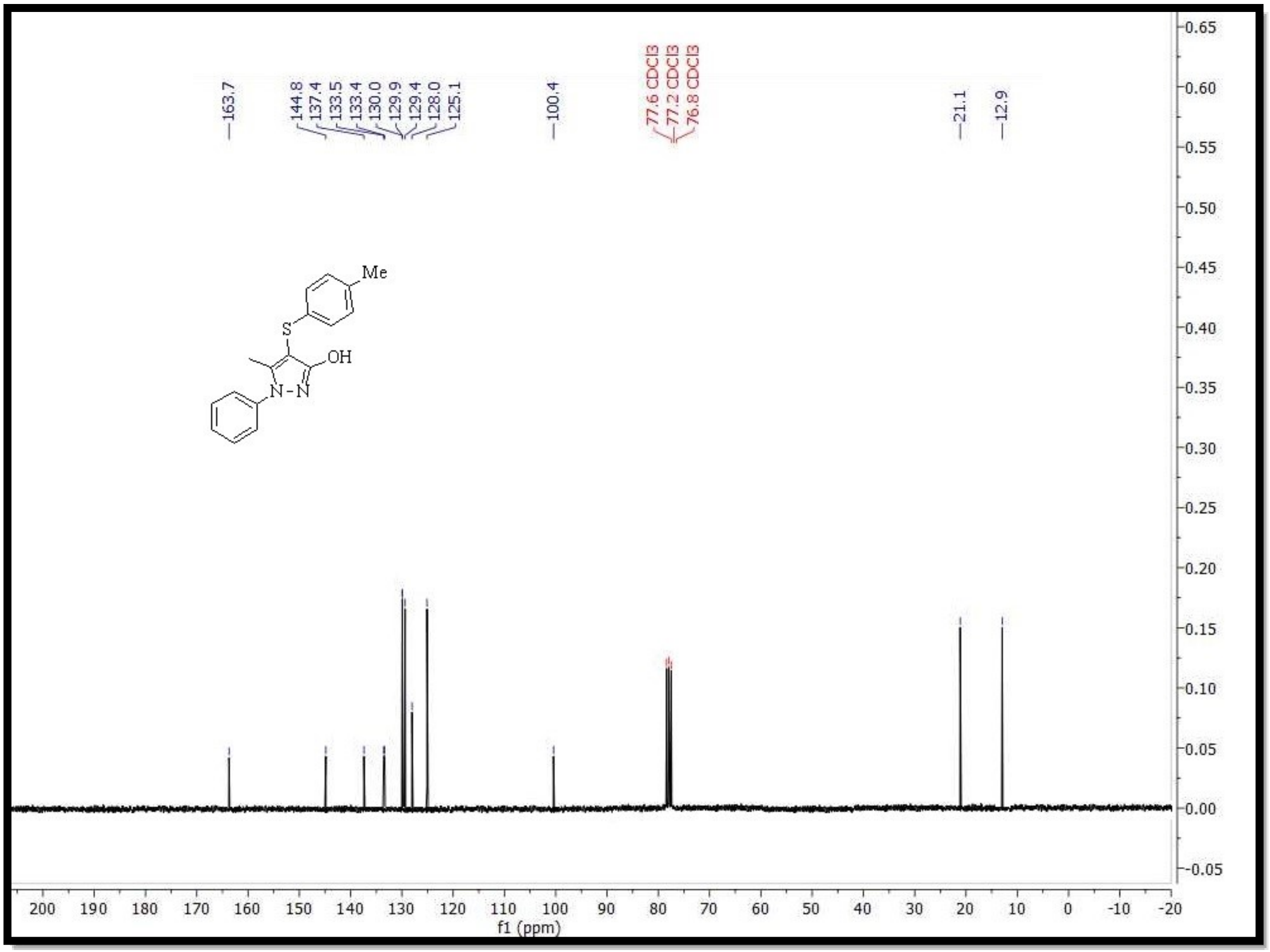

Figure 58: ^{13}C-NMR spectrum of compound **6b**

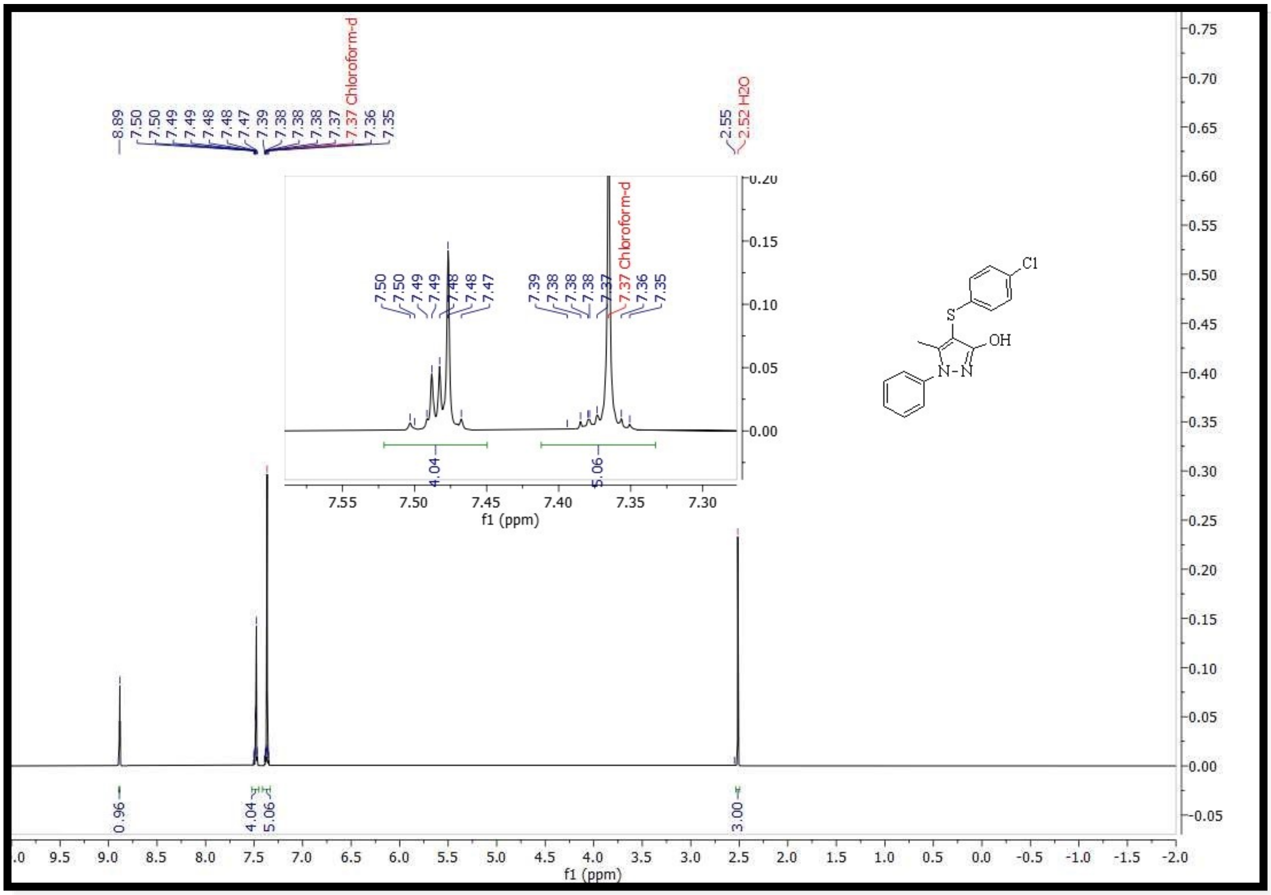

Figure 59: ^{1}H-NMR spectrum of compound **6c**

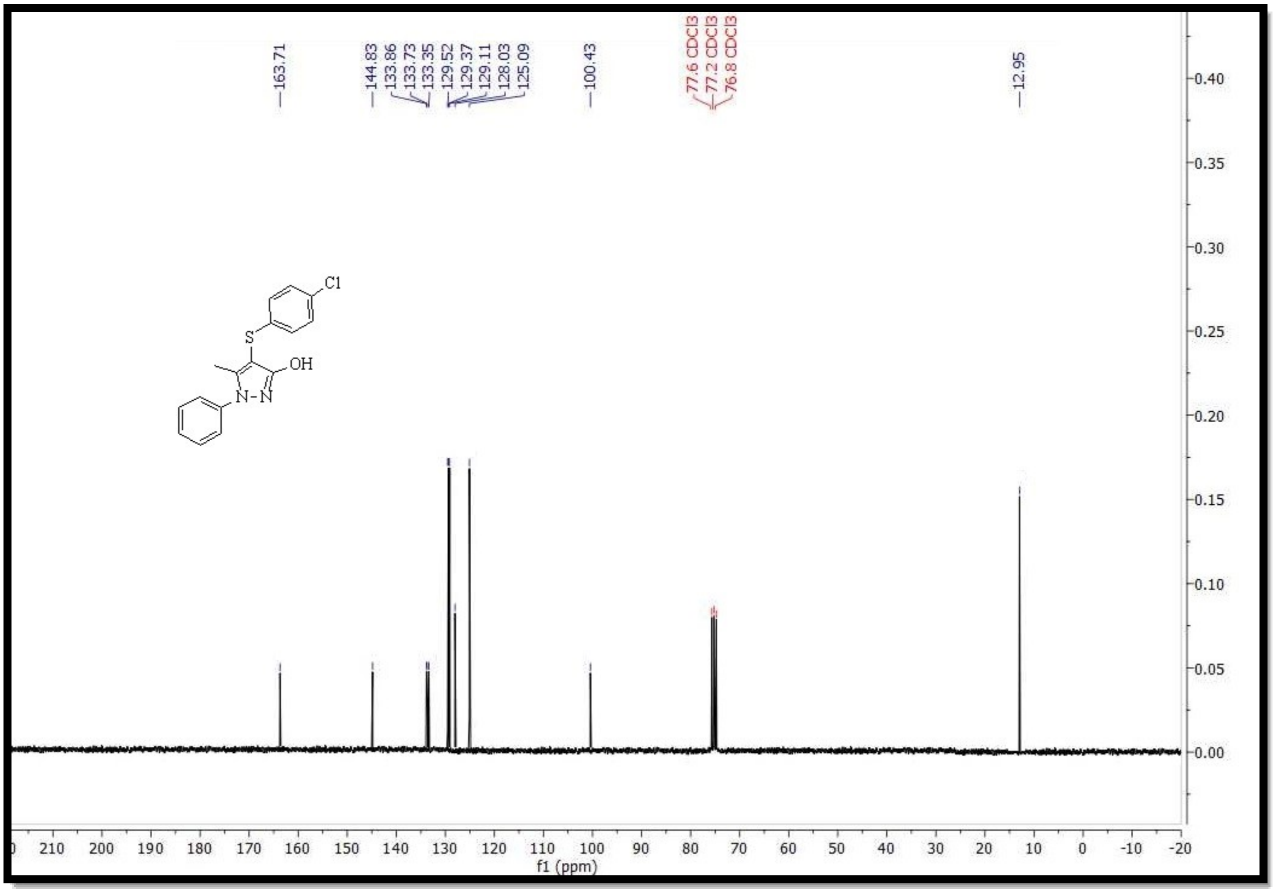

Figure 60: ^{13}C-NMR spectrum of compound **6c**

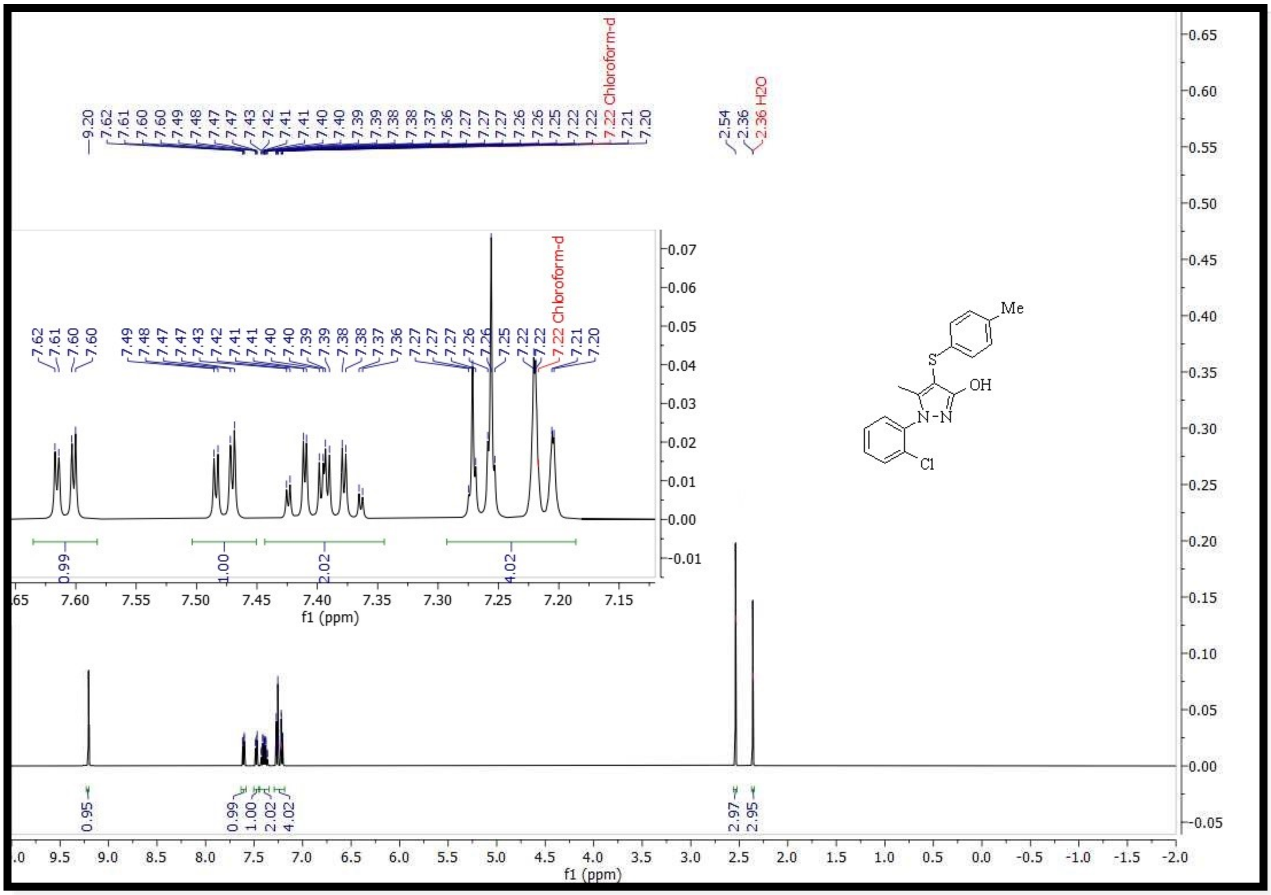

Figure 61: ^{1}H-NMR spectrum of compound **6d**

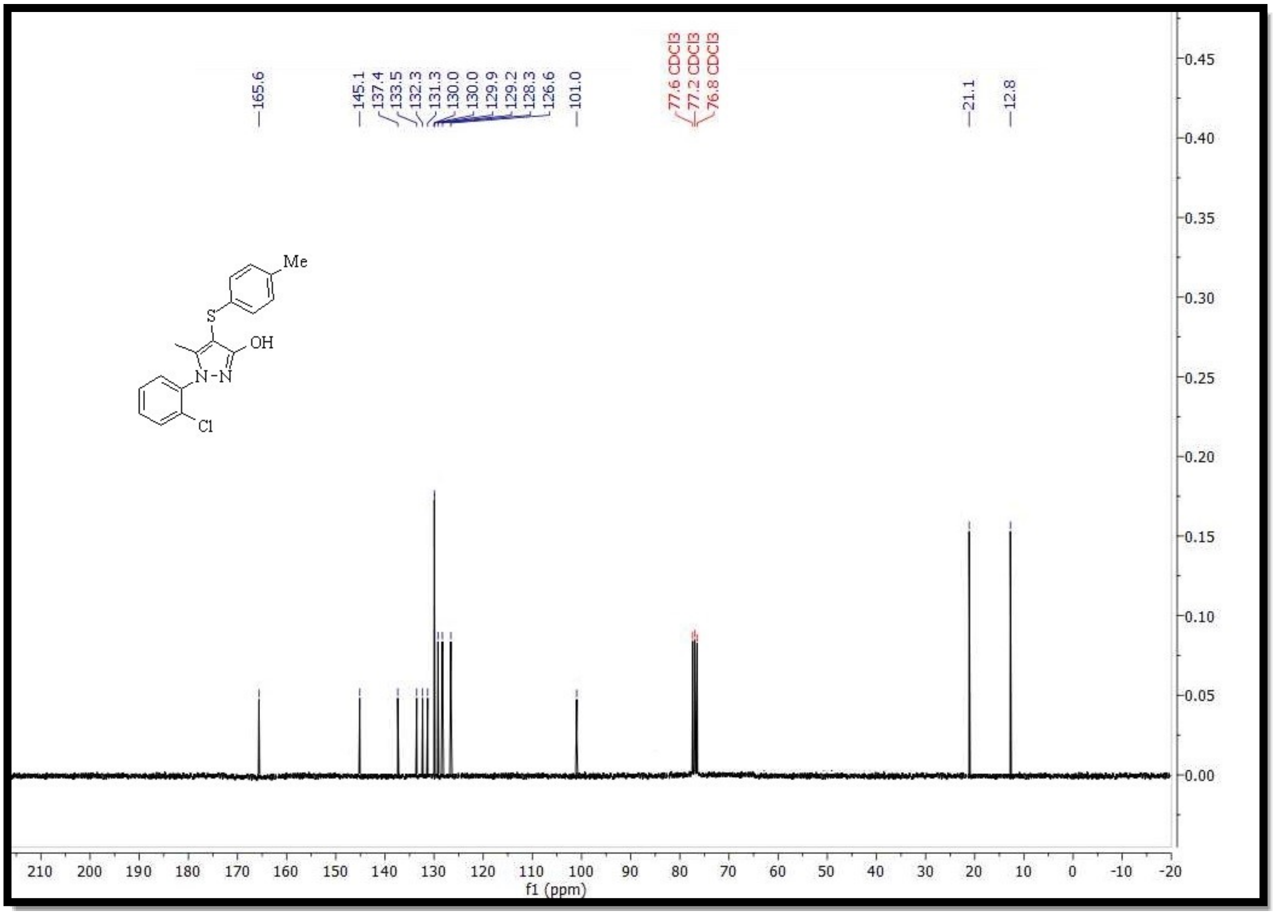

Figure 62: ^{13}C-NMR spectrum of compound **6d**

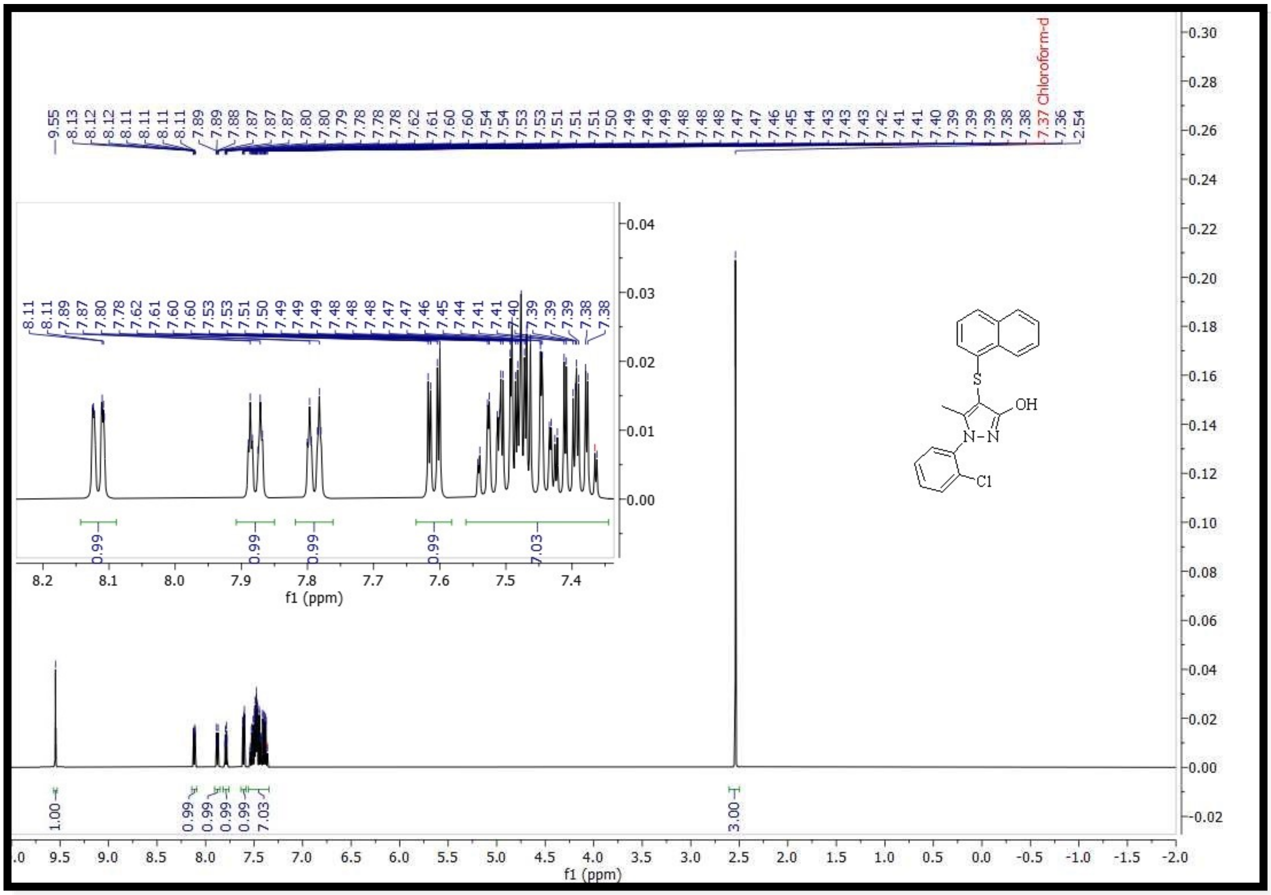

Figure 63: ^{1}H-NMR spectrum of compound **6e**

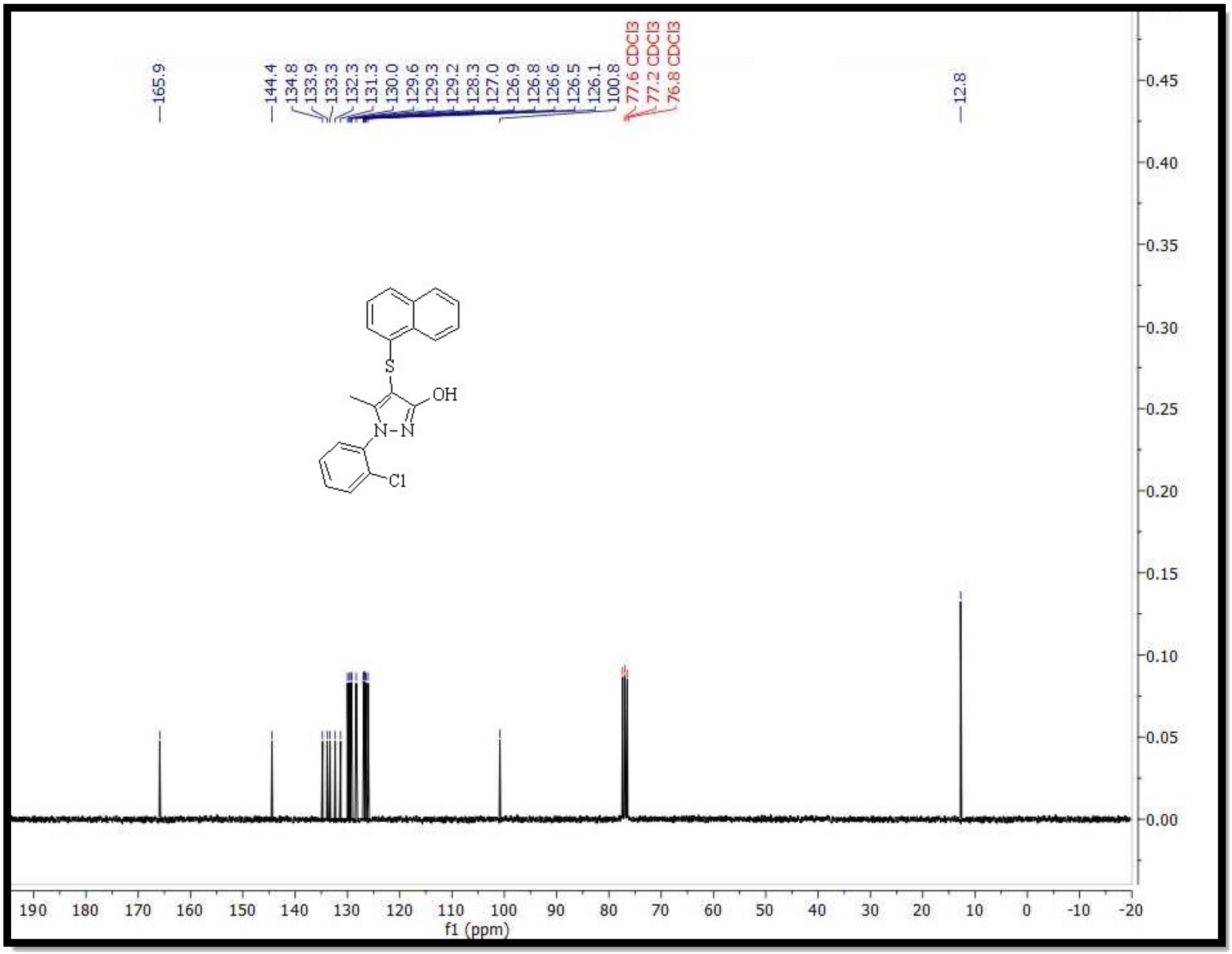

Figure 64: ^{13}C-NMR spectrum of compound **6e**

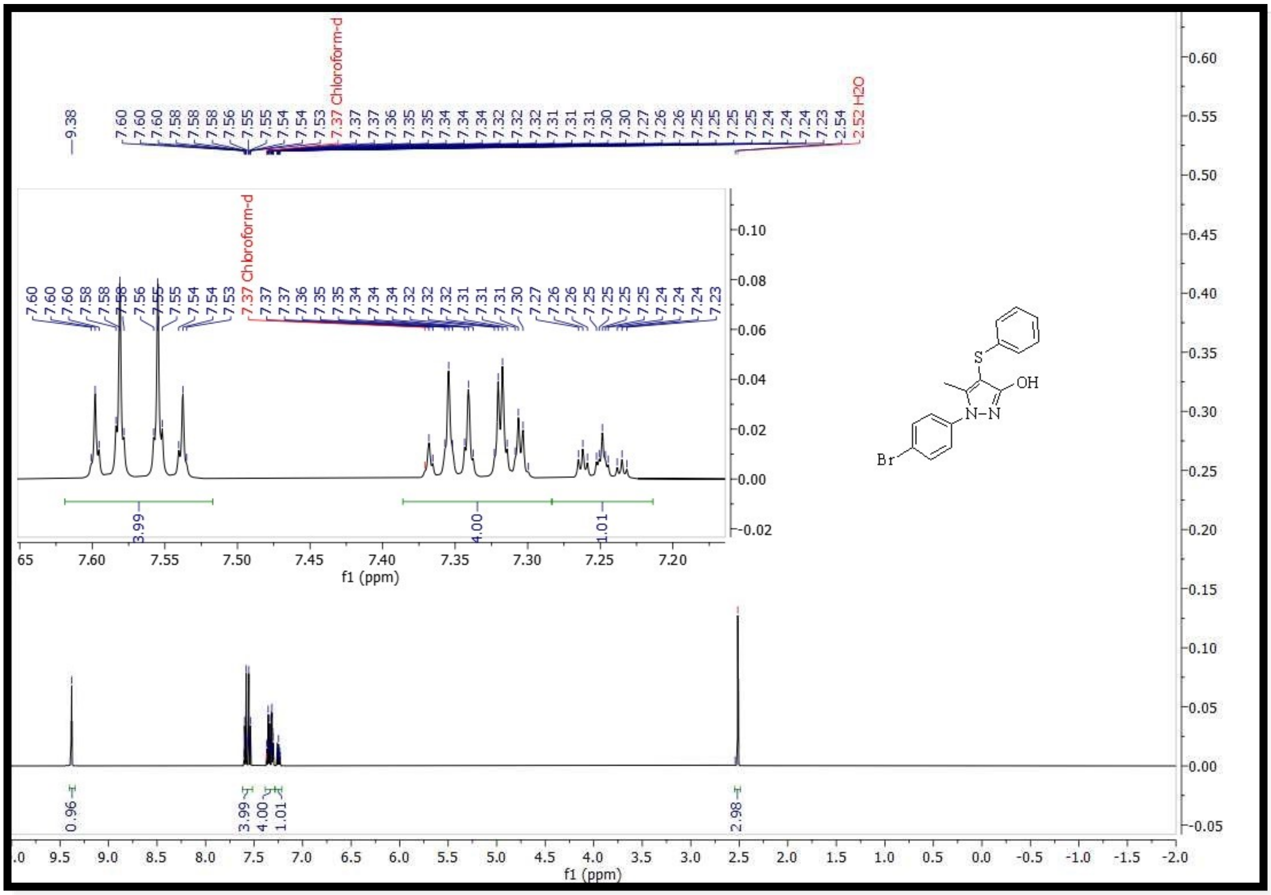

Figure 65: ^{1}H-NMR spectrum of compound **6f**

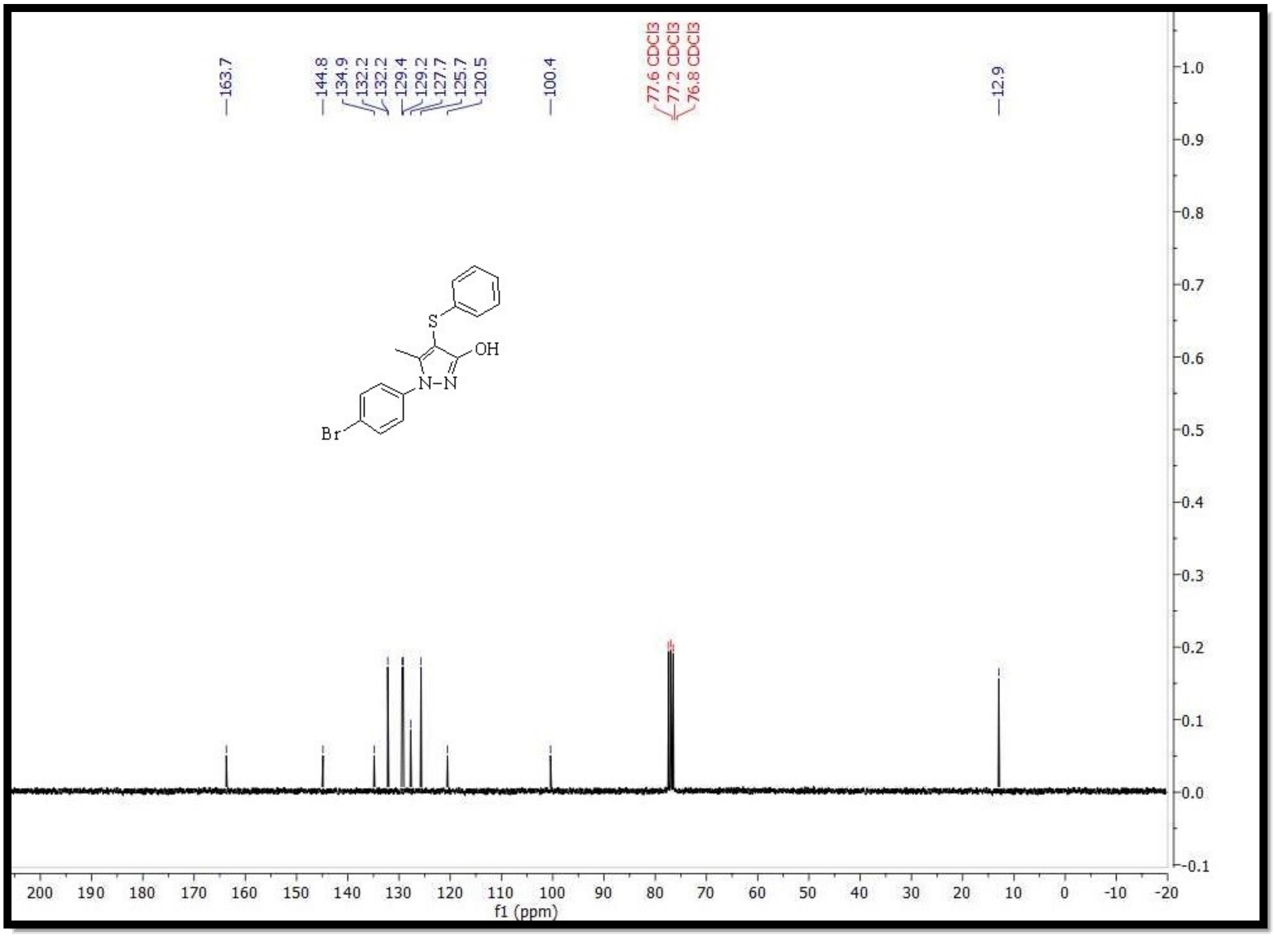

Figure 66: ^{13}C-NMR spectrum of compound **6f**

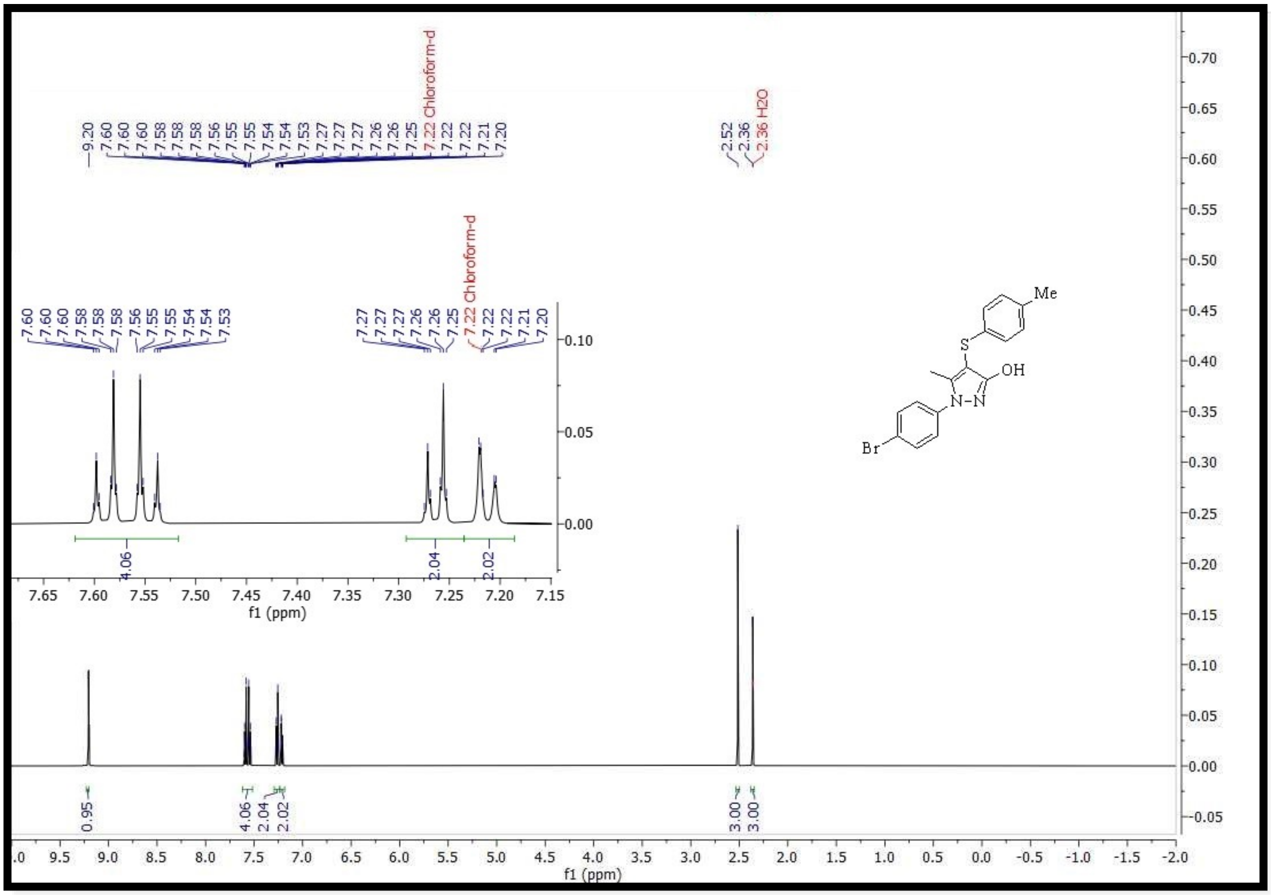

Figure 67: ^{1}H-NMR spectrum of compound **6g**

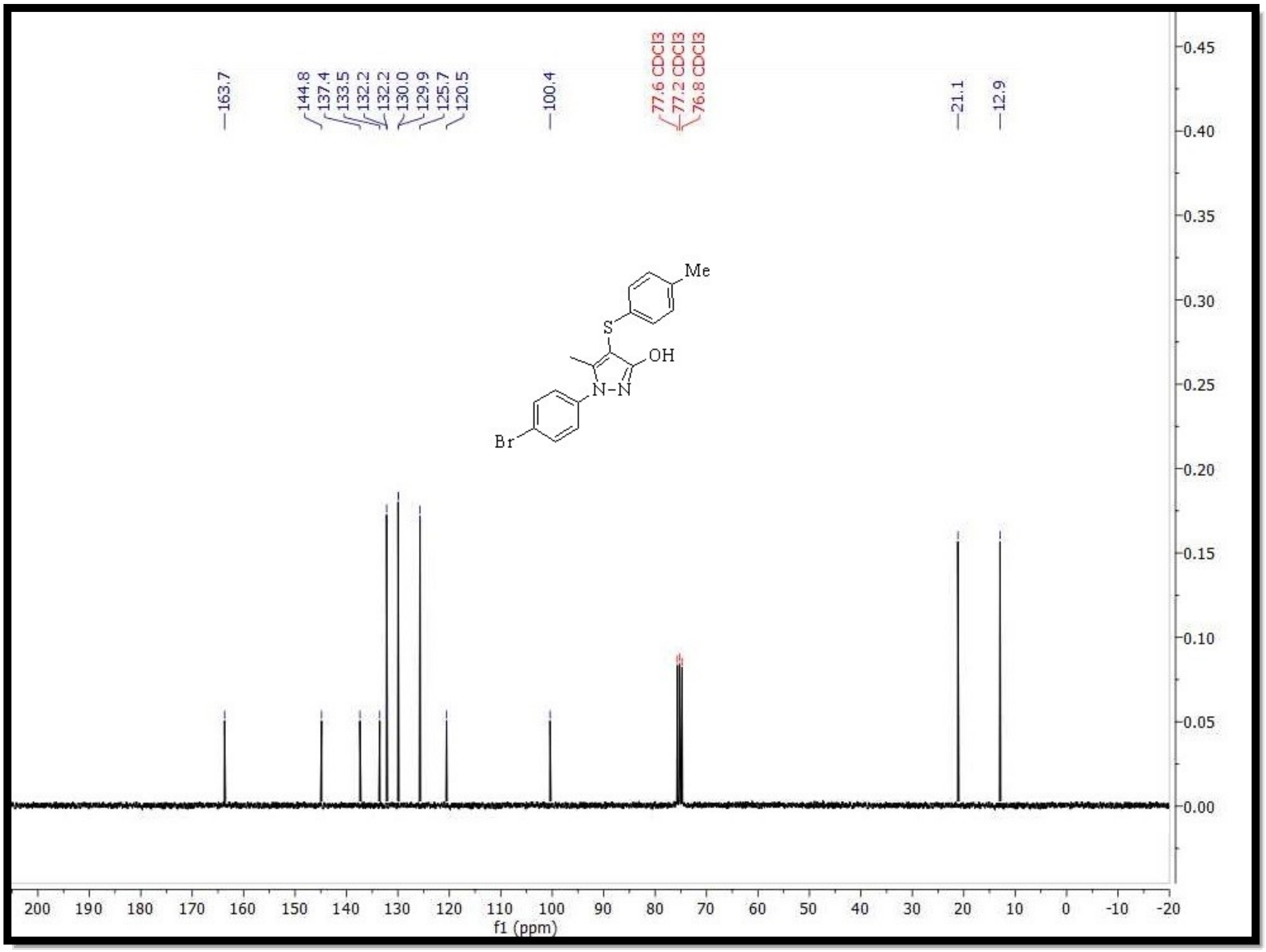

Figure 68: ^{13}C-NMR spectrum of compound **6g**

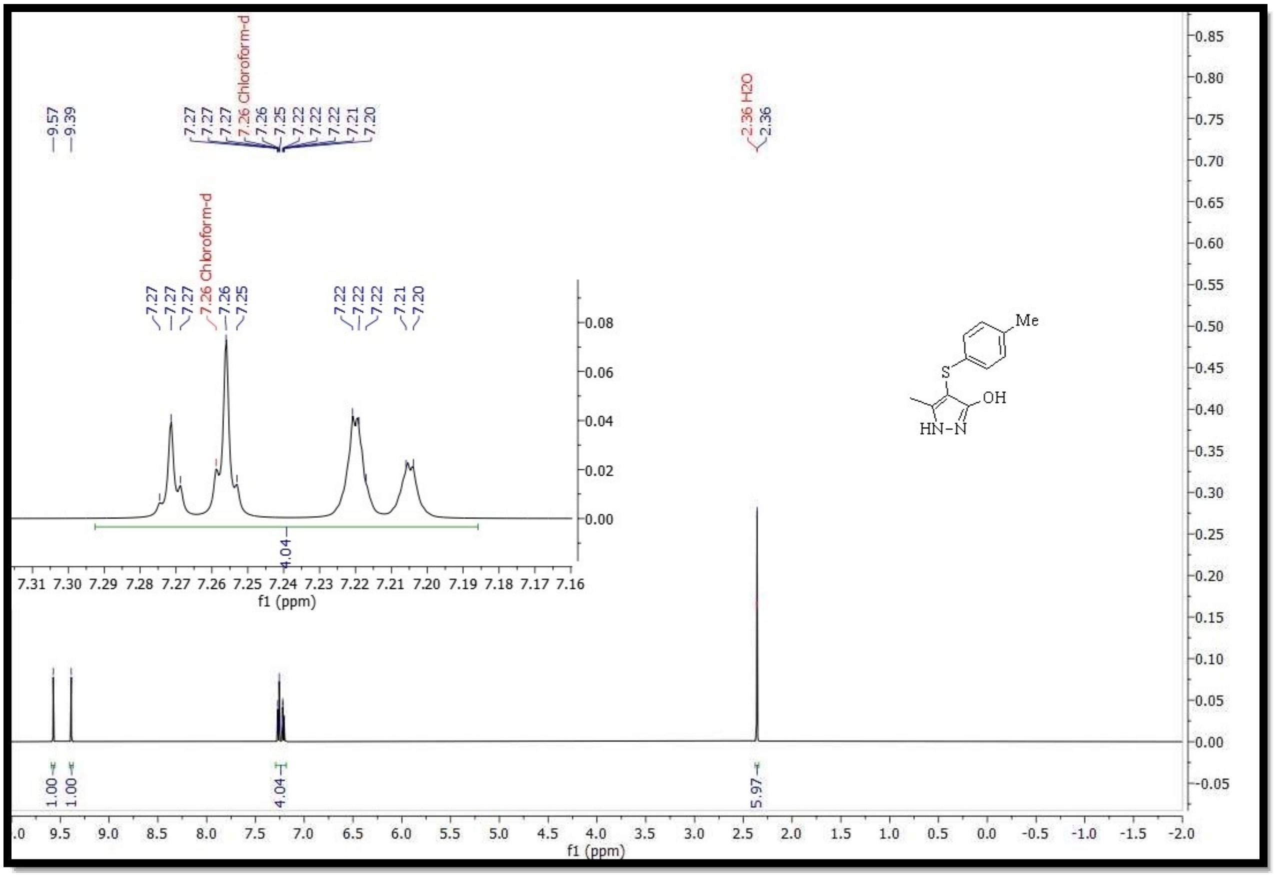

Figure 69: ^{1}H-NMR spectrum of compound **6h**

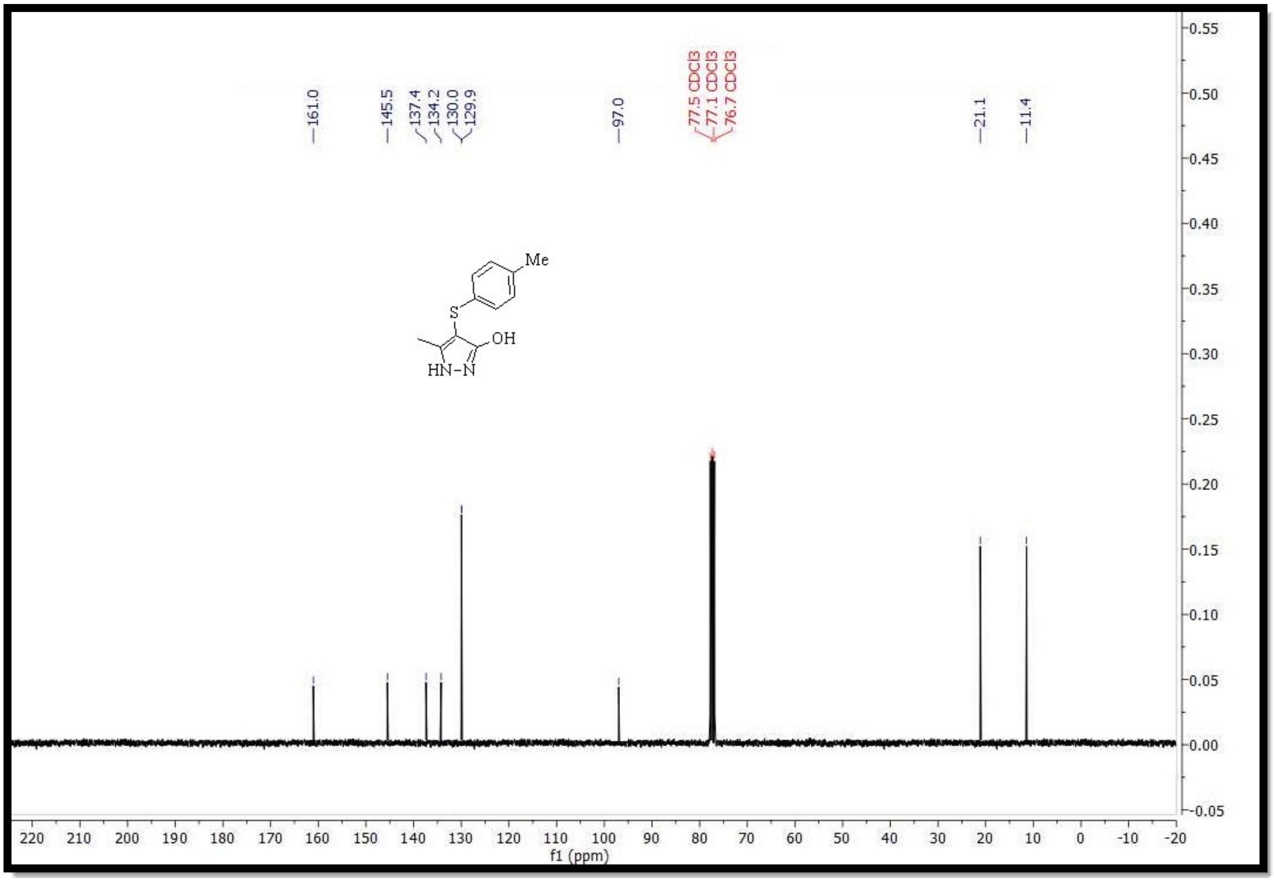

Figure 70: ^{13}C-NMR spectrum of compound **6h**

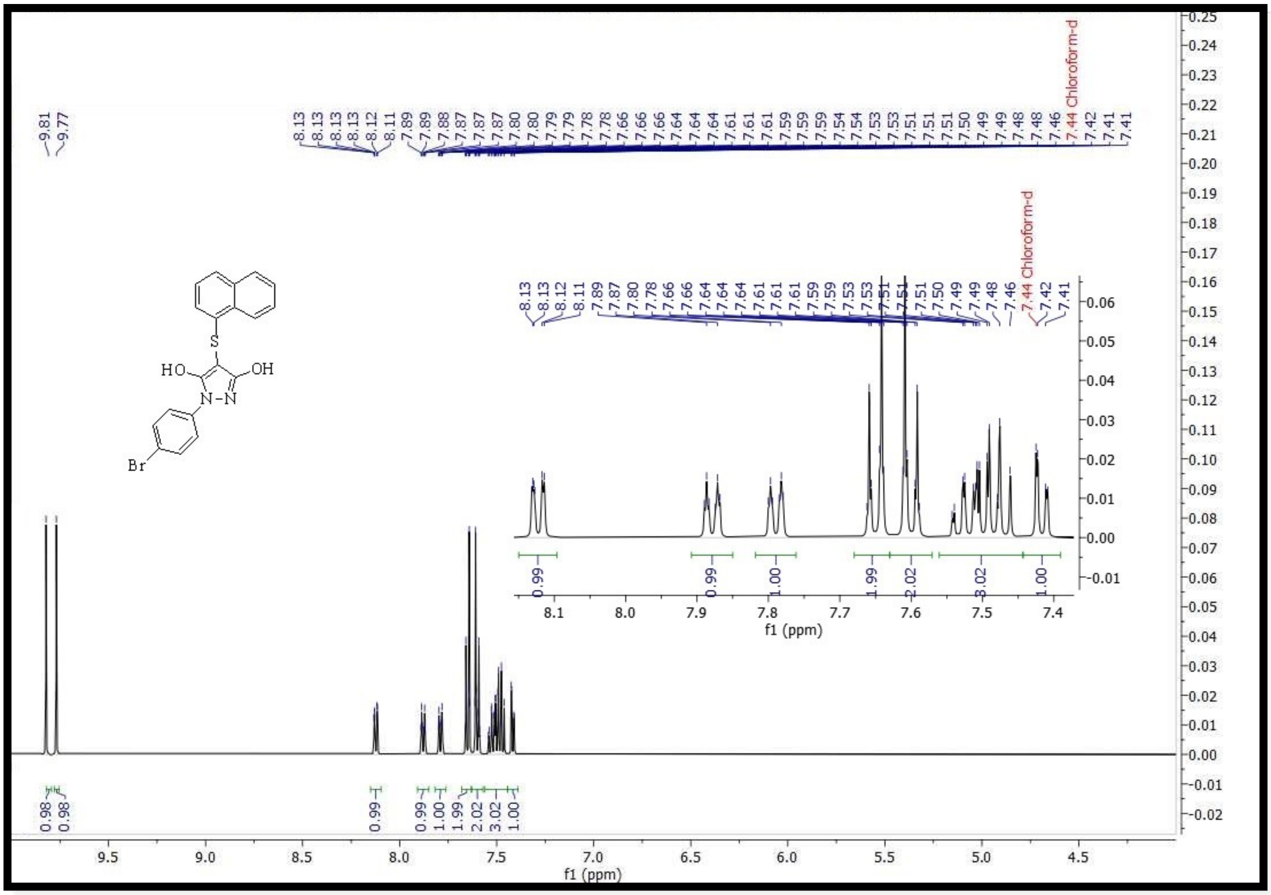

Figure 71: ^{1}H-NMR spectrum of compound **6i**

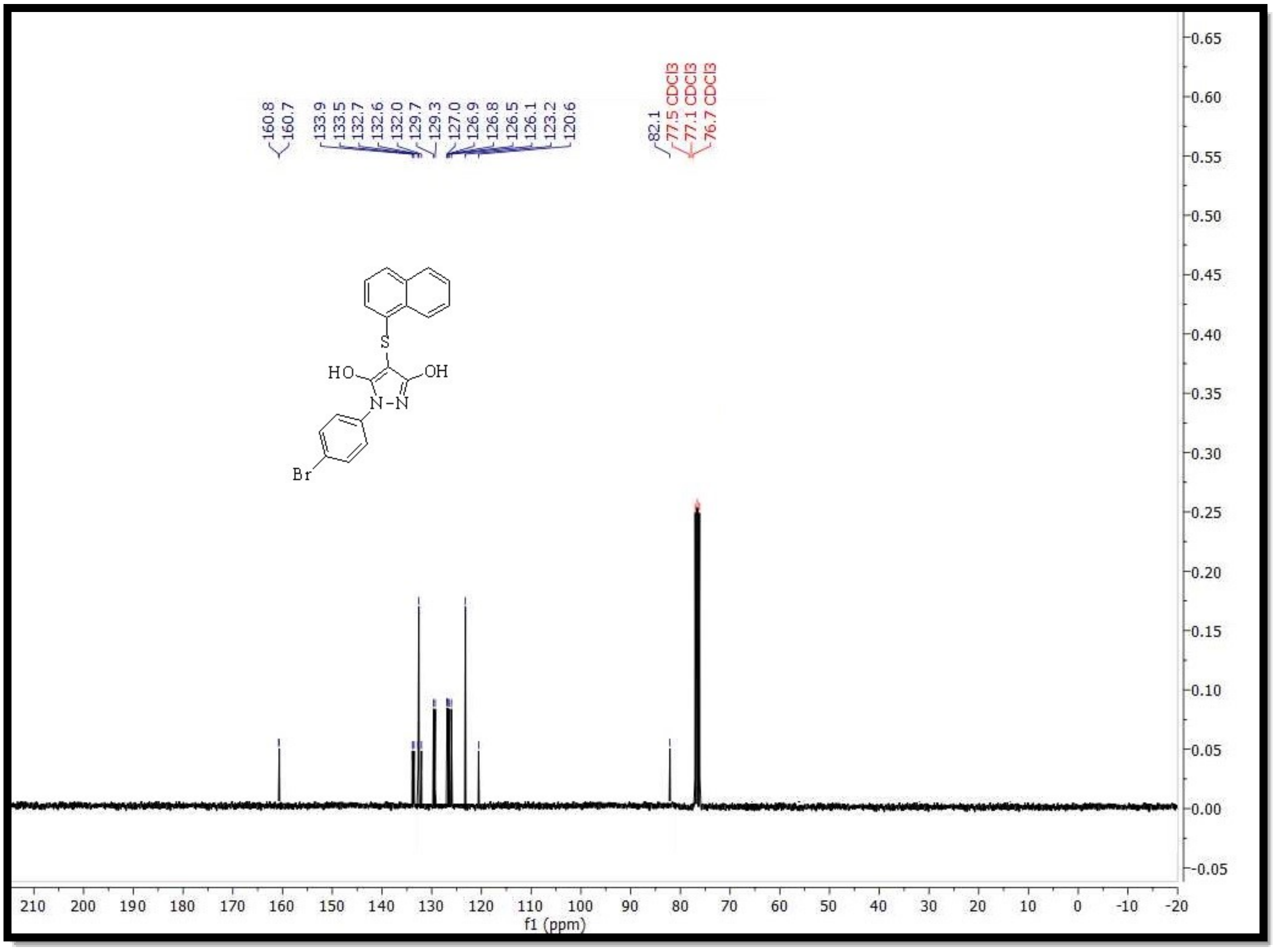

Figure 72: ^{13}C-NMR spectrum of compound **6i**

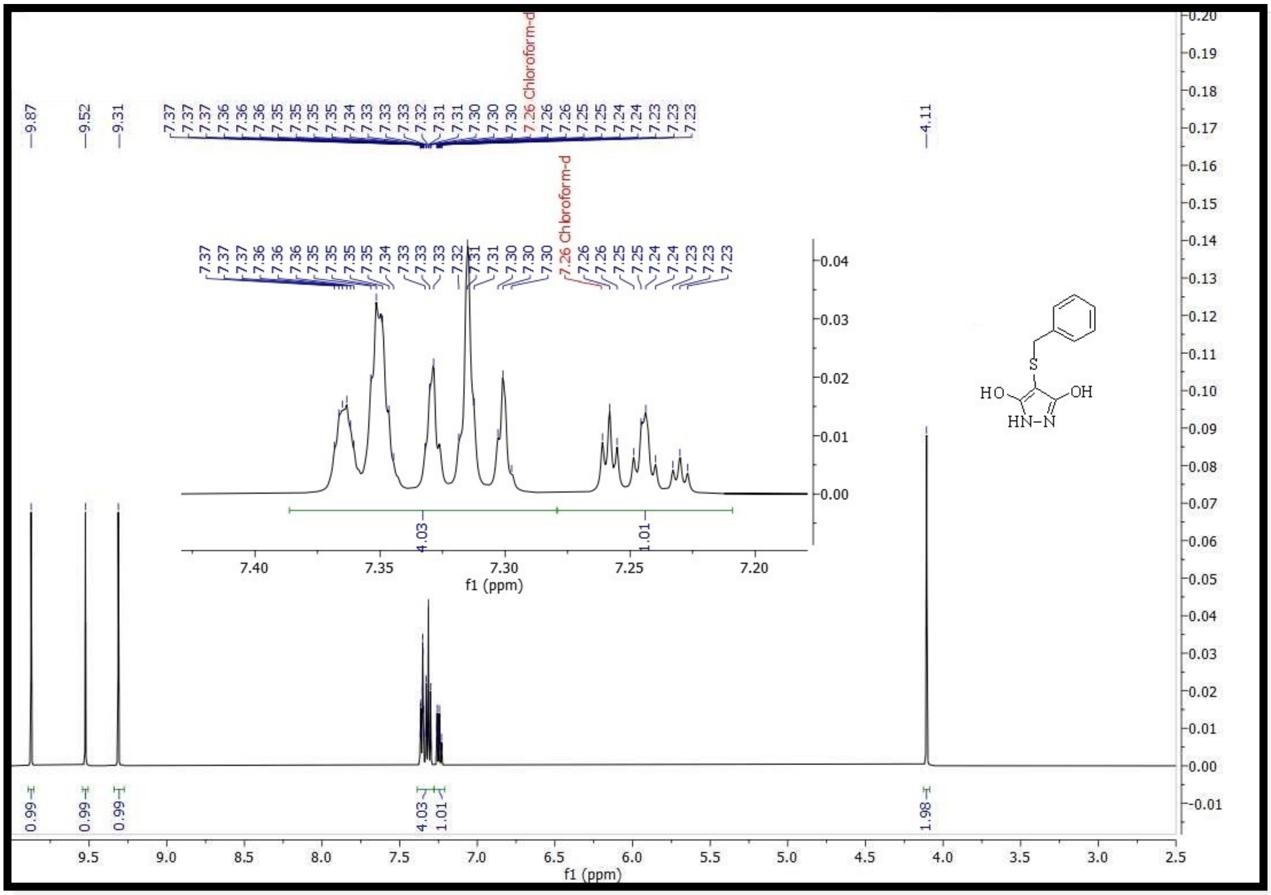

Figure 73: ^{1}H-NMR spectrum of compound **6j**

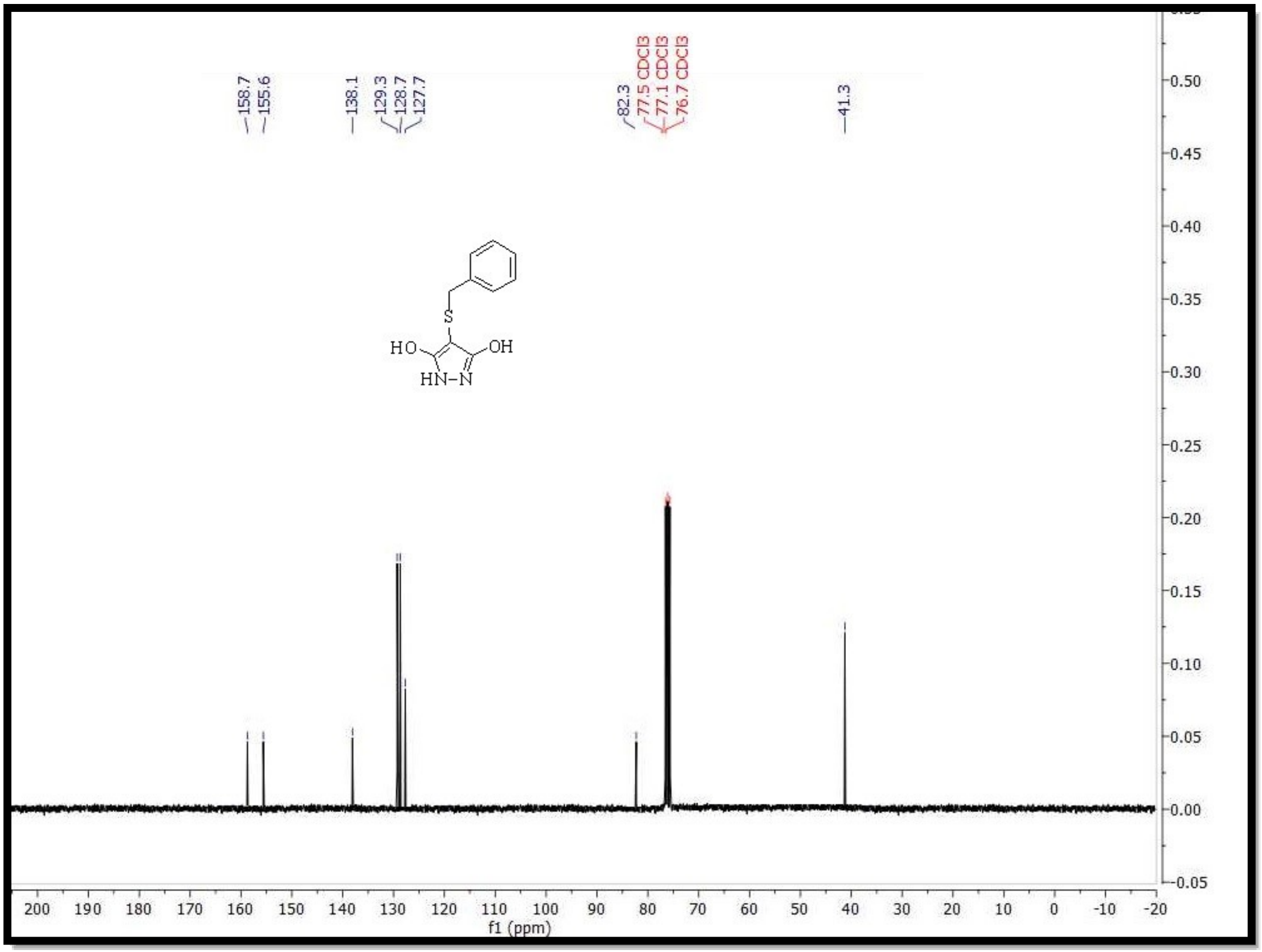

Figure 74: ^{13}C-NMR spectrum of compound **6j**

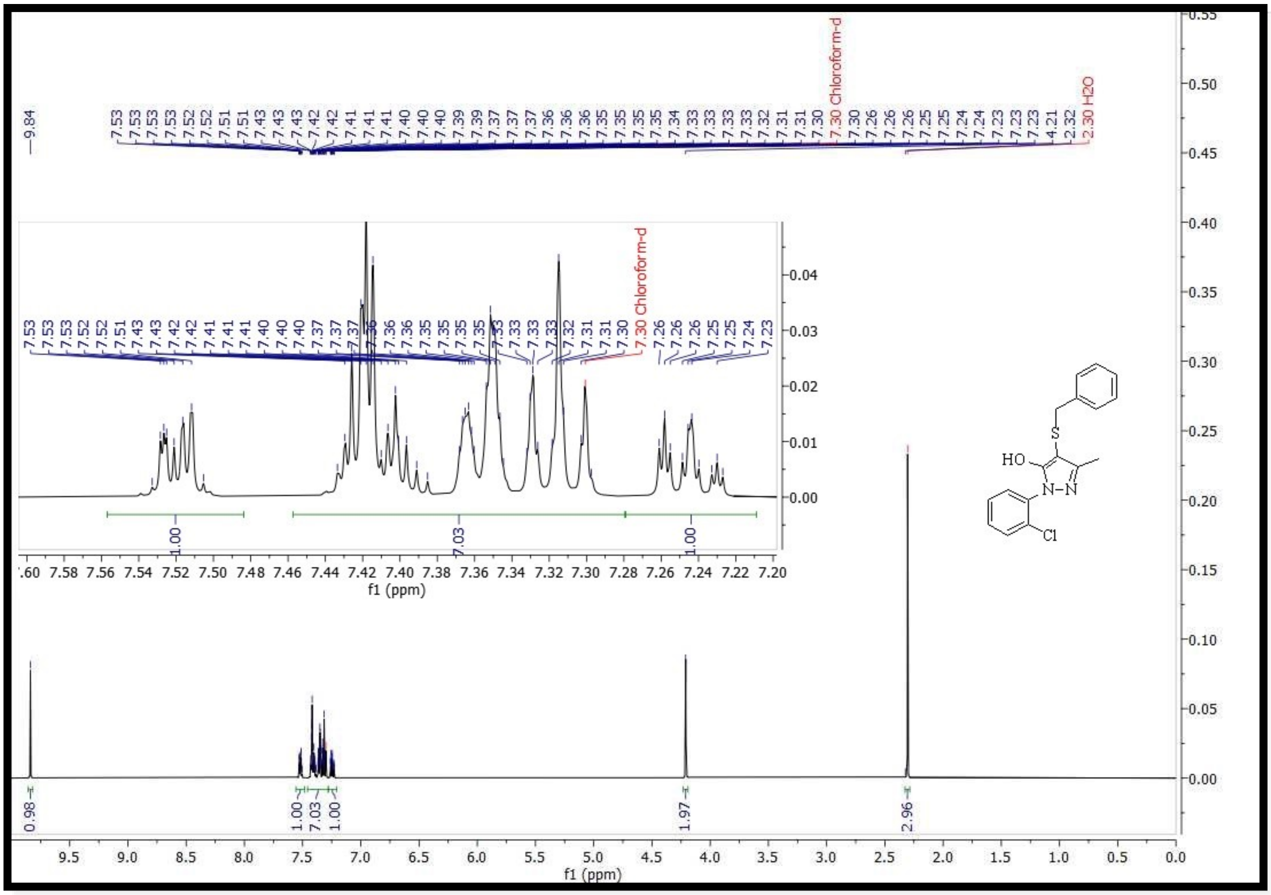

Figure 75: ^{1}H-NMR spectrum of compound **6k**

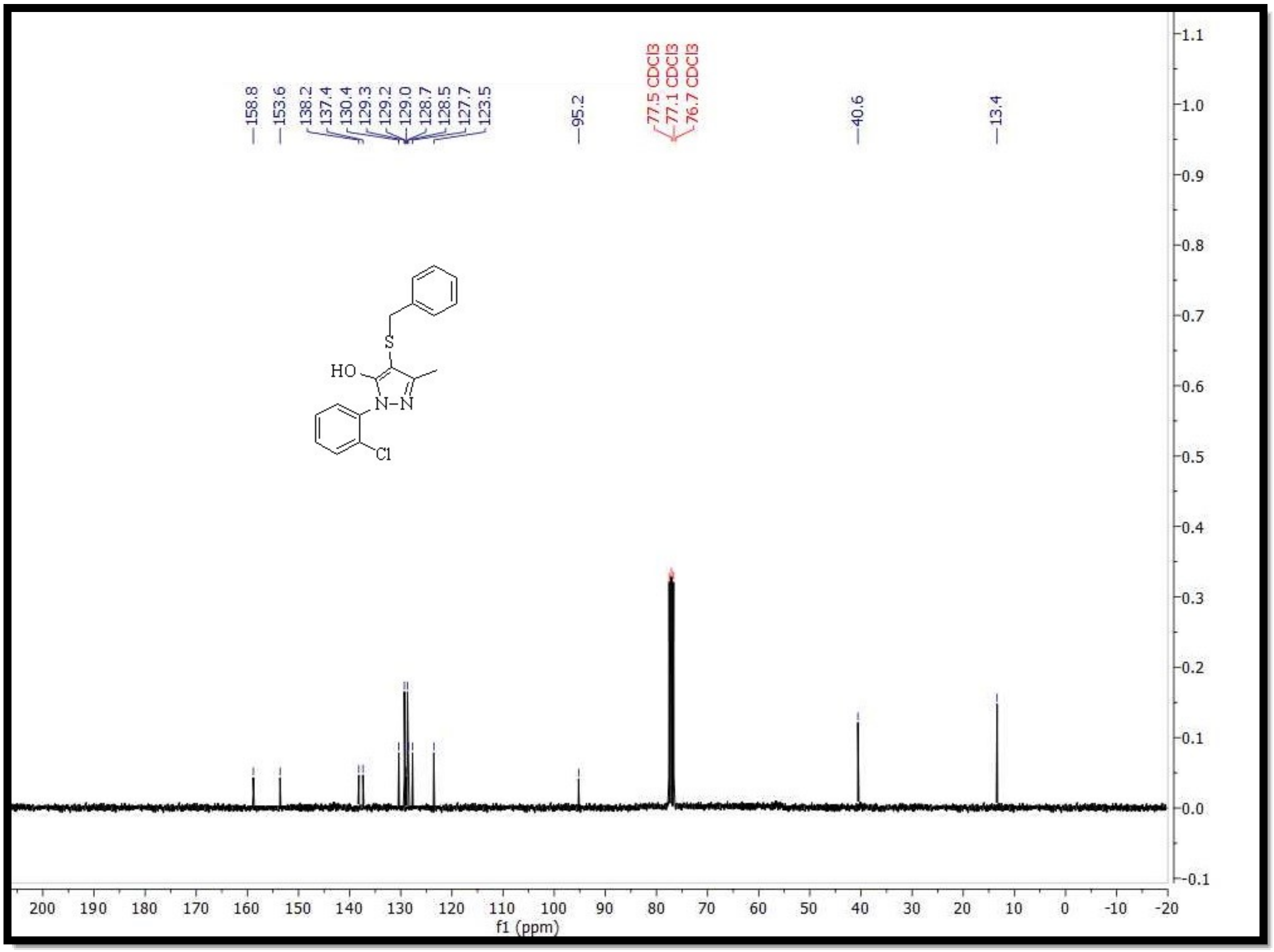

Figure 76: ^{13}C-NMR spectrum of compound **6k**

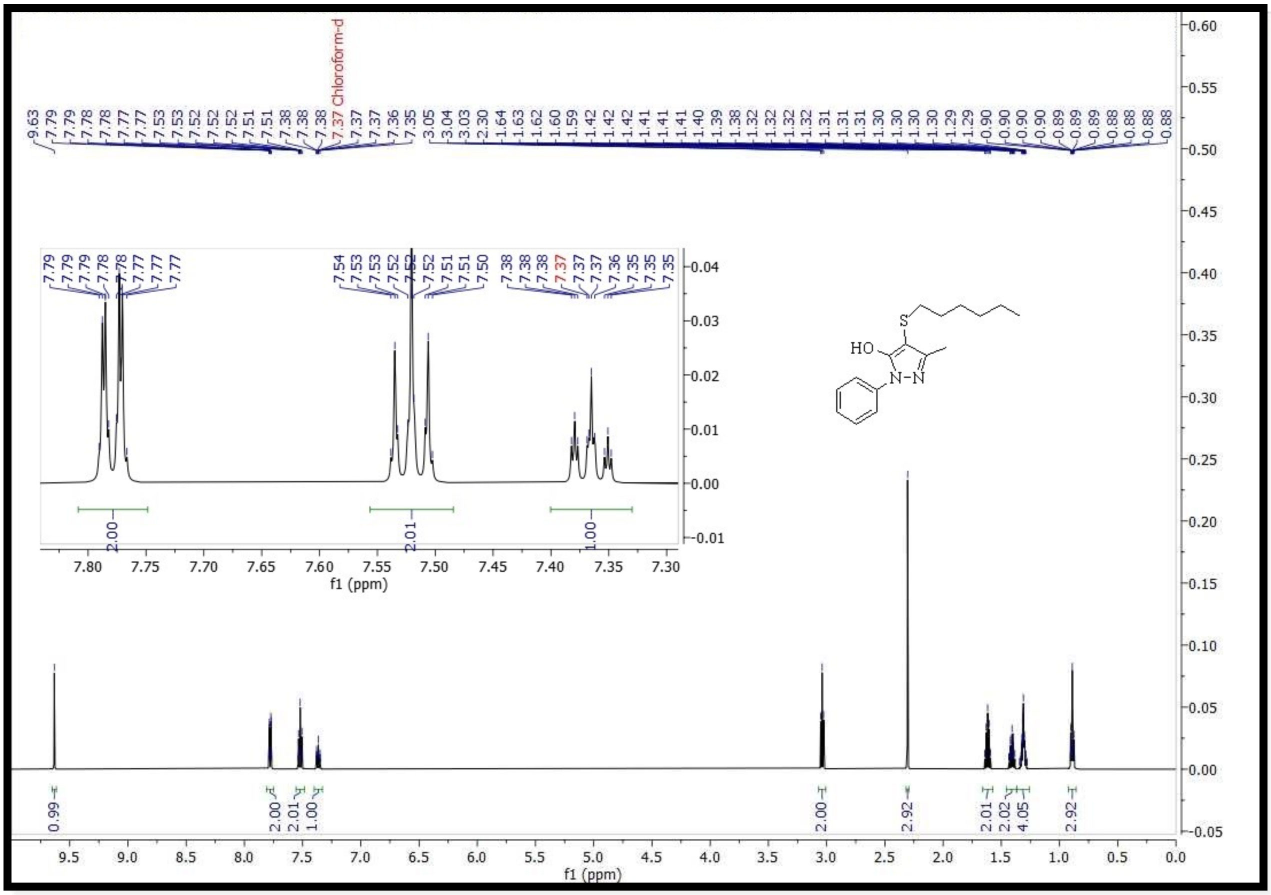

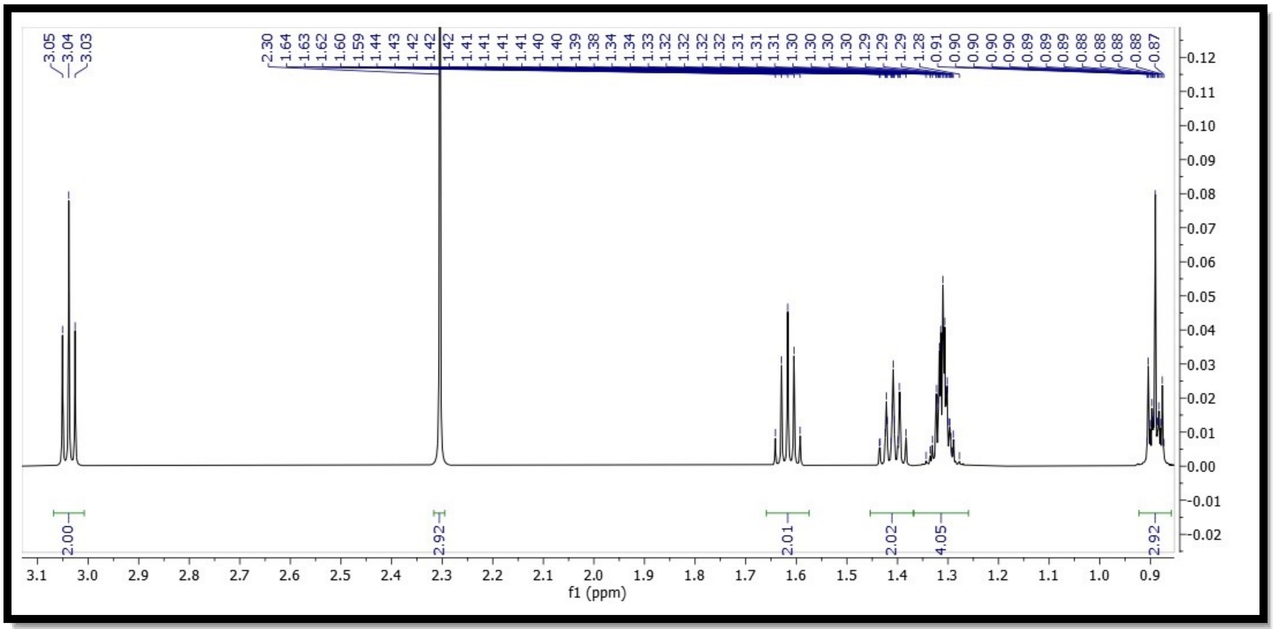

Figure 77: ^{1}H-NMR spectrum of compound **6l**

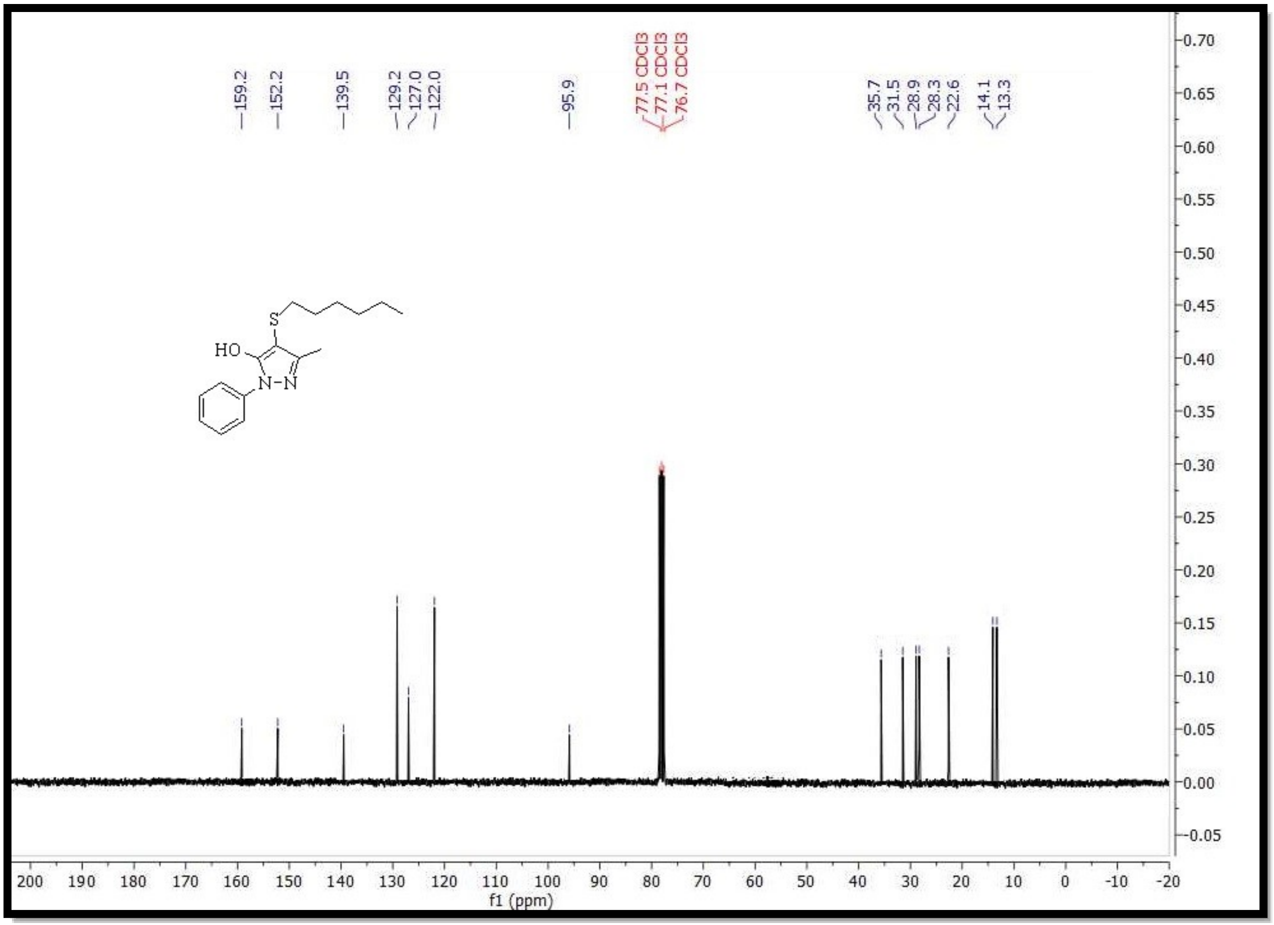

Figure 78: ^{13}C-NMR spectrum of compound **6l**
